# Supplementary material for: Older adults’ medical preferences for the end of life: a cross-sectional population-based survey in Switzerland
Source: BMJ Open. 2023 Jul 24;13(7):e071444. doi: 10.1136/bmjopen-2022-071444 (PMC10373667; doi:10.1136/bmjopen-2022-071444)
Supplement: Supplementary data [file bmjopen-2022-071444supp001.pdf]

FMH

SAMW ASSM
Schweizerische Akademie der Medizinischen Wissenschaften
Académie Suisse des Sciences Médicales
Accademia Svizzera delle Scienze Mediche
Swiss Academy of Medical Sciences

Advance directive | Detailed version

Drawn up by

Name, first name ______________________________

Date of birth ______________________________

Place of residence ______________________________

1. This advance directive is applicable in the following situations

I am drawing up this advance directive after careful reflection in case, at some point, I am no longer able to express my wishes as a result of illness or an accident. The advance directive is to be applicable:

☐ in all situations where I am incapacitated and decisions on medical treatment have to be taken – i.e. in the case of unexpected acute events such as a heart attack, stroke or accident, but also in advanced stages of chronic disease;

or

☐ ______________________________

____

2. My motivation and personal values

The following description of my motivation and personal values is a product of careful reflection and is intended to guide the health care team in the event of any difficulties of interpretation.

The following specific situation has prompted me to draw up this advance directive:

☐ (description, if appropriate) ______________________________

____

☐ No specific situation; this is a precautionary measure in case I should become incapacitated.

What I particularly wish to achieve with this advance directive is…

…that all possible medical measures are taken to keep me alive. My suffering should be relieved as far as possible, but I accept that certain burdens may be associated with the preservation of my life.<br>☐ This reflects my views more closely.	or	…that medical treatment should aim above all to relieve suffering. Prolonging life at all costs is not the priority for me. I accept that my life may be shortened if certain medical treatments are withheld.<br>☐ This reflects my views more closely.

Describe in your own words your motivation for drawing up an advance directive:

My current situation:

My attitude to life:

My experience, attitude and fears concerning disease, dying and death:

What I understand by 'quality of life'; what impairments/losses of independence I would find it difficult to accept:

Personal and/or religious convictions by which I am guided:

☐ As a member of the following religion, I wish to receive pastoral care:

☐ I do not wish to receive pastoral care.

Vilpert S, *et al. BMJ Open* 2023; 13:e071444. doi: 10.1136/bmjopen-2022-071444

3. Statements concerning treatment goals and specific medical measures

In drawing up this advance directive, I have received advice from

☐ My GP, Dr ______________________________________

☐ or ______________________________________

☐ I do not wish to give any instructions concerning specific medical measures, but I would ask the health care team to act as far as possible in accordance with my wishes (see Section 2: 'My motivation and personal values').

☐ I wish to give specific instructions for the following situations (cf. choices I - IV).

I Unexpected acute event (e.g. accident, stroke, heart attack)

If I am incapacitated as a result of an unexpected acute event and – after initial emergency measures and careful medical assessment – it is deemed impossible or unlikely that I will regain capacity,

☐ I wish to forgo all measures which would serve merely to prolong life and suffering.

☐ I wish that, despite the poor outlook, every medically appropriate measure should be taken.

II Treatment of pain and other symptoms

☐ In any event, I wish to receive effective treatment of pain and other distressing symptoms such as anxiety, restlessness, breathing difficulties and nausea. I am prepared to accept the reduced awareness (sedation) which such treatment may induce.

or

☐ For me, alertness and the ability to communicate are more important than optimum relief of pain and other symptoms.

III Artificial hydration and nutrition

I consent to *long-term* artificial hydration and nutrition (by nasogastric tube, infusion, surgically implanted feeding tube).

☐ Yes ☐ No

If you answered no

I consent to artificial hydration and nutrition *on a temporary basis*, i.e. if it is expected that my suffering will thereby be relieved or that I will subsequently be able – possibly with support – to ingest fluids and food by the normal route again.

☐ Yes ☐ No

IV Resuscitation in the event of a cardiac and/or respiratory arrest

I wish to be resuscitated.

☐ Yes ☐ No

Additional comments:

____

____

____

____

4. Trusted person / healthcare proxy

☐ I have appointed the following healthcare proxy whom I authorize to make my wishes known to the health care team. This person is to be informed about my condition and involved in decision-making; he/she can access my medical records, and I release all physicians and nursing staff from their duty of confidentiality vis-à-vis this person.

Name, first name ______

Address ______

Postcode/town ______

Tel. home ______ work ______ mobile ______

E-mail ______

If my healthcare proxy cannot be contacted or is unable for other reasons to exercise this responsibility, I designate the following substitute:

Name, first name ______

☐ I have informed my healthcare proxy about this advance directive.

Name, first name of my general practitioner (GP) ______

Address ______

Postcode/town ______

Tel. home ______ work ______ mobile ______

E-mail ______

☐ I have informed my general practitioner (GP) about this advance directive.

5. Special instructions in the event of my death

Organ donation

☐ I wish to donate my organs, and I consent to the removal of any organs, tissue or cells and to the implementation of the preparatory medical measures which are required prior to organ donation. I consent to the use of any intensive measures this may require.

☐ I consent only to the removal of ______

☐ I do not wish to be a donor.

Autopsy: I consent to an autopsy

☐ Yes ☐ No ☐ I leave this decision to my healthcare proxy.

I am aware that the drawing-up of an advance directive is voluntary, and that I may revoke or modify it at any time, as long as I have mental capacity.

I have prepared separate supplements to this advance:

☐ No ☐ Yes, namely ______

Place, date ______ Signature ______

N.B. Please keep your advance directive in such a way that it can be found should the need arise; you can leave a copy with your general practitioner. Advance directives should not be sent to the FMH/SAMS.The SAMS guidelines on advance directives are available online at *www.samw.ch*, and further information on advance directives is available at *www.fmh.ch > Patientenverfügung.*

FMH

SAMW ASSM
Schweizerische Akademie der Medizinischen Wissenschaften
Académie Suisse des Sciences Médicales
Accademia Svizzera delle Scienze Mediche
Swiss Academy of Medical Sciences

Advance directive | Short version

Drawn up by

Name, first name ____________________

Date of birth ____________ Place of residence ____________________

If I become incapacitated, it is my wish that:

☐ all medically indicated measures (including resuscitation) should be taken to treat the acute condition so that I may regain capacity;

or

☐ I should not be resuscitated and no intensive medical interventions (in particular ventilation) should be carried out;

or

☐ I should not be resuscitated, but I consent to treatment in an intensive care unit.

If, after an initial stabilizing of my condition, it becomes clear that I am unlikely to regain capacity and that there is a high risk that I will require long-term care, it is my wish that:

☐ all life-sustaining measures continue to be carried out, provided there is still hope that I may regain capacity;

☐ no further life-sustaining measures are carried out.

☐ In any event, I wish to receive effective treatment of pain and other distressing symptoms such as anxiety, restlessness, breathing difficulties and nausea.

I have appointed the following healthcare proxy whom I authorize to make my wishes known to the health care team. This person is to be informed about my condition and involved in decision-making; he/she can access my medical records, and I release all physicians and nursing staff from their duty of confidentiality vis-à-vis this person.

Name, first name ____________________

Address ____________________ Postcode/town ____________

Tel. home ____________ work ____________ mobile ____________

E-mail ____________________

If my healthcare proxy cannot be contacted or is unable for other reasons to exercise this responsibility, I designate the following substitute:

Name, first name ____________________

☐ I have informed my healthcare proxy about this advance directive.

Name, first name of my general practitioner (GP) ____________________

Address ____________________ Postcode/town ____________

Tel. home ____________ work ____________ mobile ____________

E-mail ____________________

☐ I have informed my general practitioner (GP) about this advance directive.

(Please turn over)

Vilpert S, *et al. BMJ Open* 2023; 13:e071444. doi: 10.1136/bmjopen-2022-071444

Organ donation

☐ I wish to donate my organs, and I consent to the removal of any organs, tissue or cells and to the implementation of the preparatory medical measures which are required prior to organ donation. I consent to the use of any intensive measures this may require.

☐ I consent only to the removal of ____________________

☐ I do not wish to be a donor.

Place, date ____________________ Signature ____________________

N.B. Please keep your advance directive in such a way that it can be found should the need arise. You can leave a copy with your general practitioner. Advance directives should not be sent to the FMH/SAMS. The SAMS guidelines on advance directives are available online at *www.samw.ch*, and further information on advance directives is available at *www.fmh.ch > Patientenverfügung*.

Vilpert S, *et al. BMJ Open* 2023; 13:e071444. doi: 10.1136/bmjopen-2022-071444
