# Supplementary material for: Older adults’ medical preferences for the end of life: a cross-sectional population-based survey in Switzerland
Source: BMJ Open. 2023 Jul 24;13(7):e071444. doi: 10.1136/bmjopen-2022-071444 (PMC10373667; doi:10.1136/bmjopen-2022-071444)
Supplement: Author's manuscript [file bmjopen-2022-071444.draft_revisions.pdf]

BMJ Open is committed to open peer review. As part of this commitment we make the peer review history of every article we publish publicly available.

When an article is published we post the peer reviewers' comments and the authors' responses online. We also post the versions of the paper that were used during peer review. These are the versions that the peer review comments apply to.

The versions of the paper that follow are the versions that were submitted during the peer review process. They are not the versions of record or the final published versions. They should not be cited or distributed as the published version of this manuscript.

BMJ Open is an open access journal and the full, final, typeset and author-corrected version of record of the manuscript is available on our site with no access controls, subscription charges or pay-per-view fees (http://bmjopen.bmj.com).

If you have any questions on BMJ Open's open peer review process please email info.bmjopen@bmj.com

Older adults' medical preferences for the end of life: a cross-sectional representative survey in Switzerland

Journal:	*BMJ Open*
Manuscript ID	bmjopen-2022-071444
Article Type:	Original research
Date Submitted by the Author:	27-Dec-2022

Complete List of Authors:	Vilpert, Sarah; University of Lausanne, Faculty of Business and Economics (HEC)<br>Meier, Clément; University of Lausanne, Faculty of Business and Economics (HEC); University of Lausanne, Faculty of Biology and Medicine<br>Berche, Jeanne; Paris-Dauphine University<br>Borasio, Gian Domenico; Centre Hospitalier Universitaire Vaudois, Palliative Care Service<br>Jox, Ralf; CHUV,<br>Maurer, Jurgen; University of Lausanne,

Keywords:	Quality of Life, PUBLIC HEALTH, Adult palliative care < PALLIATIVE CARE, PAIN MANAGEMENT, INTENSIVE & CRITICAL CARE

SCHOLARONE™
Manuscripts

BMJ

I, the Submitting Author has the right to grant and does grant on behalf of all authors of the Work (as defined in the below author licence), an exclusive licence and/or a non-exclusive licence for contributions from authors who are: i) UK Crown employees; ii) where BMJ has agreed a CC-BY licence shall apply, and/or iii) in accordance with the terms applicable for US Federal Government officers or employees acting as part of their official duties; on a worldwide, perpetual, irrevocable, royalty-free basis to BMJ Publishing Group Ltd ("BMJ") its licensees and where the relevant Journal is co-owned by BMJ to the co-owners of the Journal, to publish the Work in this journal and any other BMJ products and to exploit all rights, as set out in our licence.

The Submitting Author accepts and understands that any supply made under these terms is made by BMJ to the Submitting Author unless you are acting as an employee on behalf of your employer or a postgraduate student of an affiliated institution which is paying any applicable article publishing charge ("APC") for Open Access articles. Where the Submitting Author wishes to make the Work available on an Open Access basis (and intends to pay the relevant APC), the terms of reuse of such Open Access shall be governed by a Creative Commons licence – details of these licences and which Creative Commons licence will apply to this Work are set out in our licence referred to above.

Other than as permitted in any relevant BMJ Author's Self Archiving Policies, I confirm this Work has not been accepted for publication elsewhere, is not being considered for publication elsewhere and does not duplicate material already published. I confirm all authors consent to publication of this Work and authorise the granting of this licence.

For peer review only

Older adults' medical preferences for the end of life: a cross-sectional representative survey in Switzerland

Abstract:

Objectives: Advance directives (ADs) allow individuals to specify preferences for medical treatment in the event of decision-making incapacity. Our study investigates older adults' preferences toward three medical treatments that are frequently included in AD forms and their social, regional, and health patterns.

Setting: An observational study using nationally representative data of wave 8 (2019/20) of the Swiss component of the Survey of Health, Ageing, and Retirement in Europe (SHARE).

Participants: 1,430 adults aged 58 years and older living in Switzerland.

Primary and secondary outcome measures: Three vignette-based questions on the preferences regarding cardiopulmonary resuscitation (CPR), life-prolonging treatment in case of high risk of permanent mental incapacity, and reduced awareness as a consequence of analgesic medication and their associations with individuals' social, regional and health characteristics.

Results: Most older adults expressed the wish to receive CPR (65%), to forgo life-prolonging treatment in case of permanent mental incapacity (90.7%) and accepted reduced awareness if necessary for adequate treatment of pain and distressing symptoms (60.8%). Older adults' treatment preferences for CPR and life-prolonging treatment differed according to some social, regional, and health characteristics, while preference for reduced awareness was more uniformly distributed across population groups.

Conclusions: Simultaneous preferences for CPR and refusal of life-prolonging treatment can appear contradictory and fail to provide clear medical guidance to physicians who must implement ADs. The presence of conflicting medical information in ADs could be avoided by introducing relevant questions with clear and precise wording and educating the general public about end-of-life medical issues and the nature and limitations of available treatments.

Strengths and limitations of this study

- Nationally representative sample of older adults
- Quantitative method, multivariable regression models

Keywords

Advance directives; life-prolonging treatments; cardiopulmonary resuscitation; sedation; population-based study

For peer review only

Introduction:

Advances in medical technologies and extended life expectancy have altered the context of dying in our society (1) and increased the need to make complex medical decisions at the end of life (EOL) (2,3). EOL decisions mostly concern symptom management, withholding or withdrawing life-supporting treatment, and, in countries where it is legal, euthanasia and assisted suicide (4,5). Life-supporting treatment measures include, among others, cardiopulmonary resuscitation (CPR), artificial nutrition and hydration, artificial ventilation, and antibiotics. Following the principle of patient autonomy, patients are expected to make decisions about their treatment based on the information provided by physicians (6). So, patients' preference for whether or not to attempt CPR is commonly discussed in hospitals, nursing homes, ambulance services, and primary care consultations. In some health conditions, the decision to start, continue, withdraw or forgo treatment depends primarily on whether the patient wishes to maximize the quantity of life (aggressive treatments) or quality of life (comfort care), both goals being difficult to achieve simultaneously (7). The patient's medical choice can also be about the trade-off between benefits and consequences of treatment: e.g., the level of sedation the patient accepts in order to alleviate intractable suffering (i.e., mild, intermittent, terminal sedation) (8) . Patients can make these medical decisions when the situation arises, but at the end of life, more than half of the patients lose decision-making capacity (4) which often prevents individuals from formulating and expressing their wishes (9).

To enable the expression of medical treatment preferences in the event of a loss of decision-making capacity, it is possible to complete a written document that records the medical treatments to which a person consents or refuses and/or that designates a healthcare proxy to make medical decisions on behalf of that patient (10) which is commonly referred to as advance directives (ADs). ADs ensure patients' autonomy and self-determination; they enable patients to receive medical treatments that are better aligned with their wishes and thus contribute to maintaining their quality of life (11,12). In addition, ADs help family and healthcare specialists make the most appropriate medical decisions on the patient's behalf (13,14) and lower the stress related to surrogate medical decision-making (15).

In Switzerland, individuals can find numerous AD forms published by medical associations, patient organizations or other private initiatives, almost all of which include information on medical treatment preferences and the option to designate a healthcare proxy (16). ADs can be completed by individuals alone or with the assistance of trained professionals or healthcare specialists. ADs are often associated with death and older age, yet their completion is also recommended for healthy and/or young populations since they are targeted toward situations of medical care needs with lost of decision-making capacity (17).

For peer review only - http://bmjopen.bmj.com/site/about/guidelines.xhtml

Preferences for specific life-sustaining treatments (18–20), for general EOL care goals such as priority for length or quality of life (19,21,22), and palliative sedation therapy (23–26) have been studied in the general population, mostly in hypothetical contexts of severe physical pain or cognitive decline. To our knowledge, these preferences have not been investigated together and in a decontextualized manner, as they are presented in AD forms. Individuals who write ADs usually answer questions about preferences for certain treatments by referring either to their current health situation or to a hypothetical illness they imagine. We sought to replicate this ecological situation by asking three questions from one of the most widely known and used AD form in Switzerland (27) about preferences for treatments options to a nationally representative sample of adults aged 58 years and older in Switzerland. In this study, we examine older adults' preferences for CPR, life-prolonging treatment, and reduced awareness. We also investigate the association between treatments' preferences and individuals' social, regional, and health characteristics. This information is relevant to understanding how older adults view specific EOL treatments and identifying differences in treatment preferences among the population groups.

Methods:

Data for this study come from the Survey of Health, Ageing and Retirement in Europe (SHARE), which collects internationally comparable longitudinal microdata on public health and socio-economic conditions of European aged 50 and over (28). We used a subsample of the Swiss-SHARE Wave 8 (2019/2020) dataset, including respondents who participated in the internationally harmonized in-person interview and the national paper-and-pencil self-administered EOL questionnaire. The EOL questionnaire concerns preferences, knowledge, attitudes, and behaviors toward EOL care planning. Its completion rate was 94,3%, representing 1,891 individuals. We only include adults aged 58 and older in our analyses since the last sample refreshment in Switzerland was in 2011, and therefore, respondents aged 50–57 in 2019/20 who could only enter SHARE as partners are not representative of the general population. So, after restricting the sample to respondents older than 58 years and older and eliminating respondents with missing values on the items used in the analysis, our final analytical selection had 1,430 respondents (Figure1).

(Insert figure 1 here)

Measures

Outcome variables:

The following vignettes have been adapted directly from the long version of the AD form publicly available for download from the Swiss Medical Association (FMH website (27). The adaptation mainly consisted in

For peer review only - http://bmjopen.bmj.com/site/about/guidelines.xhtml

transforming the statements into questions, keeping the vocabulary used in the English version of the form and the contextual information provided.

Vignette 1. *Imagine that you experience a cardiac and/or respiratory arrest. In this situation, you wish: to be resuscitated / not to be resuscitated (0=refuse CPR, 1=accept CPR).*

Vignette 2. *Imagine that you are incapacitated following an accident, a stroke, or a heart attack. After initial emergency measures and careful medical assessment, physicians deem it very unlikely that you will regain mental capacity. In this situation, you prefer: to forgo all measures which would only serve to prolong your life and suffering / that, despite the poor outlook, every medically appropriate measure should be taken (0=refuse life-prolonging treatment, 1=accept life-prolonging treatment).*

Vignette 3. *Imagine that you suffer from a disease that causes unbearable pain and symptoms such as fear, restlessness, breathing difficulties, and nausea. In this situation: you wish to receive optimum treatment of pain and other distressing symptoms, and you are prepared to accept the reduced awareness (sedation) that such treatment may induce / for you, alertness and the ability to communicate are more important than optimal relief of pain and other symptoms (0=refuse reduced awareness, 1=accept reduced awareness).*

Independent variables:

Gender (0=male, 1=female), age group (58-64 years, 65-74 years, 75+ years), education levels based on the International Standard Classification of Education (ISCED) of 2017 (29) (basic=ISCED levels 0-1-2, secondary=ISCED levels 3-4, tertiary=ISCED levels 5-6), living with a partner (0=has a partner, 1=has no partner), parenthood (0=no children, 1=at least one child), self-perceived financial difficulties (1=easily, 2=fairly easily, 3=with difficulty), living environment (0=urban, 1=rural), language regions (German, French or Italian) and religiosity (0=never pray, 1=pray at least sometimes), self-rated health (1 = poor/fair health, 2 = good health, 3 = very good/excellent health), and self-reported limitation in activities of daily living (ADLs) (0=no limitation, 1=at least one limitation).

Statistical analysis:

The proportions of the variables included in our study are weighted using cross-sectional weights from the SHARE data (30). We performed probit regressions to assess the partial associations of preferences for medical treatments with respondents' characteristics. Regression estimates are expressed in terms of average partial effects (APEs). APEs report how the average conditional outcome probability $P(Y=1 \mid X)$ changes when the given explanatory variable changes from 0 to 1, holding all other independent variables at their observed values. Estimated standard errors were clustered at the household level to account for potential unobserved dependencies between partners'

For peer review only - http://bmjopen.bmj.com/site/about/guidelines.xhtml
For peer review only

responses. The estimations were conducted using STATA/SE 17.0 software (STATA Corporation, College Station, TX).

Ethical approval

The study was approved by the research ethics committee of the canton of Vaud in March 2014 with number 66/14. In addition, during data collection, respondents gave their consent to participate in the survey on two occasions, when they agreed over the phone to schedule an interview and at the beginning of the in-person interview.

Patient and Public Involvement

Patients and the public were not directly involved in the design of this study as their contribution would not have been relevant to the research question.

Results:

The social, cultural, and health characteristics of the study population are presented in Table 1. Regarding preferences for medical treatments displayed in Figure 2, 65% of respondents preferred to receive CPR, 90.7% chose to forgo life-prolonging treatment in case of high risk of permanent mental incapacity, and 60,8% accepted reduced awareness for optimum treatment of pain and other distressing symptoms.

For peer review only

Table 1: Characteristics of the study population, adults aged 58+, SHARE Switzerland, 2019/2020, n=1,430		
	Weighted proportion (%)	95% confidence interval
Gender		
Male	53.5	[49.0-58.0]
Female	46.5	[42.0-51.0]
Age groups		
58-64 years	50.9	[45.5-56.2]
65-74 years	27.1	[23.8-30.5]
75+ years	22.0	[19.1-24.9]
Education		
Low	14.2	[11.0-17.4]
Secondary	65.1	[60.3-69.8]
Tertiary	20.7	[16.4-25.0]
Living with a partner		
Yes	70.5	[65.8-75.3]
No	29.5	[24.7-34.2]
Parenthood		
No children	16.9	[12.6-21.2]
At least on child	83.1	[78.8-87.4]
Self-perceived financial difficulties		
Easily	58.5	[53.5-63.4]
Fairly easily	30.1	[25.6-34.5]
With difficulty	11.5	[8.4-14.5]
Living environment		
Urban	41.6	[36.5-46.7]
Rural	58.4	[53.3-63.5]
Switzerland linguistic regions		
German	72.7	[67.8-77.5]
French	24.7	[19.9-29.5]
Italian	2.6	[1.5-3.7]
Religiosity		
Never pray	29.6	[24.9-34.2]
Pray at least sometimes	70.4	[65.8-74.1]
Self-rated health		
Poor/fair health	15.2	[11.9-18.5]
Good health	39.7	[34.8-44.6]
Very good/excellent health	45.1	[39.7-50.4]
Self-reported limitations in activities of daily living (ADLs)		
No limitation	94.7	[92.8-96.6]
At least one limitation	5.3	[3.4-7.2]

(Insert Figure 2 here)

For peer review only - http://bmjopen.bmj.com/site/about/guidelines.xhtml
For peer review only

Table 2 shows the results from the probit regressions of preferences for medical treatment on respondents' characteristics. First, concerning preferences for CPR, being a woman (APE: -11.2, $p < 0{,}001$), living without a partner (APE: -8.2; $p < 0{,}05$), and being older than 64 years old (APE 65-74: -10.1, $p < 0{,}01$; APE 75+: -20.4, $p < 0{,}001$) were each associated with a lower likelihood of wanting to be resuscitated. In contrast, having a tertiary level of education (APE: 10.3, $p < 0{,}05$), being from the Italian-speaking part of Switzerland (APE: 24.8, $p < 0{,}001$), praying at least sometimes (APE: 5.6, $p < 0{,}01$), and self-rating one's health as good (APE: 9.4, $p < 0{,}05$) very good/excellent (APE: 12.0, $p < 0{,}01$) were positively associated with willingness of CPR. Second, regarding the preference for life-prolonging treatment, being a woman (APE: -4.3, $p < 0{,}01$), having 65 to 74 years old (APE: -3.8, $p < 0{,}05$), and having at least one limitation in activities of daily living (APE: -4.8, $p < 0{,}05$) were all negatively associated with preference for life-prolonging treatment in case of high risk of permanent mental incapacity. On the other side, living without a partner (APE: 4.3, $p < 0{,}01$) and being from the Italian part of Switzerland (APE: 17.7, $p < 0{,}01$) were associated with a higher chance of wanting life-prolonging treatment. Third, concerning preferences for reduced awareness, having a secondary level of education (APE: 8.9, $p < 0{,}01$) was correlated with a higher likelihood of accepting reduced awareness for optimum pain treatment and other distressing symptoms. At the same time, respondents who said they pray at least sometimes (APE: -6.2, $p < 0{,}01$) were more likely to refuse reduced awareness.

For peer review only

For peer review only - http://bmjopen.bmj.com/site/about/guidelines.xhtml

Table 2: Partial associations of preferences for medical treatments with respondents' social, cultural, and health characteristics, adults aged 58+, SHARE Switzerland, 2019/2020, n=1,430			
	Preference for CPR APE (95% CI)	Preference for life-prolonging treatment APE (95% CI)	Preference for reduced awareness APE (95% CI)
Gender (ref: male)			
Female	-11.2*** (-16.2,-6.3)	-4.3** (-7.3,-1.4)	2.3 (-2.9,7.4)
Age groups (ref: 58-64 years)			
65-74 years	-10.1** (-16.3,-4.0)	-3.8* (-7.6,-0.1)	2.2 (-4.2,8.5)
75+ years	-20.4*** (-27.3,-13.5)	-4.0 (-8.0,0.0)	-5.1 (-12.4,2.2)
Education (ref: low)			
Secondary	1.2 (-6.0,8.4)	-4.1 (-8.8,0.7)	8.9* (1.4,16.3)
Tertiary	10.3* (1.5,19.1)	-4.1 (-9.5,1.4)	7.6 (-1.8,16.9)
Living with a partner (ref: yes)			
No	-8.2* (-14.4,-1.9)	4.3* (0.5,8.1)	2.2 (-4.1,8.5)
Parenthood (ref: no children)			
At least on child	0.6 (-6.5,7.7)	-0.6 (-4.5,3.2)	3.3 (-3.9,10.5)
Self-perceived financial difficulties (ref: easy)			
Fairly easily	1.6 (-4.2,7.4)	1.1 (-2.0,4.3)	-2.4 (-8.4,3.7)
With difficulty	2.2 (-6.0,10.3)	2.9 (-2.2,8.1)	-0.0 (-8.7,8.6)
Living environment (ref: urban)			
Rural	1.4 (-3.8,6.6)	0.3 (-2.5,3.0)	-0.0 (-5.4,5.3)
Switzerland linguistic regions (ref: German)			
French	-6.1 (-12.3,0.0)	2.3 (-1.1,5.7)	2.6 (-3.5,8.7)
Italian	24.8*** (15.2,34.3)	17.7* (4.0,31.4)	-7.5 (-23.5,8.5)
Religiosity (ref: never pray)			
Pray at least sometimes	5.6* (0.1,11.1)	0.5 (-2.5,3.5)	-6.2* (-11.8,-0.5)
Self-rated health (ref: poor/fair health)			
Good health	9.4* (1.8,16.9)	-2.4 (-6.6,1.8)	2.6 (-4.8,10.0)
Very good/excellent health	12.0** (4.0,20.0)	-1.9 (-6.3,2.5)	-3.3 (-11.4,4.7)
Self-reported limitations in activities of daily living (ADLs) (ref: no limitation)			
At least one limitation	-1.7 (-12.7,9.4)	-4.8* (-8.6,-0.9)	0.9 (-10.4,12.3)
Observations	1,430	1,430	1,430

*CPR=Cardiopulmonary resuscitation. Average partial effects (APEs) based on probit regression models. All probabilities are multiplied by 100. Asterisks indicate levels of significance: ***<0.1%, ** 1%, *5%. Interpretation of APEs: Being a woman decreases the probability of wanting to be resuscitated by 11.2 percentage points compared to being a man.*

For peer review only - http://bmjopen.bmj.com/site/about/guidelines.xhtml

Discussion

The present study is the first nationally representative population-based study in Switzerland investigating older adults' preferences toward medical treatments typically described in AD forms. Most respondents wished to receive CPR in case of a cardiac/respiratory arrest, to forgo life-prolonging treatment in case of high risk of permanent mental incapacity, and to reduce their awareness for optimum treatment of pain and other distressing symptoms. Additionally, social, regional, and health patterns in medical treatment preferences were found for CPR and life-sustaining treatments, while preference for reduced awareness was more uniformly distributed across population groups.

Preference for cardiopulmonary resuscitation

In our study, a majority of individuals reported wanting to receive CPR in the event of cardiac and/or respiratory arrest. In most population-based studies investigating the preference for CPR, the question is asked for a given health condition (e.g., terminal cancer, advanced dementia), and individuals largely refuse CPR (18,19,23). In our study, the context of the CPR is not specified, and the respondents certainly refer to their current health status, which is rather good (84.8% declared a good, very good, or excellent health). The preference for CPR may be influenced by the prospect of significant remaining life expectancy (22), but it could also be explained by an excessive optimism for the success of such an intervention. Indeed, the general public tends to strongly overestimate the survival rate of CPR, which leads individuals to desire CPR for themselves (31). This distorted image of CPR is driven by media, TV series and films (32), the general societal narrative of CPR as life-saving punctual measure that immediately restores the healthy state before (which is rarely the case (33)).

Regarding the social pattern of CPR preferences in our study, women reported less often the wish to be resuscitated than men. This finding, which has been shown in other studies (19,23), could be explained by the fact that women value quality of life over quantity (34) which may lead them to refrain from treatments (35) and that their direct experience with caregiving makes them aware of the benefits and limitations of medical treatments and the potential burden of a patient in relatives (22). Respondents living without a partner were also less likely to choose CPR than respondents living with a partner. Since respondents living without a partner are better informed about the limited success of CPR, as shown in a study based on the same population as our (36), they may prefer to refuse CPR (37).

Conversely, respondents with a tertiary level of education were more likely to indicate that they were willing to be resuscitated. Another study on the same population has shown that older adults with higher levels of education were not more knowledgeable about the success of CPR, so they would not be expected to be more likely to forgo

For peer review only - http://bmjopen.bmj.com/site/about/guidelines.xhtml
For peer review only

CPR than others (36). A study about hospitalized patients aged 65 and over in New Zealand reported that more educated patients were more likely to prefer CPR in their current state of health (38). One potential reason could be that individuals with higher education levels have are aware that they have better health prospects (39) which could induce them to choose CPR. In fact, one study showed that older adults who are optimistic about their own life expectancy are more likely to want to continue all medical treatment in a situation of serious illness with very low chance of survival (22). Two other variables associated with more acceptance of CPR were the Italian linguistic region and active religiosity (praying at least sometimes). These findings highlight well-documented cultural differences in Switzerland, with respondents from the Italian-speaking part (40,41) and those praying at least sometimes having CPR preferences likely influenced by religious prescriptions and the belief in the sanctity of life (35,41,42) that encourage choosing life-prolonging treatments (12).

Finally, age and self-rated health status, two parameters potentially influencing medical prognoses, were associated with CPR preferences. Compared to adults aged 58-64, respondents from the two older age categories were less likely to state that they wanted CPR, with the negative association being stronger with the increase in age. These results are consistent with other studies showing that older people were more likely to refuse CPR (19,23,43). Older adults might realize that age decreases their chances of surviving a CPR (44) and therefore prefer to reject this treatment. The same reasoning is certainly true for people in poorer health (45).

Preference for life-prolonging treatment

In our sample, over 90% of respondents decided to forgo life-prolonging treatment if they were to become incapacitated following an accident, a stroke, or a heart attack and were very unlikely to regain mental capacity. Similar results were found in studies of the wish to continue or stop treatment in the presence of serious physical or mental illness in the general population (19,22). Interestingly, the population groups more likely to forgo life-prolonging treatments are comparable to those who refused CPR: women, adults aged 65-74, and those in poorer health (1+ ADLs). These findings are in line with a study of the general Dutch population that evidenced that women, older people, and people with a history of serious illness prioritize quality of life over quantity (35). In contrast, Swiss participants from the Italian-speaking region who were more likely to prefer CPR were also more likely to prefer life-prolonging treatment.

A surprising result for which we have no explanation is the preference for life-sustaining treatments among people living without a partner, while more of them refused CPR.

Preference for reduced awareness

For peer review only - http://bmjopen.bmj.com/site/about/guidelines.xhtml

In our study, 60.8% of the respondents accepted reduced awareness in a situation where they would suffer from a disease that causes unbearable pain and symptoms such as fear, restlessness, breathing difficulties, and nausea, while for 39.2% of respondents, awareness and the ability to communicate was more important than optimal relief of pain and other symptoms. Other studies investigated preferences for palliative sedation therapy in the population with various definitions of sedation and health condition (physical, mental distress). In Quebec, 67.9% of adults aged 18+ declared to be in favor of continuous deep sedation for themselves with cancer or dementia (24). In Japan, 52% declared to want care without sedation in case of severe refractory physical distress, and 75% reported to request palliative sedation (23). Another Japanese study that nuanced the types of palliative sedation found that adults aged 18+ prefer intermittent or mild sedation to continuous deep sedation in the context of physical and psychological distress (25). Furthermore, in a Dutch study, 61% of adults aged 20+ reported being conscious until death as important, and those individuals were less to accept terminal sedation (67%) and the use of high dosages of morphine (67%) (26). These findings show that the preservation of intellectual activities plays a central role in achieving a good death (23,25).

Only education and religiosity were correlated with individuals' preferences for or against reduced awareness as a side effect of symptom treatment. A secondary education level was linked with a higher acceptance of reduced awareness. This is consistent with a Belgian study showing that patients at the end of life with higher levels of education are more likely to emphasize pain and symptom relief (46). Furthermore, more religiously active respondents were less likely to accept reduced awareness, which may be linked to the (erroneous but widespread) belief that symptom treatment that reduces awareness also shortens life, and religiosity is commonly associated with life-prolonging treatment preferences among older adults (19,22,35). In addition, religion often involves notions of spiritual awareness, which is necessary for praying and communicating with the divinities or their representatives, especially at the end of life. Thus, this religious value may be incompatible with the reduced awareness induced by some treatments to relieve pain and symptoms.

Potential discrepancy in treatment preferences

Our study showed results that may seem contradictory in that respondents did not want life-prolonging treatments but did want CPR, which has a relatively low chance of survival. This type of ambivalence has already been noted in a study that showed that about one-third of the Dutch adult population could not make a clear choice between quality and quantity of life (35). Another study pointed to inconsistencies between Dutch older adults' general EOL goals and preferences for specific life-sustaining treatments (18). The authors explained these contradictions by the population's lack of knowledge of the risks and side effects of these treatments and their distorted

For peer review only - http://bmjopen.bmj.com/site/about/guidelines.xhtml

perception. In our study, this apparent contradiction may be related to the fact that respondents established their preference for CPR by referring to their current state of health, whereas for life-prolonging treatment they presumed a severely reduced state of health. Nevertheless, conflicting information about individuals' treatment preferences may appear in AD forms, especially when the form's questions are ambiguous, decontextualized, or both. In these circumstances, ADs may become very difficult to interpret when making medical decisions.

Limitations

Our study presents some limitations. First, the overwhelming majority of respondents reporting a preference to refuse life-prolonging treatment suggests that the way this question is framed is not optimal, as it is not sufficiently discriminating and therefore does not provide clear guidance on which medical treatments are wished or not. in that it does not provide clear guidance on what medical decisions should be made. Second, although it is valuable to know the importance of remaining aware in patients at the end of life, it seems inappropriate to learn about this preference in patients who have lost their decision-making capacity. However, these two questions are directly drawn from the FMH AD form. Although this form contains other questions that may supplement the information provided, it seems desirable that these two questions be refined and clarified to be medically useful and actionable and not unnecessarily overload the decision-making process of the person completing ADs. Third, because our sample suffers from attrition problems common to longitudinal studies, people in very poor health or living in a nursing home may be underrepresented, although considerable efforts are undertaken in the SHARE survey to minimize these biases. On the other hand, the EOL questionnaire's response rate was extremely high (94.3%), and an analysis of the profile of non-respondents indicates that no population group was underrepresented.

Conclusion

Our study investigates preferences for CPR, life-prolonging treatments, and reduced awareness, three statements included in one of the most used Swiss AD forms in a nationally representative sample of adults aged 58 years and older living in Switzerland. Most older adults wished to receive CPR in case of a cardiac/respiratory arrest, forgo life-prolonging treatment in case of high risk of permanent mental incapacity, and reduce their awareness for optimum treatment of pain and other distressing symptoms. Our study also revealed that individuals with characteristics that may influence medical prognoses, such as advanced age and health conditions, as well as women were less likely to wish for CPR and life-prolonging treatments. These findings may indicate a more practical approach to the potential of medicine in circumstances such as EOL in these population groups. Furthermore, the apparent contradiction in our findings between, on the one hand, individuals' willingness to receive CPR and, on the other hand, their wishes to forgo life-prolonging treatment may reveal a general public's

For peer review only - http://bmjopen.bmj.com/site/about/guidelines.xhtml

For peer review only

lack of knowledge in the actual abilities of medicine and/or ambivalence about EOL goals and the medical decisions related to them. It is therefore essential to inform and educate the general population about common EOL medical situations and related treatment decisions before they are directly or indirectly exposed to them and to avoid treatment choices that do not reflect the individuals' actual preferences. In addition, public health policies should provide suitable guidance and support for advance care planning processes in order to ensure that ADs are tailored to the needs of the individuals who write them and the healthcare specialists who implement them.

Contributorship statement

SV designed the study. CM and JB conducted the statistical analysis with the support of SV. SV and CM drafted the manuscript. All authors discussed the interpretation of findings and provided critical revision of the manuscript for important intellectual content.

Competing interests

The authors declare that they have no competing interests.

Funding

This work was supported by the Swiss National Science Foundation (SNSF), grant number 10001C_188836.

This paper uses data from Börsch-Supan, A. (2021). Survey of Health, Ageing and Retirement in Europe (SHARE) Wave 8. Release version: 1.0.0. SHARE-ERIC. Data set. DOI: 10.6103/SHARE.w8.100. The SHARE data collection has been funded by the European Commission, DG RTD through FP5 (QLK6-CT-2001-00360), FP6 (SHARE-I3: RII-CT-2006-062193, COMPARE: CIT5-CT-2005-028857, SHARELIFE: CIT4-CT-2006-028812), FP7 (SHARE-PREP: GA N°211909, SHARE-LEAP: GA N°227822, SHARE M4: GAN°261982, DASISH: GA N°283646) and Horizon 2020 (SHARE-DEV3: GA N°676536, SHARE-COHESION: GA N°870628, SERISS: GA N°654221, SSHOC: GA N°823782) and by DG Employment, Social Affairs & Inclusion through VS 2015/0195, VS 2016/0135, VS 2018/0285, VS 2019/0332, and VS 2020/0313. Additional funding from the German Ministry of Education and Research, the Max Planck Society for the Advancement of Science, the U.S. National Institute on Aging (U01_AG09740-13S2, P01_AG005842, P01_AG08291, P30_AG12815, R21_AG025169, Y1-AG-4553-01, IAG_BSR06-11, OGHA_04-064, HHSN271201300071C, RAG052527A) and from various national funding sources is gratefully acknowledged (see www.share-project.org).

Data sharing statement

SHARE data are publicly available for scientific purposes only through registration on the data distribution platform.

For peer review only - http://bmjopen.bmj.com/site/about/guidelines.xhtml

Ethics Approval

The study was approved by the research ethics committee of the canton of Vaud in March 2014 with number 66/14.

References

1. Etkind SN, Bone AE, Gomes B, Lovell N, Evans CJ, Higginson IJ, et al. How many people will need palliative care in 2040? Past trends, future projections and implications for services. BMC Med. 2017 May 18;15(1):102.

2. Bosshard G, Zellweger U, Bopp M, Schmid M, Hurst SA, Puhan MA, et al. Medical End-of-Life Practices in Switzerland: A Comparison of 2001 and 2013. JAMA Intern Med. 2016 Apr;176(4):555–6.

3. Becker C, Künzli N, Perrig S, Beck K, Vincent A, Widmer M, et al. Code status discussions in medical inpatients: results of a survey of patients and physicians. Swiss Med Wkly. 2020 Mar 23;150(1314).

4. van der Heide A, Deliens L, Faisst K, Nilstun T, Norup M, Paci E, et al. End-of-life decision-making in six European countries: descriptive study. The Lancet. 2003 Aug 2;362(9381):345–50.

5. Löfmark R, Nilstun T, Cartwright C, Fischer S, van der Heide A, Mortier F, et al. Physicians' experiences with end-of-life decision-making: Survey in 6 European countries and Australia. BMC Med. 2008 Feb 12;6(1):4.

6. Principles of Biomedical Ethics - Paperback - Tom L. Beauchamp; James F. Childress - Oxford University Press [Internet]. [cited 2022 Nov 16]. Available from: //global.oup.com/ushe/product/principles-of-biomedical-ethics-9780190640873

7. Stiggelbout AM, De Haes JCJM, Kiebert GM, Kievit J, Leer JWH. Tradeoffs between Quality and Quantity of Life: Development of the QQ Questionnaire for Cancer Patient Attitudes. Med Decis Making. 1996 Jun 1;16(2):184–92.

8. Takla A, Savulescu J, Kappes A, Wilkinson DJC. British laypeople's attitudes towards gradual sedation, sedation to unconsciousness and euthanasia at the end of life. PLOS ONE. 2021 Mar 26;16(3):e0247193.

9. Silveira MJ, Kim SYH, Langa KM. Advance Directives and Outcomes of Surrogate Decision Making before Death. N Engl J Med. 2010 Apr 1;362(13):1211–8.

10. Pautex S, Herrmann F, Zulian G. Role of advance directives in palliative care units: a prospective study. Palliat Med. 2008 Oct 1;22(7):835–41.

11. Bosisio F, Jox RJ, Jones L, Truchard ER. Planning ahead with dementia: what role can advance care planning play? A review on opportunities and challenges. Swiss Med Wkly. 2018 Dec 30;(51).

12. Borrat-Besson C, Vilpert S, Borasio GD, Maurer J. Views on a "Good Death": End-of-Life Preferences

For peer review only - http://bmjopen.bmj.com/site/about/guidelines.xhtml

and Their Association With Socio-Demographic Characteristics in a Representative Sample of Older Adults in Switzerland. OMEGA - J Death Dying. 2020 Jul 29;

13. Detering KM, Hancock AD, Reade MC, Silvester W. The impact of advance care planning on end of life care in elderly patients: randomised controlled trial. BMJ. 2010 Mar 24;340:c1345.

14. van Wijmen MPS, Pasman HRW, Widdershoven GAM, Onwuteaka-Philipsen BD. Motivations, aims and communication around advance directives: A mixed-methods study into the perspective of their owners and the influence of a current illness. Patient Educ Couns. 2014 Jun 1;95(3):393–9.

15. Brinkman-Stoppelenburg A, Rietjens JA, van der Heide A. The effects of advance care planning on end-of-life care: A systematic review. Palliat Med. 2014 Sep 1;28(8):1000–25.

16. Rüegger H. Patientenverfügungen in der deutschsprachigen Schweiz, Institut Neumünster, CURAVIVA. 2011;45.

17. Otte I, Jung C, Elger B, Bally K. Advance directives and the impact of timing. A qualitative study with Swiss general practitioners. Swiss Med Wkly [Internet]. 2014 Sep 21 [cited 2022 Apr 1];(39). Available from: https://smw.ch/article/doi/smw.2014.14035

18. Evans N, Pasman HR, Deeg D, Onwuteaka-Philipsen B, EURO IMPACT. How do general end-of-life treatment goals and values relate to specific treatment preferences? a population-based study. Palliat Med. 2014 Dec;28(10):1206–12.

19. van Wijmen MPS, Pasman HRW, Widdershoven GAM, Onwuteaka-Philipsen BD. Continuing or forgoing treatment at the end of life? Preferences of the general public and people with an advance directive. J Med Ethics. 2015 Aug;41(8):599–606.

20. Hamano J, Hanari K, Tamiya N. End-of-life care preferences of the general public and recommendations of healthcare providers: a nationwide survey in Japan. BMC Palliat Care. 2020 Mar 24;19(1):38.

21. Chung RYN, Wong ELY, Kiang N, Chau PYK, Lau JYC, Wong SYS, et al. Knowledge, Attitudes, and Preferences of Advance Decisions, End-of-Life Care, and Place of Care and Death in Hong Kong. A Population-Based Telephone Survey of 1067 Adults. J Am Med Dir Assoc. 2017 Apr 1;18(4):367.e19-367.e27.

22. Carr D, Moorman SM. End-of-Life Treatment Preferences Among Older Adults: An Assessment of Psychosocial Influences. Sociol Forum Randolph NJ. 2009 Dec 1;24(4):754–78.

23. Sanjo M, Miyashita M, Morita T, Hirai K, Kawa M, Akechi T, et al. Preferences regarding end-of-life cancer care and associations with good-death concepts: a population-based survey in Japan. Ann Oncol Off J Eur Soc Med Oncol. 2007 Sep;18(9):1539–47.

For peer review only

24. Bérubé A, Tapp D, Dupéré S, Plaisance A, Bravo G, Downar J, et al. Do Socioeconomic Factors Influence Knowledge, Attitudes, and Representations of End-of-Life Practices? A Cross-Sectional Study. J Palliat Care. 2022 Oct 13;8258597221131658.

25. Morita T, Hirai K, Okazaki Y. Preferences for Palliative Sedation Therapy in the Japanese General Population. J Palliat Med. 2002 Jun;5(3):375–85.

26. Rietjens JA, van der Heide A, Onwuteaka-Philipsen BD, van der Maas PJ, van der Wal G. Preferences of the Dutch general public for a good death and associations with attitudes towards end-of-life decision-making. Palliat Med. 2006 Oct 1;20(7):685–92.

27. Directives anticipées [Internet]. Fédération des médecins suisses; 2022 [cited 2022 Apr 1]. Available from: https://www.fmh.ch/fr/prestations/droit/directives-anticipees.cfm

28. Börsch-Supan A, Brandt M, Hunkler C, Kneip T, Korbmacher J, Malter F, et al. Data Resource Profile: The Survey of Health, Ageing and Retirement in Europe (SHARE). Int J Epidemiol. 2013 Aug 1;42(4):992–1001.

29. Hoffmeyer-Zlotnik JHP, Hoffmeyer-Zlotnik JHP, Wolf C. Advances in Cross-National Comparison: A European Working Book for Demographic and Socio-economic Variables. Springer Science & Business Media; 2003. 432 p.

30. Börsch-Supan A. The Survey of Health, Ageing and Retirement in Europe (SHARE): Release Guides 8.0.0. 2022;

31. Ouellette L, Puro A, Weatherhead J, Shaheen M, Chassee T, Whalen D, et al. Public knowledge and perceptions about cardiopulmonary resuscitation (CPR): Results of a multicenter survey. Am J Emerg Med. 2018 Oct;36(10):1900–1.

32. Colwill M, Somerville C, Lindberg E, Williams C, Bryan J, Welman T. Cardiopulmonary resuscitation on television: are we miseducating the public? Postgrad Med J. 2018 Feb;94(1108):71–5.

33. Ramirez L, Diaz J, Alshami A, Varon DS, Einav S, Surani S, et al. Cardiopulmonary resuscitation in television medical dramas: Results of the TVMD2 study. Am J Emerg Med. 2021 May;43:238–42.

34. Buiting HM, van Delden JJM, Rietjens JAC, Onwuteaka-Philipsen BD, Bilsen J, Fischer S, et al. Forgoing artificial nutrition or hydration in patients nearing death in six European countries. J Pain Symptom Manage. 2007 Sep;34(3):305–14.

35. Rietjens JAC, van der Heide A, Voogt E, Onwuteaka-Philipsen BD, van der Maas PJ, van der Wal G. Striving for quality or length at the end-of-life: attitudes of the Dutch general public. Patient Educ Couns. 2005 Nov 1;59(2):158–63.

For peer review only

For peer review only - http://bmjopen.bmj.com/site/about/guidelines.xhtml

36. Meier C, Vilpert S, Borasio GD, Maurer J, Jox RJ. Perceptions and Knowledge Regarding Medical Situations at the End of Life among Older Adults in Switzerland. J Palliat Med [Internet]. 2022 Jun 29 [cited 2022 Jul 4]; Available from: https://www.liebertpub.com/doi/10.1089/jpm.2022.0057

37. Murphy DJ, Burrows D, Santilli S, Kemp AW, Tenner S, Kreling B, et al. The influence of the probability of survival on patients' preferences regarding cardiopulmonary resuscitation. N Engl J Med. 1994 Feb 24;330(8):545–9.

38. Sharma R, Jayathissa S, Weatherall M. Cardiopulmonary resuscitation knowledge and opinions on end of life decision making of older adults admitted to an acute medical service. N Z Med J. 2016 Jan 8;129(1428):26–36.

39. Raghupathi V, Raghupathi W. The influence of education on health: an empirical assessment of OECD countries for the period 1995–2015. Arch Public Health. 2020 Apr 6;78(1):20.

40. Vilpert S, Borrat-Besson C, Maurer J, Borasio GD. Awareness, approval and completion of advance directives in older adults in Switzerland. Swiss Med Wkly. 2018 Jul 16;148:w14642.

41. Vilpert S, Bolliger E, Borrat-Besson C, Borasio GD, Maurer J. Social, cultural and experiential patterning of attitudes and behaviour towards assisted suicide in Switzerland: evidence from a national population-based study. Swiss Med Wkly [Internet]. 2020 Jul 1 [cited 2021 Jan 4];150(2526). Available from: https://smw.ch/article/doi/smw.2020.20275

42. Mercadante S, Ferrera P, Girelli D, Casuccio A. Patients' and relatives' perceptions about intravenous and subcutaneous hydration. J Pain Symptom Manage. 2005 Oct;30(4):354–8.

43. Cotter PE, Simon M, Quinn C, O'Keeffe ST. Changing attitudes to cardiopulmonary resuscitation in older people: a 15-year follow-up study. Age Ageing. 2009 Mar 1;38(2):200–5.

44. van Gijn MS, Frijns D, van de Glind EMM, C. van Munster B, Hamaker ME. The chance of survival and the functional outcome after in-hospital cardiopulmonary resuscitation in older people: a systematic review. Age Ageing. 2014 Jul 1;43(4):456–63.

45. Piscator E, Hedberg P, Göransson K, Djärv T. Survival after in-hospital cardiac arrest is highly associated with the Age-combined Charlson Co-morbidity Index in a cohort study from a two-site Swedish University hospital. Resuscitation. 2016 Feb 1;99:79–83.

46. Chambaere K, Rietjens JA, Cohen J, Pardon K, Deschepper R, Pasman HRW, et al. Is educational attainment related to end-of-life decision-making? A large post-mortem survey in Belgium. BMC Public Health. 2013 Nov 9;13(1):1055.

For peer review only

For peer review only - http://bmjopen.bmj.com/site/about/guidelines.xhtml

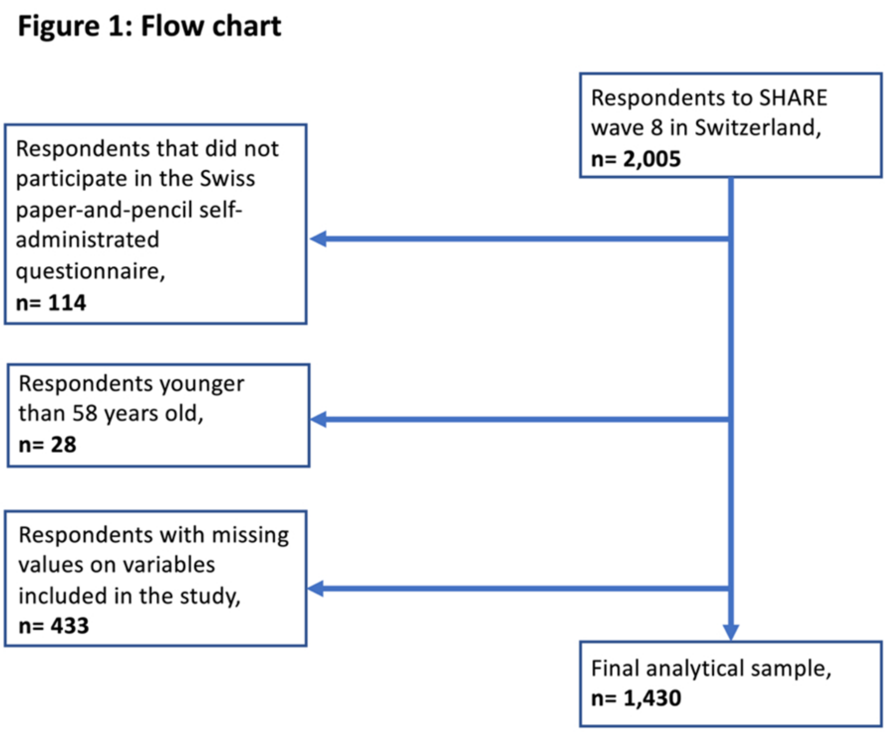

Flow chart

293x242mm (87 x 87 DPI)

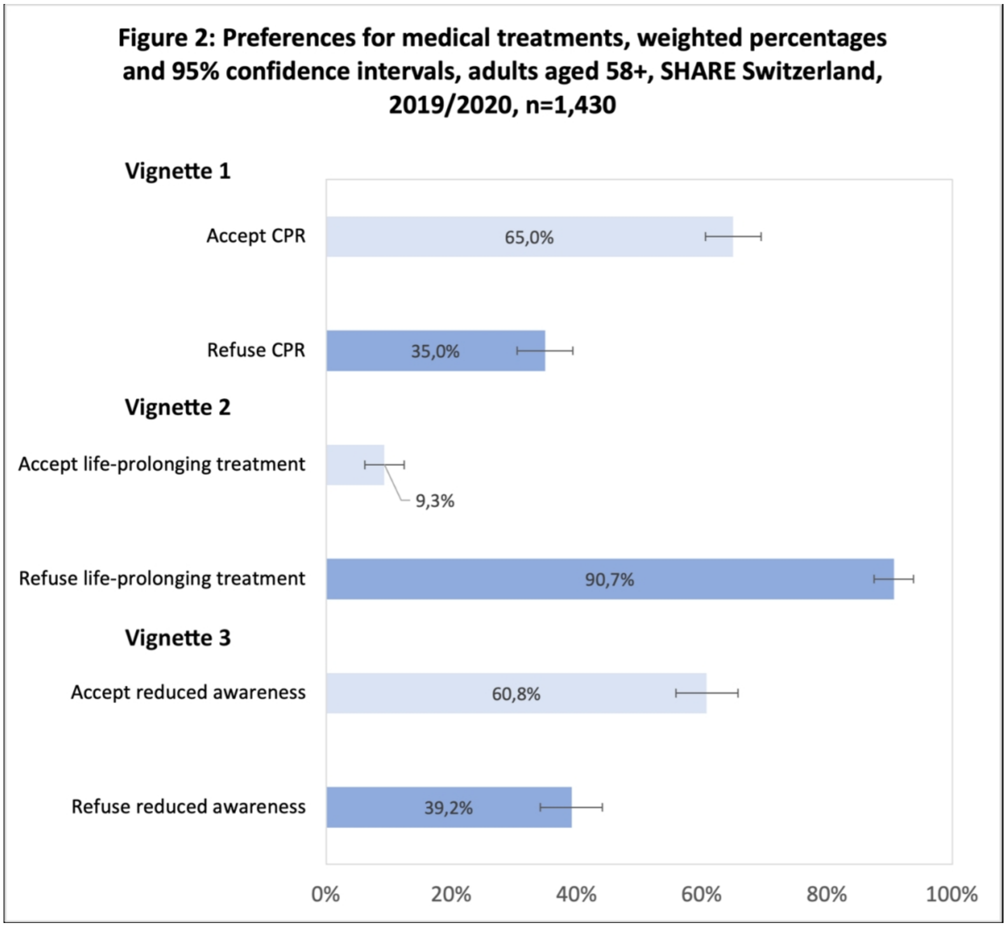

Preferences for medical treatments, weighted percentages and 95% confidence intervals, adults aged 58+, SHARE Switzerland, 2019/2020, n=1,430

404x371mm (87 x 87 DPI)

BMJ Open

Older adults' medical preferences for the end of life: a cross-sectional population-based survey in Switzerland

Journal:	*BMJ Open*
Manuscript ID	bmjopen-2022-071444.R1
Article Type:	Original research
Date Submitted by the Author:	23-May-2023
Complete List of Authors:	Vilpert, Sarah; University of Lausanne, Faculty of Business and Economics (HEC)<br>Meier, Clément; University of Lausanne, Faculty of Business and Economics (HEC); University of Lausanne, Faculty of Biology and Medicine<br>Berche, Jeanne; Unisanté<br>Borasio, Gian Domenico; Centre Hospitalier Universitaire Vaudois, Palliative Care Service<br>Jox, Ralf; Centre Hospitalier Universitaire Vaudois Institut des humanités en médecine; Centre Hospitalier Universitaire Vaudois Service de soins palliatifs et de support<br>Maurer, Jurgen; University of Lausanne Faculty of Business and Economics
**Primary Subject Heading**:	Public health
Secondary Subject Heading:	Communication, Palliative care, Patient-centred medicine
Keywords:	Quality of Life, PUBLIC HEALTH, PAIN MANAGEMENT, INTENSIVE & CRITICAL CARE, Aged, Cardiopulmonary Resuscitation

SCHOLARONE™
Manuscripts

I, the Submitting Author has the right to grant and does grant on behalf of all authors of the Work (as defined in the below author licence), an exclusive licence and/or a non-exclusive licence for contributions from authors who are: i) UK Crown employees; ii) where BMJ has agreed a CC-BY licence shall apply, and/or iii) in accordance with the terms applicable for US Federal Government officers or employees acting as part of their official duties; on a worldwide, perpetual, irrevocable, royalty-free basis to BMJ Publishing Group Ltd ("BMJ") its licensees and where the relevant Journal is co-owned by BMJ to the co-owners of the Journal, to publish the Work in this journal and any other BMJ products and to exploit all rights, as set out in our licence.

The Submitting Author accepts and understands that any supply made under these terms is made by BMJ to the Submitting Author unless you are acting as an employee on behalf of your employer or a postgraduate student of an affiliated institution which is paying any applicable article publishing charge ("APC") for Open Access articles. Where the Submitting Author wishes to make the Work available on an Open Access basis (and intends to pay the relevant APC), the terms of reuse of such Open Access shall be governed by a Creative Commons licence – details of these licences and which Creative Commons licence will apply to this Work are set out in our licence referred to above.

Other than as permitted in any relevant BMJ Author's Self Archiving Policies, I confirm this Work has not been accepted for publication elsewhere, is not being considered for publication elsewhere and does not duplicate material already published. I confirm all authors consent to publication of this Work and authorise the granting of this licence.

For peer review only

Older adults' medical preferences for the end of life: a cross-sectional population-based survey in Switzerland

Abstract:

Objectives: Medical decision-making at the end of life is common and should be as patient-centered as possible. Our study investigates older adults' preferences toward three medical treatments that are frequently included in advance directive forms and their association with social, regional, and health characteristics.

Setting: An observational study using population-based data of wave 8 (2019/20) of the Swiss component of the Survey of Health, Ageing, and Retirement in Europe (SHARE).

Participants: 1,430 adults aged 58 years and older living in Switzerland.

Primary and secondary outcome measures: Three questions on the preferences regarding cardiopulmonary resuscitation (CPR); life-prolonging treatment in case of high risk of permanent mental incapacity; reduced awareness (sedation) to relieve unbearable pain and symptoms. Their associations with individuals' social, regional and health characteristics.

Results: Most older adults expressed a wish to receive CPR (58.6%) and to forgo life-prolonging treatment in case of permanent mental incapacity (90.7%). Most older adults also indicated that they would accept reduced awareness if necessary to receive effective treatment for pain and distressing symptoms (60.8%). Older adults' treatment preferences for CPR and life-prolonging treatment differed according to sex, age, partnership status, linguistic region and health status, while willingness to accept reduced awareness for effective symptom treatment was more similarly distributed across population groups.

Conclusions: Simultaneous preferences for CPR and refusal of life-prolonging treatment might appear to be conflicting treatment goals. Considering individuals' values and motivations can help clarify ambivalent treatment decisions. Structured ACP processes with trained professionals allows for exploring individuals' motivations and values and helps to identify congruent care and treatment goals.

Strengths and limitations of this study

- This study replicates the questions on treatment preferences that are usually part of advance directives.
- This study explores treatment preferences in a national population-based sample of older adults.
- The wording of the questions may not provide clear indications of individuals' actual treatment preferences.

Keywords

Advance directives; life-prolonging treatments; cardiopulmonary resuscitation; sedation; population-based study

For peer review only - http://bmjopen.bmj.com/site/about/guidelines.xhtml

Introduction:

Advances in medical technologies and extended life expectancy have changed the context of dying in today's societies (1) and often result in a need to make complex medical decisions at the end of life (EOL) (2,3). EOL decisions may concern pain or symptom management, possibly but not necessarily with life-shortening effects, withholding or withdrawing life-supporting treatment (e.g. cardiopulmonary resuscitation (CPR), artificial nutrition and hydration, mechanical ventilation, dialysis), and, in countries where it is legal, euthanasia and assisted suicide (4,5). Following the principle of patient autonomy (6), EOL decisions are jointly made by the patients and/or their surrogates and clinicians (7). These decisions ought to be based on considerations of prognosis, benefits and risks of different treatments, and patients' values and care goals among others (8). Patients commonly opt for or against certain treatments based on their perceived benefits or costs in terms of quality of life and prolongation of life, while physicians typically consider the medical appropriateness of the treatments under consideration.

EOL decisions are most often the result of multiple discussions between medical experts, patients, and potentially their families which constitute the basis of a process called advance care planning (ACP), when preparing for a patient health situation that may worsen in the future. The ACP outcomes may be recorded in advance directives (ADs). ACP aims to enable patients to receive medical treatments that are better aligned with their values, care preferences and goals (9). ACP thus contributes to maintaining patients' quality of life throughout their care episodes (10), and eventually achieve what they may have defined as a "good death" for themselves during the ACP process (11,12). ADs record the medical treatments to which a person consents or refuses. ADs can also be used to designate a healthcare proxy to make medical decisions on behalf of that person in the event of a loss of decision-making capacity (13). At the end of life, more than half of the patients lose their decision-making capacity (4). While ACP may not always entirely achieve its primary goals such as a positive impact on patients' quality of life or EOL care consistent with patient preference and goals of care, it often achieves other important secondary objectives such as improving the quality of patient-physician communication and congruence in preferences between patients and their caregivers as well as reducing decisional conflict, increasing preferences for comfort care, and encouraging the documentation of ACP and ADs (14,15). Furthermore, the existence of ADs is associated with more out-of-hospital care, an increased focus on comfort care, and may contribute to reducing overtreatment at the end of life (16).

In Switzerland, ADs have been legally binding since 2013 and individuals can find numerous AD forms published by medical associations, patient organizations or other private initiatives. Most AD forms include information on

For peer review only - http://bmjopen.bmj.com/site/about/guidelines.xhtml

medical and health situations in which the wishes expressed in ADs are to be applied, individuals', treatment preferences, organ donation, and designated healthcare proxy, while some of them also contain a section about personal values, the meaning of life and quality of life and related fears and expectations (17). ADs can be completed by individuals alone, although completion with the assistance of trained professionals or healthcare specialists is generally recommended (18). While many people tend to have the impression that ADs are mainly targeted to older adults and persons with terminal illnesses, AD completion is in fact also recommended for healthy younger adults, due to the broader risks of potential loss of decision-making capacity after, for example, an accident (19).

Understanding treatment preferences commonly indicated in AD forms may be helpful to assess attitudes towards EOL medical decisions in the general population and give insights into how to best initiate discussion on the topic. Preferences regarding specific life-sustaining treatments (20–22), general EOL care goals such as priority for length or quality of life (21,23,24), and palliative sedation therapy (25–29) have been studied in the general population, but mostly in hypothetical contexts of severe physical pain or cognitive decline. To our knowledge, these preferences have not been investigated together in the way in which they might appear in AD forms. We, therefore, consider three specific questions taken from the actual AD form (see Appendix 1) used until 2022 by the Swiss Medical Association FMH, the leading physicians association in Switzerland, which comprises 42,000 members and over 70 medical organisations in the country. In this study, we examine older adults' preferences for CPR, life-prolonging treatment, and reduced awareness (sedation) as measured by the questions from the aforementioned AD form in a large population survey of adults aged 58 years and older living in Switzerland. We also investigate the association between stated treatment preferences and individuals' social, regional, and health characteristics. This information is relevant to understanding how older adults view specific EOL treatments and identifying differences in treatment preferences among the population groups.

For peer review only

Methods:

We used data from the Survey of Health, Ageing and Retirement in Europe (SHARE), which collects internationally comparable multidisciplinary longitudinal microdata on social, economic and health conditions of European aged 50 and over and their partners (30). We used a subsample of the Swiss-SHARE Wave 8 (2019/2020) dataset, which included respondents who participated in the internationally harmonized in-person interview and the national paper-and-pencil self-administered EOL questionnaire distributed at the end of the interview in Switzerland. The EOL questionnaire was developed by the Swiss SHARE team and palliative care

For peer review only - http://bmjopen.bmj.com/site/about/guidelines.xhtml

experts of the Lausanne University Hospital and concerns preferences, knowledge, attitudes, and behaviors toward EOL care planning (31). In Wave 8 in Switzerland, 2,005 respondents participated in the SHARE main interview and 94.3% of them (1,891 individuals) also completed the EOL questionnaire. Since the last sample refreshment in Switzerland was in 2011, only 28 respondents to the Swiss-SHARE Wave 8 survey, which had entered the study as partners, were aged 50-57. We, therefore, discard these respondents from our sample and limit our analyses to respondents aged 58 years and older who had no missing values on any of the items used in our analysis. Our selected final analytical sample comprises 1,430 respondents (Figure1).

(Insert figure 1 here)

Measures

Outcome variables:

We included three questions in our EOL questionnaire, which have been adapted directly from the long version of the AD form that was publicly available for download from the Swiss Medical Association FMH website. The adaptations made for inclusion in our survey mainly consisted of transforming the statements into questions, while keeping the vocabulary and contextual information provided as in the Swiss Medical Association's AD forms.

Question 1. Imagine that you experience a cardiac and/or respiratory arrest. In this situation, you wish:

- to be resuscitated (1=accept CPR).
- not to be resuscitated (0=refuse CPR)

Question 2. Imagine that you are incapacitated following an accident, a stroke, or a heart attack. After initial emergency measures and careful medical assessment, physicians deem it very unlikely that you will regain mental capacity. In this situation, you prefer:

- to forgo all measures which would only serve to prolong your life and suffering (0=refuse life-prolonging treatment)
- that, despite the poor outlook, every medically appropriate measure should be taken ((1=accept life-prolonging treatment).

Question 3. Imagine that you suffer from a disease that causes unbearable pain and symptoms such as fear, restlessness, breathing difficulties, and nausea. In this situation:

For peer review only - http://bmjopen.bmj.com/site/about/guidelines.xhtml

- you wish to receive optimal treatment of pain and other distressing symptoms, and you are prepared to accept the reduced awareness (sedation) that such treatment may induce (1=accept reduced awareness)
- for you, alertness and the ability to communicate are more important than optimal relief of pain and other symptoms (0=refuse reduced awareness).

Independent variables:

We considered social, regional and health individuals' characteristics as independent variables in our study. Social characteristics included information on sex (0=male, 1=female), age group (58-64 years, 65-74 years, 75+ years), education level based on the International Standard Classification of Education (ISCED) of 2017 (32) (basic=ISCED levels 0-1-2, secondary=ISCED levels 3-4, tertiary=ISCED levels 5-6), living with a partner (0=has a partner, 1=has no partner), parenthood (0=no children, 1=at least one child) and self-perceived financial difficulties (Is your household able to make ends meet?: 1=easily, 2=fairly easily, 3=with difficulty). Regional characteristics comprised variables on the living environment (0=urban, 1=rural), language regions (German, French or Italian) and religiosity (0=never pray, 1=pray at least sometimes). Health characteristics involved two variables: self-rated health (1 = poor/fair health, 2 = good health, 3 = very good/excellent health) and self-reported limitation in activities of daily living (ADLs) (0=no limitation, 1=at least one limitation).

Statistical analysis:

We performed proportion estimation for all the variables included in the study. We further used probit regressions to assess the partial associations of preferences for medical treatments with respondents' characteristics. Regression estimates are expressed in terms of average partial effects (APEs). APEs report (in percentage points) how the average conditional outcome probability $P(Y=1 \mid X)$ changes when the given explanatory variable changes from 0 to 1, holding all other independent variables at their observed values. Estimated standard errors were clustered at the household level to account for potential unobserved dependencies between partners' responses. All estimations were conducted using STATA/SE 17.0 software (STATA Corporation, College Station, TX).

Ethical approval

The study was approved by the research ethics committee of the canton of Vaud in March 2014 with number 66/14. In addition, during data collection, respondents gave their consent to participate in the survey on two occasions, when they agreed over the phone to schedule an interview and at the beginning of the in-person interview.

For peer review only - http://bmjopen.bmj.com/site/about/guidelines.xhtml

Patient and Public Involvement

Patients and the public were not directly involved in the design of this study as their contribution would not have been relevant to the research question.

Results:

The social, cultural, and health characteristics of the study population are presented in Table 1. Regarding preferences for medical treatments displayed in Figure 2, 58.6% of respondents preferred to receive CPR, 90.7% chose to forgo life-prolonging treatment in case of high risk of permanent mental incapacity, and 60.8% accepted reduced awareness for optimum treatment of pain and other distressing symptoms.

For peer review only

Table 1: Characteristics of the study sample, adults aged 58+, SHARE Switzerland, 2019/2020, n=1,430		
	Number	Proportions (%)
Gender		
Male	676	47.3
Female	754	52.7
Age groups		
58-64 years	351	24.6
65-74 years	603	42.2
75+ years	476	33.3
Education		
Basic	231	16.2
Secondary	911	63.7
Tertiary	288	20.1
Living with a partner		
Yes	1,071	74.9
No	359	25.1
Parenthood		
No children	221	15.5
At least on child	1,209	84.5
Make ends meet		
Easily	798	55.8
Fairly easily	457	32
With difficulty	175	12.2
Living environment		
Urban	657	45.9
Rural	773	54.1
Switzerland linguistic regions		
German	1,037	72.5
French	345	24.1
Italian	48	3.4
Religiosity		
Never pray	441	30.8
Pray at least sometimes	989	69.2
Self-rated health		
Poor/fair health	250	17.5
Good health	607	42.4
Very good/excellent health	573	40.1
Self-reported limitations in activities of daily living (ADLs)		
No limitation	1,343	93.9
At least one limitation	87	6.1

(Insert Figure 2 here)

For peer review only - http://bmjopen.bmj.com/site/about/guidelines.xhtml

Table 2 shows the results from the multivariable probit regressions of preferences for the three different medical treatments on respondents' characteristics. Concerning preferences for CPR, being a woman decreases the probability of wanting to be resuscitated by 11.2 percentage points compared to being a man (APE: -11.2, $p < 0.001$). In addition, living without a partner (APE: -8.2; $p < 0.05$) and being older (APE 65-74: -10.1, $p < 0.01$; APE 75+: -20.4, $p < 0.001$) were also each associated with a lower likelihood of wanting to be resuscitated. In contrast, having a tertiary level of education (APE: 10.3, $p < 0.05$), being from the Italian-speaking part of Switzerland (APE: 24.8, $p < 0.001$), praying at least sometimes (APE: 5.6, $p < 0.01$), and self-rating one's health as good (APE: 9.4, $p < 0.05$) or very good/excellent (APE: 12.0, $p < 0.01$) were positively associated with preferences for CPR.

Regarding general preferences for life-prolonging treatment, being a woman (APE: -4.3, $p < 0.01$), being 65- to 74 years old (APE: -3.8, $p < 0.05$), and having at least one limitation in activities of daily living (APE: -4.8, $p < 0.05$) were all negatively associated with preference for life-prolonging treatment in case of high risk of permanent mental incapacity. On the other side, living without a partner (APE: 4.3, $p < 0.01$) and being from the Italian-speaking part of Switzerland (APE: 17.7, $p < 0.01$) were associated with a higher chance of wanting life-prolonging treatment.

Concerning acceptance of reduced awareness, having a secondary level of education (APE: 8.9, $p < 0.01$) was associated with a higher likelihood of accepting reduced awareness for optimum pain treatment and other distressing symptoms. At the same time, respondents who said they pray at least sometimes (APE: -6.2, $p < 0.01$) were less likely to accept reduced awareness in order to receive optimal treatment of pain and other distressing symptoms.

For peer review only - http://bmjopen.bmj.com/site/about/guidelines.xhtml

**Table 2: Partial associations of preferences for medical treatments with respondents' social, cultural, and health characteristics, adults aged 58+, SHARE Switzerland, 2019/2020, n=1,430**			
	Preference for CPR APE (95% CI)	Preference for life-prolonging treatment APE (95% CI)	Preference for reduced awareness APE (95% CI)
Gender (ref: male)			
Female	-11.2*** (-16.2,-6.3)	-4.3** (-7.3,-1.4)	2.3 (-2.9,7.4)
Age groups (ref: 58-64 years)			
65-74 years	-10.1** (-16.3,-4.0)	-3.8* (-7.6,-0.1)	2.2 (-4.2,8.5)
75+ years	-20.4*** (-27.3,-13.5)	-4.0 (-8.0,0.0)	-5.1 (-12.4,2.2)
Education (ref: basic)			
Secondary	1.2 (-6.0,8.4)	-4.1 (-8.8,0.7)	8.9* (1.4,16.3)
Tertiary	10.3* (1.5,19.1)	-4.1 (-9.5,1.4)	7.6 (-1.8,16.9)
Living with a partner (ref: yes)			
No	-8.2* (-14.4,-1.9)	4.3* (0.5,8.1)	2.2 (-4.1,8.5)
Parenthood (ref: no children)			
At least on child	0.6 (-6.5,7.7)	-0.6 (-4.5,3.2)	3.3 (-3.9,10.5)
Ease of making ends meet (ref: easy)			
Fairly easily	1.6 (-4.2,7.4)	1.1 (-2.0,4.3)	-2.4 (-8.4,3.7)
With difficulty	2.2 (-6.0,10.3)	2.9 (-2.2,8.1)	-0.0 (-8.7,8.6)
Living environment (ref: urban)			
Rural	1.4 (-3.8,6.6)	0.3 (-2.5,3.0)	-0.0 (-5.4,5.3)
Switzerland linguistic regions (ref: German)			
French	-6.1 (-12.3,0.0)	2.3 (-1.1,5.7)	2.6 (-3.5,8.7)
Italian	24.8*** (15.2,34.3)	17.7* (4.0,31.4)	-7.5 (-23.5,8.5)
Religiosity (ref: never pray)			
Pray at least sometimes	5.6* (0.1,11.1)	0.5 (-2.5,3.5)	-6.2* (-11.8,-0.5)
Self-rated health (ref: poor/fair health)			
Good health	9.4* (1.8,16.9)	-2.4 (-6.6,1.8)	2.6 (-4.8,10.0)
Very good/excellent health	12.0** (4.0,20.0)	-1.9 (-6.3,2.5)	-3.3 (-11.4,4.7)
Self-reported limitations in activities of daily living (ADLs) (ref: no limitation)			
At least one limitation	-1.7 (-12.7,9.4)	-4.8* (-8.6,-0.9)	0.9 (-10.4,12.3)
Observations	1,430	1,430	1,430

*CPR=Cardiopulmonary resuscitation. Average partial effects (APEs) based on probit regression models. All probabilities are multiplied by 100 to express APEs in percentage points. Asterisks indicate levels of significance: ***<0.1%, ** 1%, *5%. Interpretation of APEs: Being a woman decreases the probability of wanting to be resuscitated by 11.2 percentage points compared to being a man.*

For peer review only - http://bmjopen.bmj.com/site/about/guidelines.xhtml
For peer review only

Discussion

Our study is the first national population-based study in Switzerland that investigates older adults' preferences toward specific medical treatments that are commonly elicited in official AD forms. Most respondents wished to receive CPR in case of a cardiac/respiratory arrest, to forgo life-prolonging treatment in case of high risk of permanent mental incapacity, and to reduce their awareness for optimal treatment of pain and other distressing symptoms. Additionally, significant social, regional, and health-related differences in medical treatment preferences were found for CPR and life-sustaining treatments, while preferences for reduced awareness were more uniformly distributed across different population groups.

Preference for cardiopulmonary resuscitation

A majority of individuals reported wanting to receive CPR in the event of cardiac and/or respiratory arrest. In most population-based studies investigating preferences for CPR, the question is asked for a given health condition (e.g., terminal cancer, advanced dementia), and individuals largely refuse CPR (20,21,25). There are several reasons that could explain this difference between our study and previous ones. In our study, the context of the CPR is not specified, and respondents may be referring to their current health status, which is generally rather good (84.8% declared a good, very good, or excellent health) with the preference for CPR being potentially influenced by the prospect of significant remaining longevity (24). Alternatively, this difference could also be explained by an overestimation of CPR survival rate (33), which may lead individuals to choose CPR (34). Some individuals may decide against CPR once they have been informed of its procedure, risks and benefits in different contexts (35).

Regarding social differences in CPR preferences in our study, women reported lower preferences for CPR than men. This finding, which has been shown in other studies (21,25), could be explained by a higher valuation of quality of life over length of life among women compared to men (36) which may lead them to refrain from treatments (37). Moreover, women are more likely to have direct experience with caregiving (38) that may make them more aware of the benefits and limitations of certain medical treatments (33) and the potential burden that these treatments may imply for patients and their relatives (24). Respondents living without a partner were also less likely to opt for CPR than respondents living with a partner. Since respondents living without a partner also seem to be better informed about the limited success of CPR, as shown in a previous study by the authors based on the same Swiss subsample of SHARE Wave 8 (33), they may prefer to refuse CPR (39).

Conversely, respondents with a tertiary level of education were more likely to report a preference for CPR. Our previous study (33) showed that older adults with higher levels of education were not more knowledgeable about

For peer review only - http://bmjopen.bmj.com/site/about/guidelines.xhtml

the (relatively low) chances of success of CPR and may, therefore, not be more likely to forgo CPR than persons with less education (33). A study of hospitalized patients aged 65 and over in New Zealand reported that more educated patients were more likely to prefer CPR in their current state of health (40). One potential reason could be that individuals with higher education believe to have better health prospects (41) which could induce them to opt for CPR. One study showed that older adults who are optimistic about their own life expectancy are more likely to want to continue all medical treatment in a situation of serious illness even if there are very low chances of survival (24). Acceptance of CPR was also higher in the Italian linguistic region and among more religious people, who report to pray at least sometimes. These findings may highlight well-documented cultural differences in Switzerland, with respondents from the Italian-speaking part (42,43) and more religious persons being more likely to be influenced by religious considerations and corresponding beliefs in the sanctity of life (37,43,44) that tend to encourage life-prolonging treatments (11).

Finally, age and self-rated health status, which are two parameters of medical prognoses, were also strongly associated with CPR preferences. Compared to adults aged 58-64, older respondents were less likely to state that they would prefer to receive CPR, with this negative association increasing in age. These results are consistent with other studies showing that older people are more likely to refuse CPR (21,25,45). Older adults might realize that age decreases their chances of surviving a CPR (46) and, therefore, be more likely to reject this treatment. The same reasoning may also apply to people in poorer health (47).

Preference for life-prolonging treatment

Over 90% of our respondents indicated a preference to forgo life-prolonging treatment if they were to become incapacitated following an accident, a stroke, or a heart attack and were very unlikely to regain mental capacity. Similar results were found in studies of preferences to continue or stop treatment in the presence of serious physical or mental illness in the general population (21,24). Interestingly, the population groups more likely to forgo life-prolonging treatments are comparable to those who refused CPR, that is, women, older persons, and those in poorer health (1+ ADLs). These findings are in line with a study of the general Dutch population that showed that women, older people, and people with a history of serious illness tend to prioritize quality of life over quantity (37). In contrast, Swiss participants from the Italian-speaking region who were more likely to prefer CPR were also more likely to prefer life-prolonging treatment. A surprising result for which we have no explanation is the preference for life-sustaining treatments among people living without a partner, especially since many of them simultaneously indicated to refuse CPR.

For peer review only - http://bmjopen.bmj.com/site/about/guidelines.xhtml

Preference for reduced awareness

In our study, 60.8% of our respondents accepted reduced awareness in a situation where they would suffer from a disease that causes unbearable pain and symptoms such as fear, restlessness, breathing difficulties, and nausea, while for the remaining respondents (39.2%), awareness and the ability to communicate was more important than optimal relief of pain and other symptoms. Other studies of individuals' preferences for different types of palliative sedation (e.g. intermittent, mild, continuous deep sedation), in different situations and for different durations have produced mixed results, which are not directly comparable to ours (25–29). Interestingly, although adequate pain and symptoms management is one of the most important features of a good death (28), preservation of consciousness also plays a central role at the end of life for many people (11,25,27). In a Dutch study, 61% of adults aged 20+ reported being conscious until death as important. Those individuals were less likely to accept terminal sedation (67%) and the use of high dosages of morphine (67%) (28). These findings highlight the clinical importance to clearly discuss the possible interaction and trade-offs between symptom alleviation and decreased alertness with patients.

Only education and religiosity were associated with individuals' preferences for or against reduced awareness as a side effect of optimal symptom treatment. People with secondary education are more likely to accept reduced awareness, which is consistent with a Belgian study that showed that patients with higher levels of education are more likely to emphasize pain and symptom relief at the end of life (48). Furthermore, more religious respondents were less likely to accept reduced awareness. Another study pointed that the proportion of individuals rejecting gradual and rapid sedation was higher among more religious people (29). These associations may be linked to the belief that symptom treatment that reduces awareness also shortens life (49), especially since religiosity is commonly associated with life-prolonging treatment preferences among older adults (21,24,37).

Potential discrepancy in treatment preferences

Our results may at first seem contradictory in that most respondents indicated a preference against life-prolonging treatments but at the same time showed a preference for CPR, which has a relatively low chance of survival. This type of ambivalence has already been noted in a study that showed that about one-third of the Dutch adult population could not make a clear choice between quality and quantity of life (37), which could be compared in our study with the refusal of EOL treatments (quality of life) and the simultaneous preference for resuscitation (quantity of life). Another study also pointed to inconsistencies between Dutch older adults' general EOL goals

For peer review only - http://bmjopen.bmj.com/site/about/guidelines.xhtml

and preferences for specific life-sustaining treatments (20). Their authors explained these results by the lack of knowledge of the risks and side effects of these treatments in the population.

The apparent contradiction between preferences for CPR and life-prolonging treatments in our study may be related to the framing of our questions. Our CPR question provides no information about the context in which the CPR is to be conducted such as whether the CPR would take place in- or out-hospital, and individual's health status is also not specified in this question. By contrast, the question on life-prolonging treatment refers to a situation of very bad health which may explain why the overwhelming majority of respondents reported a preference to refuse life-prolonging treatment in this context. Both questions were directly drawn from the Swiss Medical Association's AD form, which was in use at the time of our survey, but has been revised since. The long version of this form contains supplementary contextual information on individuals' motivations, health history and social situation, which seems helpful in translating patients' values and preferences into medically actionable information. Nevertheless, this supplementary information may not completely prevent potential conflicting information about individuals' treatment preferences in case of patients' loss of decision capacity, especially if this information may stand in contrast to individuals' treatment wishes as indicated by the check box questions. A broader approach to ACP that can be translated into more nuanced ADs is clearly desirable and increasingly implemented in practice (10).

Limitations

One limitation of our study is that our question wording may not be ideal for eliciting people's treatment preferences even if the questions were highly relevant to Swiss EOL policy at the time of our survey due to their close correspondence with a widely known AD form in Switzerland. Another limitation is that people in very poor health or living in a nursing home may be underrepresented in our study, despite the considerable efforts of SHARE to interview also sicker people and follow respondents into institutions such as nursing homes. On the other hand, the EOL questionnaire's cooperation rate was high (94.3%), and an analysis of the profile of non-respondents based on individuals' characteristics used in our regression models indicated no obvious underrepresentation of certain population groups.

For peer review only - http://bmjopen.bmj.com/site/about/guidelines.xhtml
For peer review only

Conclusion

Our study investigates preferences for CPR, life-prolonging treatments, and reduced awareness in the general population of adults aged 58 years and older living in Switzerland based on three statements included in the FMH Swiss Medical Association's AD form. Most older adults wished to receive CPR in case of a cardiac/respiratory arrest, forgo life-prolonging treatment in case of high risk of permanent mental incapacity, and reduce their awareness for optimal treatment of pain and other distressing symptoms. Our study also revealed that individuals' preferences appear to be influenced by characteristics that affect medical prognoses, such as advanced age and poor health. Moreover, women were less likely to report a preference for CPR and life-prolonging treatments than men. Furthermore, the apparent contradiction in our findings between individuals' willingness to receive CPR and their wishes to forgo life-prolonging treatment may reveal knowledge gaps regarding the success chances and side effects of certain treatments and also highlights the potential ambivalence between individuals' EOL care goals and the choice they make for specific EOL treatments. This inherent complexity of making advance decisions in the context of ADs could benefit from further contextual information and elicitation of individuals' values through processes of ACP, which facilitate a clearer understanding of individuals' values, motivations and fears concerning EOL care that can help to better translate ADs and related information into medical decisions at the EOL.

Contributorship statement

SV designed the study. CM and JB conducted the statistical analysis with the support of SV. SV, CM and JM drafted the manuscript. All authors discussed the interpretation of findings and provided critical revision of the manuscript for important intellectual content.

Competing interests

The authors declare that they have no competing interests.

Funding

This work was supported by the Swiss National Science Foundation (SNSF), grant number 10001C_188836.

This paper uses data from Börsch-Supan, A. (2021). Survey of Health, Ageing and Retirement in Europe (SHARE) Wave 8. Release version: 1.0.0. SHARE-ERIC. Data set. DOI: 10.6103/SHARE.w8.100. The SHARE data collection has been funded by the European Commission, DG RTD through FP5 (QLK6-CT-2001-00360), FP6 (SHARE-I3: RII-CT-2006-062193, COMPARE: CIT5-CT-2005-028857, SHARELIFE: CIT4-CT-2006-028812),

For peer review only - http://bmjopen.bmj.com/site/about/guidelines.xhtml

FP7 (SHARE-PREP: GA N°211909, SHARE-LEAP: GA N°227822, SHARE M4: GAN°261982, DASISH: GA N°283646) and Horizon 2020 (SHARE-DEV3: GA N°676536, SHARE-COHESION: GA N°870628, SERISS: GA N°654221, SSHOC: GA N°823782) and by DG Employment, Social Affairs & Inclusion through VS 2015/0195, VS 2016/0135, VS 2018/0285, VS 2019/0332, and VS 2020/0313. Additional funding from the German Ministry of Education and Research, the Max Planck Society for the Advancement of Science, the U.S. National Institute on Aging (U01_AG09740-13S2, P01_AG005842, P01_AG08291, P30_AG12815, R21_AG025169, Y1-AG-4553-01, IAG_BSR06-11, OGHA_04-064, HHSN271201300071C, RAG052527A) and from various national funding sources is gratefully acknowledged (see https://share-eric.eu/).

Data sharing statement

SHARE data are publicly available for scientific purposes only through registration on the data distribution platform.

Ethics Approval

The study was approved by the research ethics committee of the canton of Vaud in March 2014 with number 66/14.

For peer review only

References

1. Etkind SN, Bone AE, Gomes B, Lovell N, Evans CJ, Higginson IJ, et al. How many people will need palliative care in 2040? Past trends, future projections and implications for services. BMC Med. 2017 May 18;15(1):102.

2. Bosshard G, Zellweger U, Bopp M, Schmid M, Hurst SA, Puhan MA, et al. Medical End-of-Life Practices in Switzerland: A Comparison of 2001 and 2013. JAMA Intern Med. 2016 Apr;176(4):555–6.

3. Becker C, Künzli N, Perrig S, Beck K, Vincent A, Widmer M, et al. Code status discussions in medical inpatients: results of a survey of patients and physicians. Swiss Med Wkly. 2020 Mar 23;150(1314).

4. van der Heide A, Deliens L, Faisst K, Nilstun T, Norup M, Paci E, et al. End-of-life decision-making in six European countries: descriptive study. The Lancet. 2003 Aug 2;362(9381):345–50.

5. Löfmark R, Nilstun T, Cartwright C, Fischer S, van der Heide A, Mortier F, et al. Physicians' experiences with end-of-life decision-making: Survey in 6 European countries and Australia. BMC Med. 2008 Feb 12;6(1):4.

6. Principles of Biomedical Ethics - Paperback - Tom L. Beauchamp; James F. Childress - Oxford University Press [Internet]. [cited 2022 Nov 16]. Available from: //global.oup.com/ushe/product/principles-of-biomedical-ethics-9780190640873

7. Rosca A, Krones T, Biller-Andorno N. Shared decision making: patients have a right to be informed about possible treatment options and their risks and benefits. Swiss Med Wkly. 2020 Jul 6;150(2728):w20268–w20268.

8. Mehta RS, Anderson WG, Hunt S, Chaitin EK, Arnold RM. Chapter 22 - Withholding and Withdrawing Life-Sustaining Therapies. In: Emanuel LL, Librach SL, editors. Palliative Care (Second Edition) [Internet]. Saint Louis: W.B. Saunders; 2011 [cited 2023 Apr 25]. p. 300–18. Available from: https://www.sciencedirect.com/science/article/pii/B9781437716191000226

9. Sudore RL, Lum HD, You JJ, Hanson LC, Meier DE, Pantilat SZ, et al. Defining Advance Care Planning for Adults: A Consensus Definition From a Multidisciplinary Delphi Panel. J Pain Symptom Manage. 2017 May;53(5):821-832.e1.

10. Rietjens JAC, Sudore RL, Connolly M, van Delden JJ, Drickamer MA, Droger M, et al. Definition and recommendations for advance care planning: an international consensus supported by the European Association for Palliative Care. Lancet Oncol. 2017 Sep;18(9):e543–51.

11. Borrat-Besson C, Vilpert S, Borasio GD, Maurer J. Views on a "Good Death": End-of-Life Preferences

For peer review only

For peer review only - http://bmjopen.bmj.com/site/about/guidelines.xhtml

and Their Association With Socio-Demographic Characteristics in a Representative Sample of Older Adults in Switzerland. OMEGA - J Death Dying. 2020 Jul 29;

12. Krikorian A, Maldonado C, Pastrana T. Patient's Perspectives on the Notion of a Good Death: A Systematic Review of the Literature. J Pain Symptom Manage. 2020 Jan;59(1):152–64.

13. Pautex S, Herrmann F, Zulian G. Role of advance directives in palliative care units: a prospective study. Palliat Med. 2008 Oct 1;22(7):835–41.

14. Malhotra C, Shafiq M, Batcagan-Abueg APM. What is the evidence for efficacy of advance care planning in improving patient outcomes? A systematic review of randomised controlled trials. BMJ Open. 2022 Jul 1;12(7):e060201.

15. Jimenez G, Tan WS, Virk AK, Low CK, Car J, Ho AHY. Overview of Systematic Reviews of Advance Care Planning: Summary of Evidence and Global Lessons. J Pain Symptom Manage. 2018 Sep;56(3):436-459.e25.

16. Brinkman-Stoppelenburg A, Rietjens JA, van der Heide A. The effects of advance care planning on end-of-life care: A systematic review. Palliat Med. 2014 Sep 1;28(8):1000–25.

17. Rüegger H. Patientenverfügungen in der deutschsprachigen Schweiz, Institut Neumünster, CURAVIVA. 2011;45.

18. Medical-ethical Guidelines [Internet]. Medical-ethical Guidelines. [cited 2023 Apr 24]. Available from: https://www.samw.ch

19. Otte I, Jung C, Elger B, Bally K. Advance directives and the impact of timing. A qualitative study with Swiss general practitioners. Swiss Med Wkly [Internet]. 2014 Sep 21 [cited 2022 Apr 1];(39). Available from: https://smw.ch/article/doi/smw.2014.14035

20. Evans N, Pasman HR, Deeg D, Onwuteaka-Philipsen B, EURO IMPACT. How do general end-of-life treatment goals and values relate to specific treatment preferences? a population-based study. Palliat Med. 2014 Dec;28(10):1206–12.

21. van Wijmen MPS, Pasman HRW, Widdershoven GAM, Onwuteaka-Philipsen BD. Continuing or forgoing treatment at the end of life? Preferences of the general public and people with an advance directive. J Med Ethics. 2015 Aug;41(8):599–606.

22. Hamano J, Hanari K, Tamiya N. End-of-life care preferences of the general public and recommendations of healthcare providers: a nationwide survey in Japan. BMC Palliat Care. 2020 Mar 24;19(1):38.

For peer review only

For peer review only - http://bmjopen.bmj.com/site/about/guidelines.xhtml

23. Chung RYN, Wong ELY, Kiang N, Chau PYK, Lau JYC, Wong SYS, et al. Knowledge, Attitudes, and Preferences of Advance Decisions, End-of-Life Care, and Place of Care and Death in Hong Kong. A Population-Based Telephone Survey of 1067 Adults. J Am Med Dir Assoc. 2017 Apr 1;18(4):367.e19-367.e27.

24. Carr D, Moorman SM. End-of-Life Treatment Preferences Among Older Adults: An Assessment of Psychosocial Influences. Sociol Forum Randolph NJ. 2009 Dec 1;24(4):754–78.

25. Sanjo M, Miyashita M, Morita T, Hirai K, Kawa M, Akechi T, et al. Preferences regarding end-of-life cancer care and associations with good-death concepts: a population-based survey in Japan. Ann Oncol Off J Eur Soc Med Oncol. 2007 Sep;18(9):1539–47.

26. Bérubé A, Tapp D, Dupéré S, Plaisance A, Bravo G, Downar J, et al. Do Socioeconomic Factors Influence Knowledge, Attitudes, and Representations of End-of-Life Practices? A Cross-Sectional Study. J Palliat Care. 2022 Oct 13;8258597221131658.

27. Morita T, Hirai K, Okazaki Y. Preferences for Palliative Sedation Therapy in the Japanese General Population. J Palliat Med. 2002 Jun;5(3):375–85.

28. Rietjens JA, van der Heide A, Onwuteaka-Philipsen BD, van der Maas PJ, van der Wal G. Preferences of the Dutch general public for a good death and associations with attitudes towards end-of-life decision-making. Palliat Med. 2006 Oct 1;20(7):685–92.

29. Takla A, Savulescu J, Kappes A, Wilkinson DJC. British laypeople's attitudes towards gradual sedation, sedation to unconsciousness and euthanasia at the end of life. PLOS ONE. 2021 Mar 26;16(3):e0247193.

30. Börsch-Supan A, Brandt M, Hunkler C, Kneip T, Korbmacher J, Malter F, et al. Data Resource Profile: The Survey of Health, Ageing and Retirement in Europe (SHARE). Int J Epidemiol. 2013 Aug 1;42(4):992–1001.

31. Wave 8 [Internet]. [cited 2023 Apr 24]. Available from: https://share-eric.eu/data/data-documentation/questionnaires/wave-8

32. Hoffmeyer-Zlotnik JHP, Hoffmeyer-Zlotnik JHP, Wolf C. Advances in Cross-National Comparison: A European Working Book for Demographic and Socio-economic Variables. Springer Science & Business Media; 2003. 432 p.

33. Meier C, Vilpert S, Borasio GD, Maurer J, Jox RJ. Perceptions and Knowledge Regarding Medical Situations at the End of Life among Older Adults in Switzerland. J Palliat Med [Internet]. 2022 Jun 29 [cited 2022 Jul 4]; Available from: https://www.liebertpub.com/doi/10.1089/jpm.2022.0057

For peer review only

34. Ouellette L, Puro A, Weatherhead J, Shaheen M, Chassee T, Whalen D, et al. Public knowledge and perceptions about cardiopulmonary resuscitation (CPR): Results of a multicenter survey. Am J Emerg Med. 2018 Oct;36(10):1900–1.

35. Kerridge IH, Pearson SA, Rolfe IE, Lowe M, McPhee JR. Impact of written information on knowledge and preferences for cardiopulmonary resuscitation. Med J Aust. 1999;171(5):239–42.

36. Buiting HM, van Delden JJM, Rietjens JAC, Onwuteaka-Philipsen BD, Bilsen J, Fischer S, et al. Forgoing artificial nutrition or hydration in patients nearing death in six European countries. J Pain Symptom Manage. 2007 Sep;34(3):305–14.

37. Rietjens JAC, van der Heide A, Voogt E, Onwuteaka-Philipsen BD, van der Maas PJ, van der Wal G. Striving for quality or length at the end-of-life: attitudes of the Dutch general public. Patient Educ Couns. 2005 Nov 1;59(2):158–63.

38. Haberkern K, Schmid T, Szydlik M. Gender differences in intergenerational care in European welfare states. Ageing Soc. 2015 Feb;35(2):298–320.

39. Murphy DJ, Burrows D, Santilli S, Kemp AW, Tenner S, Kreling B, et al. The influence of the probability of survival on patients' preferences regarding cardiopulmonary resuscitation. N Engl J Med. 1994 Feb 24;330(8):545–9.

40. Sharma R, Jayathissa S, Weatherall M. Cardiopulmonary resuscitation knowledge and opinions on end of life decision making of older adults admitted to an acute medical service. N Z Med J. 2016 Jan 8;129(1428):26–36.

41. Raghupathi V, Raghupathi W. The influence of education on health: an empirical assessment of OECD countries for the period 1995–2015. Arch Public Health. 2020 Apr 6;78(1):20.

42. Vilpert S, Borrat-Besson C, Maurer J, Borasio GD. Awareness, approval and completion of advance directives in older adults in Switzerland. Swiss Med Wkly. 2018 Jul 16;148:w14642.

43. Vilpert S, Bolliger E, Borrat-Besson C, Borasio GD, Maurer J. Social, cultural and experiential patterning of attitudes and behaviour towards assisted suicide in Switzerland: evidence from a national population-based study. Swiss Med Wkly [Internet]. 2020 Jul 1 [cited 2021 Jan 4];150(2526). Available from: https://smw.ch/article/doi/smw.2020.20275

44. Mercadante S, Ferrera P, Girelli D, Casuccio A. Patients' and relatives' perceptions about intravenous and subcutaneous hydration. J Pain Symptom Manage. 2005 Oct;30(4):354–8.

45. Cotter PE, Simon M, Quinn C, O'Keeffe ST. Changing attitudes to cardiopulmonary resuscitation in

For peer review only

For peer review only - http://bmjopen.bmj.com/site/about/guidelines.xhtml

older people: a 15-year follow-up study. Age Ageing. 2009 Mar 1;38(2):200–5.

46. van Gijn MS, Frijns D, van de Glind EMM, C. van Munster B, Hamaker ME. The chance of survival and the functional outcome after in-hospital cardiopulmonary resuscitation in older people: a systematic review. Age Ageing. 2014 Jul 1;43(4):456–63.

47. Piscator E, Hedberg P, Göransson K, Djärv T. Survival after in-hospital cardiac arrest is highly associated with the Age-combined Charlson Co-morbidity Index in a cohort study from a two-site Swedish University hospital. Resuscitation. 2016 Feb 1;99:79–83.

48. Chambaere K, Rietjens JA, Cohen J, Pardon K, Deschepper R, Pasman HRW, et al. Is educational attainment related to end-of-life decision-making? A large post-mortem survey in Belgium. BMC Public Health. 2013 Nov 9;13(1):1055.

49. Grant M, Ugalde A, Mancuso SG, Vafiadis P, Philip J. Morphine use in cancer care: A survey of attitudes and perceptions in general practice patients. Aust Fam Physician. 2017 Oct;46(10):775–80.

Figure 1: Flow chart for study respondents

Figure 2: Preferences for medical treatments, percentages and 95% confidence intervals, adults aged 58+, SHARE Switzerland, 2019/2010, n=1,430

For peer review only

For peer review only - http://bmjopen.bmj.com/site/about/guidelines.xhtml

Figure 1: Flow chart

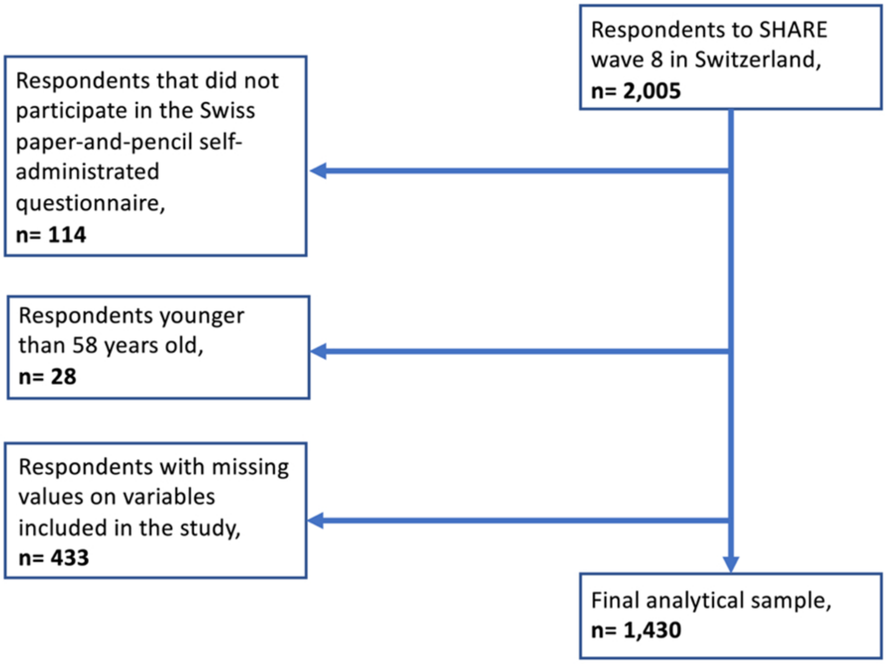

293x242mm (87 x 87 DPI)

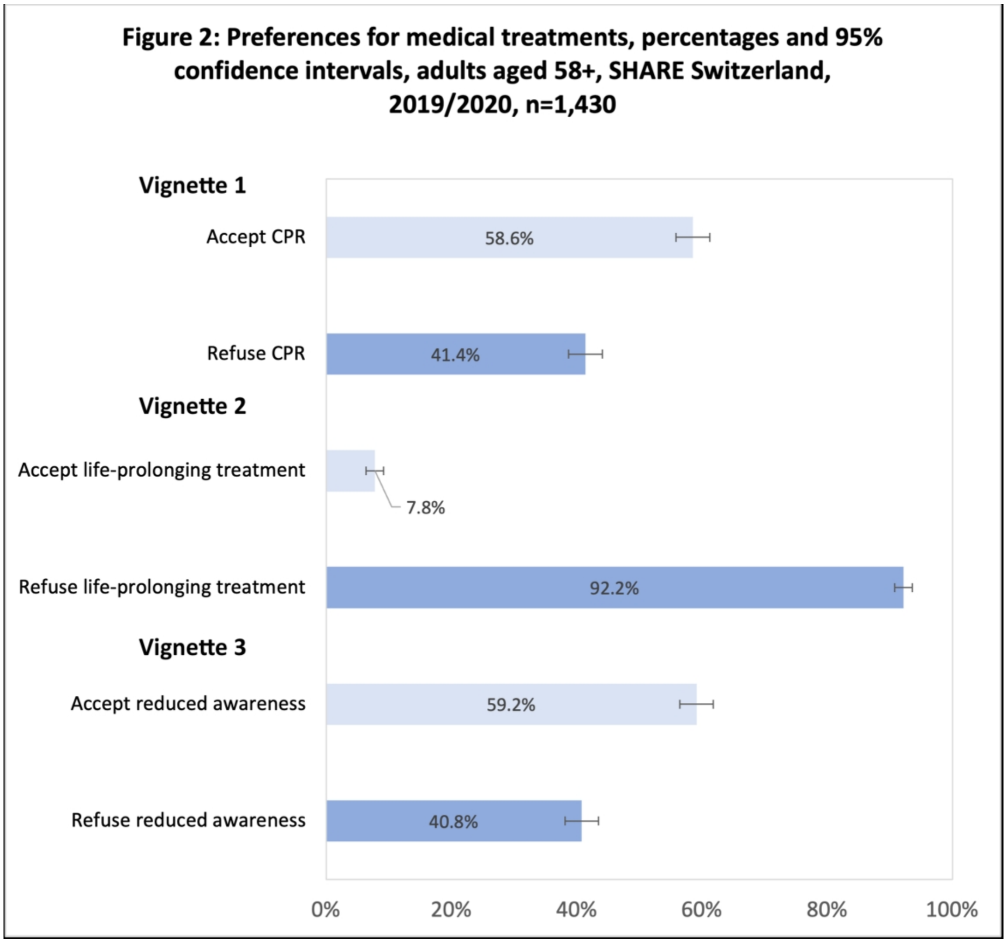

179x167mm (300 x 300 DPI)

FMH

SAMW ASSM
Schweizerische Akademie der Medizinischen Wissenschaften
Académie Suisse des Sciences Médicales
Accademia Svizzera delle Scienze Mediche
Swiss Academy of Medical Sciences

Advance directive | Detailed version

Drawn up by

Name, first name ____________________

Date of birth ____________________

Place of residence ____________________

1. This advance directive is applicable in the following situations

I am drawing up this advance directive after careful reflection in case, at some point, I am no longer able to express my wishes as a result of illness or an accident. The advance directive is to be applicable:

☐ in all situations where I am incapacitated and decisions on medical treatment have to be taken – i.e. in the case of unexpected acute events such as a heart attack, stroke or accident, but also in advanced stages of chronic disease;

or

☐ ____________________

____

2. My motivation and personal values

The following description of my motivation and personal values is a product of careful reflection and is intended to guide the health care team in the event of any difficulties of interpretation.

The following specific situation has prompted me to draw up this advance directive:

☐ (description, if appropriate) ____________________

____

☐ No specific situation; this is a precautionary measure in case I should become incapacitated.

What I particularly wish to achieve with this advance directive is…

…that all possible medical measures are taken to keep me alive. My suffering should be relieved as far as possible, but I accept that certain burdens may be associated with the preservation of my life.<br>☐ This reflects my views more closely.	or	…that medical treatment should aim above all to relieve suffering. Prolonging life at all costs is not the priority for me. I accept that my life may be shortened if certain medical treatments are withheld.<br>☐ This reflects my views more closely.

For peer review only

Describe in your own words your motivation for drawing up an advance directive:

My current situation:

My attitude to life:

My experience, attitude and fears concerning disease, dying and death:

What I understand by ‘quality of life’; what impairments/losses of independence I would find it difficult to accept:

Personal and/or religious convictions by which I am guided:

☐ As a member of the following religion, I wish to receive pastoral care:

☐ I do not wish to receive pastoral care.

For peer review only

3. Statements concerning treatment goals and specific medical measures

In drawing up this advance directive, I have received advice from

☐ My GP, Dr ______________________

☐ or ______________________

☐ I do not wish to give any instructions concerning specific medical measures, but I would ask the health care team to act as far as possible in accordance with my wishes (see Section 2: 'My motivation and personal values').

☐ I wish to give specific instructions for the following situations (cf. choices I - IV).

I Unexpected acute event (e.g. accident, stroke, heart attack)

If I am incapacitated as a result of an unexpected acute event and – after initial emergency measures and careful medical assessment – it is deemed impossible or unlikely that I will regain capacity,

☐ I wish to forgo all measures which would serve merely to prolong life and suffering.

☐ I wish that, despite the poor outlook, every medically appropriate measure should be taken.

II Treatment of pain and other symptoms

☐ In any event, I wish to receive effective treatment of pain and other distressing symptoms such as anxiety, restlessness, breathing difficulties and nausea. I am prepared to accept the reduced awareness (sedation) which such treatment may induce.

or

☐ For me, alertness and the ability to communicate are more important than optimum relief of pain and other symptoms.

III Artificial hydration and nutrition

I consent to *long-term* artificial hydration and nutrition (by nasogastric tube, infusion, surgically implanted feeding tube).

☐ Yes ☐ No

If you answered no

I consent to artificial hydration and nutrition *on a temporary basis*, i.e. if it is expected that my suffering will thereby be relieved or that I will subsequently be able – possibly with support – to ingest fluids and food by the normal route again.

☐ Yes ☐ No

IV Resuscitation in the event of a cardiac and/or respiratory arrest

I wish to be resuscitated.

☐ Yes ☐ No

Additional comments:

____

____

____

____

For peer review only

4. Trusted person / healthcare proxy

☐ I have appointed the following healthcare proxy whom I authorize to make my wishes known to the health care team. This person is to be informed about my condition and involved in decision-making; he/she can access my medical records, and I release all physicians and nursing staff from their duty of confidentiality vis-à-vis this person.

Name, first name ______________________

Address ______________________

Postcode/town ______________________

Tel. home ____________ work ____________ mobile ____________

E-mail ______________________

If my healthcare proxy cannot be contacted or is unable for other reasons to exercise this responsibility, I designate the following substitute:

Name, first name ______________________

☐ I have informed my healthcare proxy about this advance directive.

Name, first name of my general practitioner (GP) ______________________

Address ______________________

Postcode/town ______________________

Tel. home ____________ work ____________ mobile ____________

E-mail ______________________

☐ I have informed my general practitioner (GP) about this advance directive.

5. Special instructions in the event of my death

Organ donation

☐ I wish to donate my organs, and I consent to the removal of any organs, tissue or cells and to the implementation of the preparatory medical measures which are required prior to organ donation. I consent to the use of any intensive measures this may require.

☐ I consent only to the removal of ______________________

☐ I do not wish to be a donor.

Autopsy: I consent to an autopsy

☐ Yes ☐ No ☐ I leave this decision to my healthcare proxy.

I am aware that the drawing-up of an advance directive is voluntary, and that I may revoke or modify it at any time, as long as I have mental capacity.

I have prepared separate supplements to this advance:

☐ No ☐ Yes, namely ______________________

Place, date ______________________ Signature ______________________

N.B. Please keep your advance directive in such a way that it can be found should the need arise; you can leave a copy with your general practitioner. Advance directives should not be sent to the FMH/SAMS.The SAMS guidelines on advance directives are available online at *www.samw.ch,* and further information on advance directives is available at *www.fmh.ch > Patientenverfügung.*

For peer review only

FMH

SAMW ASSM
Schweizerische Akademie der Medizinischen Wissenschaften
Académie Suisse des Sciences Médicales
Accademia Svizzera delle Scienze Mediche
Swiss Academy of Medical Sciences

Advance directive | Short version

Drawn up by

Name, first name ____________________

Date of birth ____________ Place of residence ____________________

If I become incapacitated, it is my wish that:

- ☐ all medically indicated measures (including resuscitation) should be taken to treat the acute condition so that I may regain capacity;

  or

- ☐ I should not be resuscitated and no intensive medical interventions (in particular ventilation) should be carried out;

  or

- ☐ I should not be resuscitated, but I consent to treatment in an intensive care unit.

If, after an initial stabilizing of my condition, it becomes clear that I am unlikely to regain capacity and that there is a high risk that I will require long-term care, it is my wish that:

- ☐ all life-sustaining measures continue to be carried out, provided there is still hope that I may regain capacity;
- ☐ no further life-sustaining measures are carried out.
- ☐ In any event, I wish to receive effective treatment of pain and other distressing symptoms such as anxiety, restlessness, breathing difficulties and nausea.

I have appointed the following healthcare proxy whom I authorize to make my wishes known to the health care team. This person is to be informed about my condition and involved in decision-making; he/she can access my medical records, and I release all physicians and nursing staff from their duty of confidentiality vis-à-vis this person.

Name, first name ____________________

Address ____________________ Postcode/town ____________

Tel. home ____________ work ____________ mobile ____________

E-mail ____________________

If my healthcare proxy cannot be contacted or is unable for other reasons to exercise this responsibility, I designate the following substitute:

Name, first name ____________________

- ☐ I have informed my healthcare proxy about this advance directive.

Name, first name of my general practitioner (GP) ____________________

Address ____________________ Postcode/town ____________

Tel. home ____________ work ____________ mobile ____________

E-mail ____________________

- ☐ I have informed my general practitioner (GP) about this advance directive.

For peer review only

Organ donation

☐ I wish to donate my organs, and I consent to the removal of any organs, tissue or cells and to the implementation of the preparatory medical measures which are required prior to organ donation. I consent to the use of any intensive measures this may require.

☐ I consent only to the removal of ______________________

☐ I do not wish to be a donor.

Place, date ______________________ Signature ______________________

For peer review only

N.B. Please keep your advance directive in such a way that it can be found should the need arise. You can leave a copy with your general practitioner. Advance directives should not be sent to the FMH/SAMS. The SAMS guidelines on advance directives are available online at *www.samw.ch*, and further information on advance directives is available at *www.fmh.ch > Patientenverfügung*.

BMJ Open

Older adults' medical preferences for the end of life: a cross-sectional population-based survey in Switzerland

Journal:	*BMJ Open*
Manuscript ID	bmjopen-2022-071444.R2
Article Type:	Original research
Date Submitted by the Author:	29-Jun-2023
Complete List of Authors:	Vilpert, Sarah; University of Lausanne, Faculty of Business and Economics (HEC)<br>Meier, Clément; University of Lausanne, Faculty of Business and Economics (HEC); University of Lausanne, Faculty of Biology and Medicine<br>Berche, Jeanne; Unisanté<br>Borasio, Gian Domenico; Centre Hospitalier Universitaire Vaudois, Palliative Care Service<br>Jox, Ralf; Centre Hospitalier Universitaire Vaudois Institut des humanités en médecine; Centre Hospitalier Universitaire Vaudois Service de soins palliatifs et de support<br>Maurer, Jurgen; University of Lausanne Faculty of Business and Economics
**Primary Subject Heading**:	Public health
Secondary Subject Heading:	Communication, Palliative care, Patient-centred medicine
Keywords:	Quality of Life, PUBLIC HEALTH, PAIN MANAGEMENT, INTENSIVE & CRITICAL CARE, Aged, Cardiopulmonary Resuscitation

SCHOLARONE™
Manuscripts

I, the Submitting Author has the right to grant and does grant on behalf of all authors of the Work (as defined in the below author licence), an exclusive licence and/or a non-exclusive licence for contributions from authors who are: i) UK Crown employees; ii) where BMJ has agreed a CC-BY licence shall apply, and/or iii) in accordance with the terms applicable for US Federal Government officers or employees acting as part of their official duties; on a worldwide, perpetual, irrevocable, royalty-free basis to BMJ Publishing Group Ltd ("BMJ") its licensees and where the relevant Journal is co-owned by BMJ to the co-owners of the Journal, to publish the Work in this journal and any other BMJ products and to exploit all rights, as set out in our licence.

The Submitting Author accepts and understands that any supply made under these terms is made by BMJ to the Submitting Author unless you are acting as an employee on behalf of your employer or a postgraduate student of an affiliated institution which is paying any applicable article publishing charge ("APC") for Open Access articles. Where the Submitting Author wishes to make the Work available on an Open Access basis (and intends to pay the relevant APC), the terms of reuse of such Open Access shall be governed by a Creative Commons licence – details of these licences and which Creative Commons licence will apply to this Work are set out in our licence referred to above.

Other than as permitted in any relevant BMJ Author's Self Archiving Policies, I confirm this Work has not been accepted for publication elsewhere, is not being considered for publication elsewhere and does not duplicate material already published. I confirm all authors consent to publication of this Work and authorise the granting of this licence.

For peer review only

Older adults' medical preferences for the end of life: a cross-sectional population-based survey in Switzerland

Abstract:

Objectives: Medical decision-making at the end of life is common and should be as patient-centered as possible. Our study investigates older adults' preferences toward three medical treatments that are frequently included in advance directive forms and their association with social, regional, and health characteristics.

Setting: A cross-sectional study using population-based data of wave 8 (2019/20) of the Swiss component of the Survey of Health, Ageing, and Retirement in Europe (SHARE).

Participants: 1,430 adults aged 58 years and older living in Switzerland.

Primary and secondary outcome measures: Three questions on the preferences regarding cardiopulmonary resuscitation (CPR); life-prolonging treatment in case of high risk of permanent mental incapacity; reduced awareness (sedation) to relieve unbearable pain and symptoms. Their associations with individuals' social, regional and health characteristics.

Results: Most older adults expressed a wish to receive CPR (58.6%) and to forgo life-prolonging treatment in case of permanent mental incapacity (90.7%). Most older adults also indicated that they would accept reduced awareness if necessary to receive effective treatment for pain and distressing symptoms (60.8%). Older adults' treatment preferences for CPR and life-prolonging treatment differed according to sex, age, partnership status, linguistic region and health status, while willingness to accept reduced awareness for effective symptom treatment was more similarly distributed across population groups.

Conclusions: Simultaneous preferences for CPR and refusal of life-prolonging treatment might appear to be conflicting treatment goals. Considering individuals' values and motivations can help clarify ambivalent treatment decisions. Structured ACP processes with trained professionals allows for exploring individuals' motivations and values and helps to identify congruent care and treatment goals.

Strengths and limitations of this study

- This study replicates the questions on treatment preferences that are usually part of advance directives.
- This study explores treatment preferences in a national population-based sample of older adults.
- The wording of the questions may not provide clear indications of individuals' actual treatment preferences.

Keywords

Advance directives; life-prolonging treatments; cardiopulmonary resuscitation; sedation; population-based study

For peer review only - http://bmjopen.bmj.com/site/about/guidelines.xhtml

Introduction:

Advances in medical technologies and extended life expectancy have changed the context of dying in today's societies (1) and often result in a need to make complex medical decisions at the end of life (EOL) (2,3). EOL decisions may concern pain or symptom management, possibly but not necessarily with life-shortening effects, withholding or withdrawing life-supporting treatment (e.g. cardiopulmonary resuscitation (CPR), artificial nutrition and hydration, mechanical ventilation, dialysis), and, in countries where it is legal, euthanasia and assisted suicide (4,5). Following the principle of patient autonomy (6), EOL decisions are jointly made by the patients and/or their surrogates and clinicians (7). These decisions ought to be based on considerations of prognosis, benefits and risks of different treatments, and patients' values and care goals among others (8). Patients commonly opt for or against certain treatments based on their perceived benefits or costs in terms of quality of life and prolongation of life, while physicians typically consider the medical appropriateness of the treatments under consideration.

EOL decisions are most often the result of multiple discussions between medical experts, patients, and potentially their families which constitute the basis of a process called advance care planning (ACP), when preparing for a patient health situation that may worsen in the future. The ACP outcomes may be recorded in advance directives (ADs). ACP aims to enable patients to receive medical treatments that are better aligned with their values, care preferences and goals (9). ACP thus contributes to maintaining patients' quality of life throughout their care episodes (10), and eventually achieve what they may have defined as a "good death" for themselves during the ACP process (11,12). ADs record the medical treatments to which a person consents or refuses. ADs can also be used to designate a healthcare proxy to make medical decisions on behalf of that person in the event of a loss of decision-making capacity (13). At the end of life, more than half of the patients lose their decision-making capacity (4). While ACP may not always entirely achieve its primary goals such as a positive impact on patients' quality of life or EOL care consistent with patient preference and goals of care, it often achieves other important secondary objectives such as improving the quality of patient-physician communication and congruence in preferences between patients and their caregivers as well as reducing decisional conflict, increasing preferences for comfort care, and encouraging the documentation of ACP and ADs (14,15). Furthermore, the existence of ADs is associated with more out-of-hospital care, an increased focus on comfort care, and may contribute to reducing overtreatment at the end of life (16).

In Switzerland, ADs have been legally binding since 2013 and individuals can find numerous AD forms published by medical associations, patient organizations or other private initiatives. Most AD forms include information on

For peer review only - http://bmjopen.bmj.com/site/about/guidelines.xhtml

medical and health situations in which the wishes expressed in ADs are to be applied (individuals' treatment preferences, organ donation, and designated healthcare proxy), while some of them also contain a section about personal values, the meaning of life and quality of life and related fears and expectations (17). ADs can be completed by individuals alone, although completion with the assistance of trained professionals or healthcare specialists is generally recommended (18). While many people tend to have the impression that ADs are mainly targeted to older adults and persons with terminal illnesses, AD completion is in fact also recommended for healthy younger adults, due to the broader risks of potential loss of decision-making capacity after, for example, an accident (19).

Understanding treatment preferences commonly indicated in AD forms may be helpful to assess attitudes towards EOL medical decisions in the general population and give insights into how to best initiate discussion on the topic. Preferences regarding specific life-sustaining treatments (20–22), general EOL care goals such as priority for length or quality of life (21,23,24), and palliative sedation therapy (25–29) have been studied in the general population, but mostly in hypothetical contexts of severe physical pain or cognitive decline. To our knowledge, these preferences have not been investigated together in the way in which they might appear in AD forms. We, therefore, consider three specific questions taken from the actual AD form (see Appendix 1) used until 2022 by the Swiss Medical Association FMH, the leading physicians' association in Switzerland, which comprises 42,000 members and over 70 medical organisations in the country. In this study, we examine older adults' preferences for CPR, life-prolonging treatment, and reduced awareness (sedation) as measured by the questions from the aforementioned AD form in a large population survey of adults aged 58 years and older living in Switzerland. We also investigate the association between stated treatment preferences and individuals' social, regional, and health characteristics. This information is relevant to understanding how older adults view specific EOL treatments and identifying differences in treatment preferences among the population groups.

Methods:

We used data from the Survey of Health, Ageing and Retirement in Europe (SHARE), which collects internationally comparable multidisciplinary longitudinal microdata on social, economic and health conditions of European aged 50 and over and their partners (30). We used a subsample of the Swiss-SHARE Wave 8 (2019/2020) dataset, which included respondents who participated in the internationally harmonized in-person interview and the national paper-and-pencil self-administered EOL questionnaire distributed at the end of the interview in Switzerland (31,32). The EOL questionnaire was developed by the Swiss SHARE team and palliative

For peer review only - http://bmjopen.bmj.com/site/about/guidelines.xhtml

care experts of the Lausanne University Hospital and concerns preferences, knowledge, attitudes, and behaviors toward EOL care planning. In Wave 8 in Switzerland, 2,005 respondents participated in the SHARE main interview and 94.3% of them (1,891 individuals) also completed the EOL questionnaire. Since the last sample refreshment in Switzerland was in 2011, only 28 respondents to the Swiss-SHARE Wave 8 survey, which had entered the study as partners, were aged 50-57. We, therefore, discard these respondents from our sample and limit our analyses to respondents aged 58 years and older who had no missing values on any of the items used in our analysis. Our selected final analytical sample comprises 1,430 respondents (Figure1).

(Insert figure 1 here)

Measures

Outcome variables:

We included three questions in our EOL questionnaire, which have been adapted directly from the long version of the AD form that was publicly available for download from the Swiss Medical Association FMH website. The adaptations made for inclusion in our survey mainly consisted of transforming the statements into questions, while keeping the vocabulary and contextual information provided as in the Swiss Medical Association's AD forms.

Question 1. Imagine that you experience a cardiac and/or respiratory arrest. In this situation, you wish:

- to be resuscitated (1=accept CPR).
- not to be resuscitated (0=refuse CPR)

Question 2. Imagine that you are incapacitated following an accident, a stroke, or a heart attack. After initial emergency measures and careful medical assessment, physicians deem it very unlikely that you will regain mental capacity. In this situation, you prefer:

- to forgo all measures which would only serve to prolong your life and suffering (0=refuse life-prolonging treatment)
- that, despite the poor outlook, every medically appropriate measure should be taken ((1=accept life-prolonging treatment).

Question 3. Imagine that you suffer from a disease that causes unbearable pain and symptoms such as fear, restlessness, breathing difficulties, and nausea. In this situation:

For peer review only - http://bmjopen.bmj.com/site/about/guidelines.xhtml

For peer review only

- you wish to receive optimal treatment of pain and other distressing symptoms, and you are prepared to accept the reduced awareness (sedation) that such treatment may induce (1=accept reduced awareness)
- for you, alertness and the ability to communicate are more important than optimal relief of pain and other symptoms (0=refuse reduced awareness).

Independent variables:

We considered social, regional and health individuals' characteristics as independent variables in our study. Social characteristics included information on sex (0=male, 1=female), age group (58-64 years, 65-74 years, 75+ years), education level based on the International Standard Classification of Education (ISCED) of 2017 (33) (basic=ISCED levels 0-1-2, secondary=ISCED levels 3-4, tertiary=ISCED levels 5-6), living with a partner (0=has a partner, 1=has no partner), parenthood (0=no children, 1=at least one child) and self-perceived financial difficulties (Is your household able to make ends meet?: 1=easily, 2=fairly easily, 3=with difficulty). Regional characteristics comprised variables on the living environment (0=urban, 1=rural), language regions (German, French or Italian) and religiosity (0=never pray, 1=pray at least sometimes). Health characteristics involved two variables: self-rated health (1 = poor/fair health, 2 = good health, 3 = very good/excellent health) and self-reported limitation in activities of daily living (ADLs) (0=no limitation, 1=at least one limitation).

Statistical analysis:

We performed proportion estimation for all the variables included in the study. We further used probit regressions to assess the partial associations of preferences for medical treatments with respondents' characteristics. Regression estimates are expressed in terms of average partial effects (APEs). APEs report (in percentage points) how the average conditional outcome probability $P(Y=1 \mid X)$ changes when the given explanatory variable changes from 0 to 1, holding all other independent variables at their observed values. Estimated standard errors were clustered at the household level to account for potential unobserved dependencies between partners' responses. All estimations were conducted using STATA/SE 17.0 software (STATA Corporation, College Station, TX).

Ethical approval

The study was approved by the research ethics committee of the canton of Vaud in March 2014 with number 66/14. In addition, during data collection, respondents gave their consent to participate in the survey on two occasions, when they agreed over the phone to schedule an interview and at the beginning of the in-person interview.

For peer review only - http://bmjopen.bmj.com/site/about/guidelines.xhtml
For peer review only

Patient and Public Involvement

Patients and the public were not directly involved in the design, conduct, reporting or dissemination of this study as it uses SHARE data (secondary data) and direct public contribution was therefore not possible.

Results:

The social, cultural, and health characteristics of the study population are presented in Table 1. Regarding preferences for medical treatments displayed in Figure 2, 58.6% of respondents preferred to receive CPR, 90.7% chose to forgo life-prolonging treatment in case of high risk of permanent mental incapacity, and 60.8% accepted reduced awareness for optimum treatment of pain and other distressing symptoms.

For peer review only

For peer review only - http://bmjopen.bmj.com/site/about/guidelines.xhtml

Table 1: Characteristics of the study sample, adults aged 58+, SHARE Switzerland, 2019/2020, n=1,430		
	Number	Proportions (%)
Gender		
Male	676	47.3
Female	754	52.7
Age groups		
58-64 years	351	24.6
65-74 years	603	42.2
75+ years	476	33.3
Education		
Basic	231	16.2
Secondary	911	63.7
Tertiary	288	20.1
Living with a partner		
Yes	1,071	74.9
No	359	25.1
Parenthood		
No children	221	15.5
At least on child	1,209	84.5
Make ends meet		
Easily	798	55.8
Fairly easily	457	32
With difficulty	175	12.2
Living environment		
Urban	657	45.9
Rural	773	54.1
Switzerland linguistic regions		
German	1,037	72.5
French	345	24.1
Italian	48	3.4
Religiosity		
Never pray	441	30.8
Pray at least sometimes	989	69.2
Self-rated health		
Poor/fair health	250	17.5
Good health	607	42.4
Very good/excellent health	573	40.1
Self-reported limitations in activities of daily living (ADLs)		
No limitation	1,343	93.9
At least one limitation	87	6.1

(Insert Figure 2 here)

For peer review only - http://bmjopen.bmj.com/site/about/guidelines.xhtml

Table 2 shows the results from the multivariable probit regressions of preferences for the three different medical treatments on respondents' characteristics. Concerning preferences for CPR, being a woman decreases the probability of wanting to be resuscitated by 11.2 percentage points compared to being a man (APE: -11.2, $p < 0.001$). In addition, living without a partner (APE: -8.2; $p < 0.05$) and being older than the reference age group of 58-64 (APE 65-74: -10.1, $p < 0.01$; APE 75+: -20.4, $p < 0.001$) were also each associated with a lower likelihood of wanting to be resuscitated. In contrast, having a tertiary level of education (APE: 10.3, $p < 0.05$), being from the Italian-speaking part of Switzerland (APE: 24.8, $p < 0.001$), praying at least sometimes (APE: 5.6, $p < 0.01$), and self-rating one's health as good (APE: 9.4, $p < 0.05$) or very good/excellent (APE: 12.0, $p < 0.01$) were positively associated with preferences for CPR.

Regarding general preferences for life-prolonging treatment, being a woman (APE: -4.3, $p < 0.01$), being 65- to 74 years old (APE: -3.8, $p < 0.05$), and having at least one limitation in activities of daily living (APE: -4.8, $p < 0.05$) were all negatively associated with preference for life-prolonging treatment in case of high risk of permanent mental incapacity. On the other side, living without a partner (APE: 4.3, $p < 0.01$) and being from the Italian-speaking part of Switzerland (APE: 17.7, $p < 0.01$) were associated with a higher chance of wanting life-prolonging treatment.

Concerning acceptance of reduced awareness, having a secondary level of education (APE: 8.9, $p < 0.01$) was associated with a higher likelihood of accepting reduced awareness for optimum pain treatment and other distressing symptoms. At the same time, respondents who said they pray at least sometimes (APE: -6.2, $p < 0.01$) were less likely to accept reduced awareness in order to receive optimal treatment of pain and other distressing symptoms.

For peer review only

Table 2: Partial associations of preferences for medical treatments with respondents' social, cultural, and health characteristics, adults aged 58+, SHARE Switzerland, 2019/2020, n=1,430			
	Preference for CPR APE (95% CI)	Preference for life-prolonging treatment APE (95% CI)	Preference for reduced awareness APE (95% CI)
Gender (ref: male)			
Female	-11.2*** (-16.2,-6.3)	-4.3** (-7.3,-1.4)	2.3 (-2.9,7.4)
Age groups (ref: 58-64 years)			
65-74 years	-10.1** (-16.3,-4.0)	-3.8* (-7.6,-0.1)	2.2 (-4.2,8.5)
75+ years	-20.4*** (-27.3,-13.5)	-4.0 (-8.0,0.0)	-5.1 (-12.4,2.2)
Education (ref: basic)			
Secondary	1.2 (-6.0,8.4)	-4.1 (-8.8,0.7)	8.9* (1.4,16.3)
Tertiary	10.3* (1.5,19.1)	-4.1 (-9.5,1.4)	7.6 (-1.8,16.9)
Living with a partner (ref: yes)			
No	-8.2* (-14.4,-1.9)	4.3* (0.5,8.1)	2.2 (-4.1,8.5)
Parenthood (ref: no children)			
At least on child	0.6 (-6.5,7.7)	-0.6 (-4.5,3.2)	3.3 (-3.9,10.5)
Ease of making ends meet (ref: easy)			
Fairly easily	1.6 (-4.2,7.4)	1.1 (-2.0,4.3)	-2.4 (-8.4,3.7)
With difficulty	2.2 (-6.0,10.3)	2.9 (-2.2,8.1)	-0.0 (-8.7,8.6)
Living environment (ref: urban)			
Rural	1.4 (-3.8,6.6)	0.3 (-2.5,3.0)	-0.0 (-5.4,5.3)
Switzerland linguistic regions (ref: German)			
French	-6.1 (-12.3,0.0)	2.3 (-1.1,5.7)	2.6 (-3.5,8.7)
Italian	24.8*** (15.2,34.3)	17.7* (4.0,31.4)	-7.5 (-23.5,8.5)
Religiosity (ref: never pray)			
Pray at least sometimes	5.6* (0.1,11.1)	0.5 (-2.5,3.5)	-6.2* (-11.8,-0.5)
Self-rated health (ref: poor/fair health)			
Good health	9.4* (1.8,16.9)	-2.4 (-6.6,1.8)	2.6 (-4.8,10.0)
Very good/excellent health	12.0** (4.0,20.0)	-1.9 (-6.3,2.5)	-3.3 (-11.4,4.7)
Self-reported limitations in activities of daily living (ADLs) (ref: no limitation)			
At least one limitation	-1.7 (-12.7,9.4)	-4.8* (-8.6,-0.9)	0.9 (-10.4,12.3)
Observations	1,430	1,430	1,430

*CPR=Cardiopulmonary resuscitation. Average partial effects (APEs) based on probit regression models. All probabilities are multiplied by 100 to express APEs in percentage points. Asterisks indicate levels of significance: ***<0.1%, ** 1%, *5%. Interpretation of APEs: Being a woman decreases the probability of wanting to be resuscitated by 11.2 percentage points compared to being a man. For variables with three response categories the interpretation is as follows. Being aged 75 and over reduces the probability of wanting to be resuscitated by 20.4 percentage points in comparison with being aged 58-64 (reference category).*

For peer review only

Discussion

Our study is the first national population-based study in Switzerland that investigates older adults' preferences toward specific medical treatments that are commonly elicited in official AD forms. Most respondents wished to receive CPR in case of a cardiac/respiratory arrest, to forgo life-prolonging treatment in case of high risk of permanent mental incapacity, and to reduce their awareness for optimal treatment of pain and other distressing symptoms. Additionally, significant social, regional, and health-related differences in medical treatment preferences were found for CPR and life-sustaining treatments, while preferences for reduced awareness were more uniformly distributed across different population groups.

Preference for cardiopulmonary resuscitation

A majority of individuals reported wanting to receive CPR in the event of cardiac and/or respiratory arrest. In most population-based studies investigating preferences for CPR, the question is asked for a given health condition (e.g., terminal cancer, advanced dementia), and individuals largely refuse CPR (20,21,25). There are several reasons that could explain this difference between our study and previous ones. In our study, the context of the CPR is not specified, and respondents may be referring to their current health status, which is generally rather good (84.8% declared a good, very good, or excellent health) with the preference for CPR being potentially influenced by the prospect of significant remaining longevity (24). Alternatively, this difference could also be explained by an overestimation of CPR survival rate (34), which may lead individuals to choose CPR (35). Some individuals may decide against CPR once they have been informed of its procedure, risks and benefits in different contexts (36).

Regarding social differences in CPR preferences in our study, women reported lower preferences for CPR than men. This finding, which has been shown in other studies (21,25), could be explained by a higher valuation of quality of life over length of life among women compared to men (37) which may lead them to refrain from treatments (38). Moreover, women are more likely to have direct experience with caregiving (39) that may make them more aware of the benefits and limitations of certain medical treatments (34) and the potential burden that these treatments may imply for patients and their relatives (24). Respondents living without a partner were also less likely to opt for CPR than respondents living with a partner. Since respondents living without a partner also seem to be better informed about the limited success of CPR, as shown in a previous study by the authors based on the same Swiss subsample of SHARE Wave 8 (34), they may prefer to refuse CPR (40).

For peer review only - http://bmjopen.bmj.com/site/about/guidelines.xhtml

Conversely, respondents with a tertiary level of education were more likely to report a preference for CPR. Our previous study (34) showed that older adults with higher levels of education were not more knowledgeable about the (relatively low) chances of success of CPR and may, therefore, not be more likely to forgo CPR than persons with less education (34). A study of hospitalized patients aged 65 and over in New Zealand reported that more educated patients were more likely to prefer CPR in their current state of health (41). One potential reason could be that individuals with higher education believe to have better health prospects (42) which could induce them to opt for CPR. One study showed that older adults who are optimistic about their own life expectancy are more likely to want to continue all medical treatment in a situation of serious illness even if there are very low chances of survival (24). Acceptance of CPR was also higher in the Italian linguistic region and among more religious people, who report to pray at least sometimes. These findings may highlight well-documented cultural differences in Switzerland, with respondents from the Italian-speaking part (43,44) and more religious persons being more likely to be influenced by religious considerations and corresponding beliefs in the sanctity of life (38,44,45) that tend to encourage life-prolonging treatments (11).

Finally, age and self-rated health status, which are two parameters of medical prognoses, were also strongly associated with CPR preferences. Compared to adults aged 58-64, older respondents were less likely to state that they would prefer to receive CPR, with this negative association increasing in age. These results are consistent with other studies showing that older people are more likely to refuse CPR (21,25,46). Older adults might realize that age decreases their chances of surviving a CPR (47) and, therefore, be more likely to reject this treatment. The same reasoning may also apply to people in poorer health (48).

Preference for life-prolonging treatment

Over 90% of our respondents indicated a preference to forgo life-prolonging treatment if they were to become incapacitated following an accident, a stroke, or a heart attack and were very unlikely to regain mental capacity. Similar results were found in studies of preferences to continue or stop treatment in the presence of serious physical or mental illness in the general population (21,24). Interestingly, the population groups more likely to forgo life-prolonging treatments are comparable to those who refused CPR, that is, women, older persons, and those in poorer health (1+ ADLs). These findings are in line with a study of the general Dutch population that showed that women, older people, and people with a history of serious illness tend to prioritize quality of life over quantity (38). In contrast, Swiss participants from the Italian-speaking region who were more likely to prefer CPR were also more likely to prefer life-prolonging treatment. A surprising result for which we have no explanation is the preference

For peer review only - http://bmjopen.bmj.com/site/about/guidelines.xhtml

for life-sustaining treatments among people living without a partner, especially since many of them simultaneously indicated to refuse CPR.

Preference for reduced awareness

In our study, 60.8% of our respondents accepted reduced awareness in a situation where they would suffer from a disease that causes unbearable pain and symptoms such as fear, restlessness, breathing difficulties, and nausea, while for the remaining respondents (39.2%), awareness and the ability to communicate was more important than optimal relief of pain and other symptoms. Other studies of individuals' preferences for different types of palliative sedation (e.g. intermittent, mild, continuous deep sedation), in different situations and for different durations have produced mixed results, which are not directly comparable to ours (25–29). Interestingly, although adequate pain and symptoms management is one of the most important features of a good death (28), preservation of consciousness also plays a central role at the end of life for many people (11,25,27). In a Dutch study, 61% of adults aged 20+ reported being conscious until death as important. Those individuals were less likely to accept terminal sedation (67%) and the use of high dosages of morphine (67%) (28). These findings highlight the clinical importance to clearly discuss the possible interaction and trade-offs between symptom alleviation and decreased alertness with patients.

Only education and religiosity were associated with individuals' preferences for or against reduced awareness as a side effect of optimal symptom treatment. People with secondary education are more likely to accept reduced awareness, which is consistent with a Belgian study that showed that patients with higher levels of education are more likely to emphasize pain and symptom relief at the end of life (49). Furthermore, more religious respondents were less likely to accept reduced awareness. Another study pointed that the proportion of individuals rejecting gradual and rapid sedation was higher among more religious people (29). These associations may be linked to the belief that symptom treatment that reduces awareness also shortens life (50), especially since religiosity is commonly associated with life-prolonging treatment preferences among older adults (21,24,38).

Potential discrepancy in treatment preferences

Our results may at first seem contradictory in that most respondents indicated a preference against life-prolonging treatments but at the same time showed a preference for CPR, which has a relatively low chance of survival. This type of ambivalence has already been noted in a study that showed that about one-third of the Dutch adult population could not make a clear choice between quality and quantity of life (38), which could be compared in our study with the refusal of EOL treatments (quality of life) and the simultaneous preference for resuscitation

For peer review only - http://bmjopen.bmj.com/site/about/guidelines.xhtml

(quantity of life). Another study also pointed to inconsistencies between Dutch older adults' general EOL goals and preferences for specific life-sustaining treatments (20). Their authors explained these results by the lack of knowledge of the risks and side effects of these treatments in the population.

The apparent contradiction between preferences for CPR and life-prolonging treatments in our study may be related to the framing of our questions. Our CPR question provides no information about the context in which the CPR is to be conducted such as whether the CPR would take place in- or out-hospital, and individual's health status is also not specified in this question. By contrast, the question on life-prolonging treatment refers to a situation of very bad health which may explain why the overwhelming majority of respondents reported a preference to refuse life-prolonging treatment in this context. Both questions were directly drawn from the Swiss Medical Association's AD form, which was in use at the time of our survey, but has been revised since. The long version of this form contains supplementary contextual information on individuals' motivations, health history and social situation, which seems helpful in translating patients' values and preferences into medically actionable information. Nevertheless, this supplementary information may not completely prevent potential conflicting information about individuals' treatment preferences in case of patients' loss of decision capacity, especially if this information may stand in contrast to individuals' treatment wishes as indicated by the check box questions. A broader approach to ACP that can be translated into more nuanced ADs is clearly desirable and increasingly implemented in practice (10).

Limitations

One limitation of our study is that our question wording may not be ideal for eliciting people's treatment preferences even if the questions were highly relevant to Swiss EOL policy at the time of our survey due to their close correspondence with a widely known AD form in Switzerland. Another limitation is that people in very poor health or living in a nursing home may be underrepresented in our study, despite the considerable efforts of SHARE to interview also sicker people and follow respondents into institutions such as nursing homes. On the other hand, the EOL questionnaire's cooperation rate was high (94.3%), and an analysis of the profile of non-respondents based on individuals' characteristics used in our regression models indicated no obvious underrepresentation of certain population groups.

For peer review only

For peer review only - http://bmjopen.bmj.com/site/about/guidelines.xhtml

Conclusion

Our study investigates preferences for CPR, life-prolonging treatments, and reduced awareness in the general population of adults aged 58 years and older living in Switzerland based on three statements included in the FMH Swiss Medical Association's AD form. Most older adults wished to receive CPR in case of a cardiac/respiratory arrest, forgo life-prolonging treatment in case of high risk of permanent mental incapacity, and reduce their awareness for optimal treatment of pain and other distressing symptoms. Our study also revealed that individuals' preferences appear to be influenced by characteristics that affect medical prognoses, such as advanced age and poor health. Moreover, women were less likely to report a preference for CPR and life-prolonging treatments than men. Furthermore, the apparent contradiction in our findings between individuals' willingness to receive CPR and their wishes to forgo life-prolonging treatment may reveal knowledge gaps regarding the success chances and side effects of certain treatments and also highlights the potential ambivalence between individuals' EOL care goals and the choice they make for specific EOL treatments. This inherent complexity of making advance decisions in the context of ADs could benefit from further contextual information and elicitation of individuals' values through processes of ACP, which facilitate a clearer understanding of individuals' values, motivations and fears concerning EOL care that can help to better translate ADs and related information into medical decisions at the EOL.

Contributorship statement

SV designed the study. CM and JB conducted the statistical analysis with the support of SV. SV, CM and JM drafted the manuscript. All authors discussed the interpretation of findings and provided critical revision of the manuscript for important intellectual content.

Competing interests

The authors declare that they have no competing interests.

Funding

This work was supported by the Swiss National Science Foundation (SNSF), grant number 10001C_188836.

This paper uses data from Börsch-Supan, A. (2021). Survey of Health, Ageing and Retirement in Europe (SHARE) Wave 8. Release version: 1.0.0. SHARE-ERIC. Data set. DOI: 10.6103/SHARE.w8.100. The SHARE data collection has been funded by the European Commission, DG RTD through FP5 (QLK6-CT-2001-00360), FP6

For peer review only - http://bmjopen.bmj.com/site/about/guidelines.xhtml

(SHARE-I3: RII-CT-2006-062193, COMPARE: CIT5-CT-2005-028857, SHARELIFE: CIT4-CT-2006-028812), FP7 (SHARE-PREP: GA N°211909, SHARE-LEAP: GA N°227822, SHARE M4: GAN°261982, DASISH: GA N°283646) and Horizon 2020 (SHARE-DEV3: GA N°676536, SHARE-COHESION: GA N°870628, SERISS: GA N°654221, SSHOC: GA N°823782) and by DG Employment, Social Affairs & Inclusion through VS 2015/0195, VS 2016/0135, VS 2018/0285, VS 2019/0332, and VS 2020/0313. Additional funding from the German Ministry of Education and Research, the Max Planck Society for the Advancement of Science, the U.S. National Institute on Aging (U01_AG09740-13S2, P01_AG005842, P01_AG08291, P30_AG12815, R21_AG025169, Y1-AG-4553-01, IAG_BSR06-11, OGHA_04-064, HHSN271201300071C, RAG052527A) and from various national funding sources is gratefully acknowledged (see https://share-eric.eu/).

Data sharing statement

SHARE data are publicly available for scientific purposes only through registration on the data distribution platform.

Ethics Approval

The study was approved by the research ethics committee of the canton of Vaud in March 2014 with number 66/14.

For peer review only

References

1. Etkind SN, Bone AE, Gomes B, Lovell N, Evans CJ, Higginson IJ, et al. How many people will need palliative care in 2040? Past trends, future projections and implications for services. BMC Med. 2017 May 18;15(1):102.

2. Bosshard G, Zellweger U, Bopp M, Schmid M, Hurst SA, Puhan MA, et al. Medical End-of-Life Practices in Switzerland: A Comparison of 2001 and 2013. JAMA Intern Med. 2016 Apr;176(4):555–6.

3. Becker C, Künzli N, Perrig S, Beck K, Vincent A, Widmer M, et al. Code status discussions in medical inpatients: results of a survey of patients and physicians. Swiss Med Wkly. 2020 Mar 23;150(1314).

4. van der Heide A, Deliens L, Faisst K, Nilstun T, Norup M, Paci E, et al. End-of-life decision-making in six European countries: descriptive study. The Lancet. 2003 Aug 2;362(9381):345–50.

5. Löfmark R, Nilstun T, Cartwright C, Fischer S, van der Heide A, Mortier F, et al. Physicians' experiences with end-of-life decision-making: Survey in 6 European countries and Australia. BMC Med. 2008 Feb 12;6(1):4.

6. Principles of Biomedical Ethics - Paperback - Tom L. Beauchamp; James F. Childress - Oxford University Press [Internet]. [cited 2022 Nov 16]. Available from: //global.oup.com/ushe/product/principles-of-biomedical-ethics-9780190640873

7. Rosca A, Krones T, Biller-Andorno N. Shared decision making: patients have a right to be informed about possible treatment options and their risks and benefits. Swiss Med Wkly. 2020 Jul 6;150(2728):w20268–w20268.

8. Mehta RS, Anderson WG, Hunt S, Chaitin EK, Arnold RM. Chapter 22 - Withholding and Withdrawing Life-Sustaining Therapies. In: Emanuel LL, Librach SL, editors. Palliative Care (Second Edition) [Internet]. Saint Louis: W.B. Saunders; 2011 [cited 2023 Apr 25]. p. 300–18. Available from: https://www.sciencedirect.com/science/article/pii/B9781437716191000226

9. Sudore RL, Lum HD, You JJ, Hanson LC, Meier DE, Pantilat SZ, et al. Defining Advance Care Planning for Adults: A Consensus Definition From a Multidisciplinary Delphi Panel. J Pain Symptom Manage. 2017 May;53(5):821-832.e1.

10. Rietjens JAC, Sudore RL, Connolly M, van Delden JJ, Drickamer MA, Droger M, et al. Definition and recommendations for advance care planning: an international consensus supported by the European Association for Palliative Care. Lancet Oncol. 2017 Sep;18(9):e543–51.

11. Borrat-Besson C, Vilpert S, Borasio GD, Maurer J. Views on a "Good Death": End-of-Life Preferences and Their Association With Socio-Demographic Characteristics in a Representative Sample of Older Adults in Switzerland. OMEGA - J Death Dying. 2020 Jul 29;

12. Krikorian A, Maldonado C, Pastrana T. Patient's Perspectives on the Notion of a Good Death: A Systematic Review of the Literature. J Pain Symptom Manage. 2020 Jan;59(1):152–64.

13. Pautex S, Herrmann F, Zulian G. Role of advance directives in palliative care units: a prospective study. Palliat Med. 2008 Oct 1;22(7):835–41.

14. Malhotra C, Shafiq M, Batcagan-Abueg APM. What is the evidence for efficacy of advance care planning in improving patient outcomes? A systematic review of randomised controlled trials. BMJ Open. 2022 Jul 1;12(7):e060201.

15. Jimenez G, Tan WS, Virk AK, Low CK, Car J, Ho AHY. Overview of Systematic Reviews of Advance Care Planning: Summary of Evidence and Global Lessons. J Pain Symptom Manage. 2018 Sep;56(3):436-459.e25.

16. Brinkman-Stoppelenburg A, Rietjens JA, van der Heide A. The effects of advance care planning on end-of-life care: A systematic review. Palliat Med. 2014 Sep 1;28(8):1000–25.

For peer review only - http://bmjopen.bmj.com/site/about/guidelines.xhtml

17. Rüegger H. Patientenverfügungen in der deutschsprachigen Schweiz, Institut Neumünster, CURAVIVA. 2011;45.

18. Medical-ethical Guidelines [Internet]. [cited 2023 Apr 24]. Medical-ethical Guidelines. Available from: https://www.samw.ch

19. Otte I, Jung C, Elger B, Bally K. Advance directives and the impact of timing. A qualitative study with Swiss general practitioners. Swiss Med Wkly [Internet]. 2014 Sep 21 [cited 2022 Apr 1];(39). Available from: https://smw.ch/article/doi/smw.2014.14035

20. Evans N, Pasman HR, Deeg D, Onwuteaka-Philipsen B, EURO IMPACT. How do general end-of-life treatment goals and values relate to specific treatment preferences? a population-based study. Palliat Med. 2014 Dec;28(10):1206–12.

21. van Wijmen MPS, Pasman HRW, Widdershoven GAM, Onwuteaka-Philipsen BD. Continuing or forgoing treatment at the end of life? Preferences of the general public and people with an advance directive. J Med Ethics. 2015 Aug;41(8):599–606.

22. Hamano J, Hanari K, Tamiya N. End-of-life care preferences of the general public and recommendations of healthcare providers: a nationwide survey in Japan. BMC Palliat Care. 2020 Mar 24;19(1):38.

23. Chung RYN, Wong ELY, Kiang N, Chau PYK, Lau JYC, Wong SYS, et al. Knowledge, Attitudes, and Preferences of Advance Decisions, End-of-Life Care, and Place of Care and Death in Hong Kong. A Population-Based Telephone Survey of 1067 Adults. J Am Med Dir Assoc. 2017 Apr 1;18(4):367.e19-367.e27.

24. Carr D, Moorman SM. End-of-Life Treatment Preferences Among Older Adults: An Assessment of Psychosocial Influences. Sociol Forum Randolph NJ. 2009 Dec 1;24(4):754–78.

25. Sanjo M, Miyashita M, Morita T, Hirai K, Kawa M, Akechi T, et al. Preferences regarding end-of-life cancer care and associations with good-death concepts: a population-based survey in Japan. Ann Oncol Off J Eur Soc Med Oncol. 2007 Sep;18(9):1539–47.

26. Bérubé A, Tapp D, Dupéré S, Plaisance A, Bravo G, Downar J, et al. Do Socioeconomic Factors Influence Knowledge, Attitudes, and Representations of End-of-Life Practices? A Cross-Sectional Study. J Palliat Care. 2022 Oct 13;8258597221131658.

27. Morita T, Hirai K, Okazaki Y. Preferences for Palliative Sedation Therapy in the Japanese General Population. J Palliat Med. 2002 Jun;5(3):375–85.

28. Rietjens JA, van der Heide A, Onwuteaka-Philipsen BD, van der Maas PJ, van der Wal G. Preferences of the Dutch general public for a good death and associations with attitudes towards end-of-life decision-making. Palliat Med. 2006 Oct 1;20(7):685–92.

29. Takla A, Savulescu J, Kappes A, Wilkinson DJC. British laypeople's attitudes towards gradual sedation, sedation to unconsciousness and euthanasia at the end of life. PLOS ONE. 2021 Mar 26;16(3):e0247193.

30. Börsch-Supan A, Brandt M, Hunkler C, Kneip T, Korbmacher J, Malter F, et al. Data Resource Profile: The Survey of Health, Ageing and Retirement in Europe (SHARE). Int J Epidemiol. 2013 Aug 1;42(4):992–1001.

31. [dataset] Börsch-Supan, A. Data from: Survey of Health, Ageing and Retirement in Europe (SHARE) Wave 8. Release version: 8.0.0. SHARE-ERIC, 10th February 2022. DOI: 10.6103/SHARE.w8.800.

32. Bergmann M, Börsch-Supan A (Eds.) (2021). SHARE Wave 8 Methodology: Collecting Cross-National Survey Data in Times of COVID-19. Munich MEA Max Planck Inst Soc Law Soc Policy.

33. Hoffmeyer-Zlotnik JHP, Hoffmeyer-Zlotnik JHP, Wolf C. Advances in Cross-National Comparison: A

For peer review only - http://bmjopen.bmj.com/site/about/guidelines.xhtml

European Working Book for Demographic and Socio-economic Variables. Springer Science & Business Media; 2003. 432 p.

34. Meier C, Vilpert S, Borasio GD, Maurer J, Jox RJ. Perceptions and Knowledge Regarding Medical Situations at the End of Life among Older Adults in Switzerland. J Palliat Med [Internet]. 2022 Jun 29 [cited 2022 Jul 4]; Available from: https://www.liebertpub.com/doi/10.1089/jpm.2022.0057

35. Ouellette L, Puro A, Weatherhead J, Shaheen M, Chassee T, Whalen D, et al. Public knowledge and perceptions about cardiopulmonary resuscitation (CPR): Results of a multicenter survey. Am J Emerg Med. 2018 Oct;36(10):1900–1.

36. Kerridge IH, Pearson SA, Rolfe IE, Lowe M, McPhee JR. Impact of written information on knowledge and preferences for cardiopulmonary resuscitation. Med J Aust. 1999;171(5):239–42.

37. Buiting HM, van Delden JJM, Rietjens JAC, Onwuteaka-Philipsen BD, Bilsen J, Fischer S, et al. Forgoing artificial nutrition or hydration in patients nearing death in six European countries. J Pain Symptom Manage. 2007 Sep;34(3):305–14.

38. Rietjens JAC, van der Heide A, Voogt E, Onwuteaka-Philipsen BD, van der Maas PJ, van der Wal G. Striving for quality or length at the end-of-life: attitudes of the Dutch general public. Patient Educ Couns. 2005 Nov 1;59(2):158–63.

39. Haberkern K, Schmid T, Szydlik M. Gender differences in intergenerational care in European welfare states. Ageing Soc. 2015 Feb;35(2):298–320.

40. Murphy DJ, Burrows D, Santilli S, Kemp AW, Tenner S, Kreling B, et al. The influence of the probability of survival on patients' preferences regarding cardiopulmonary resuscitation. N Engl J Med. 1994 Feb 24;330(8):545–9.

41. Sharma R, Jayathissa S, Weatherall M. Cardiopulmonary resuscitation knowledge and opinions on end of life decision making of older adults admitted to an acute medical service. N Z Med J. 2016 Jan 8;129(1428):26–36.

42. Raghupathi V, Raghupathi W. The influence of education on health: an empirical assessment of OECD countries for the period 1995–2015. Arch Public Health. 2020 Apr 6;78(1):20.

43. Vilpert S, Borrat-Besson C, Maurer J, Borasio GD. Awareness, approval and completion of advance directives in older adults in Switzerland. Swiss Med Wkly. 2018 Jul 16;148:w14642.

44. Vilpert S, Bolliger E, Borrat-Besson C, Borasio GD, Maurer J. Social, cultural and experiential patterning of attitudes and behaviour towards assisted suicide in Switzerland: evidence from a national population-based study. Swiss Med Wkly [Internet]. 2020 Jul 1 [cited 2021 Jan 4];150(2526). Available from: https://smw.ch/article/doi/smw.2020.20275

45. Mercadante S, Ferrera P, Girelli D, Casuccio A. Patients' and relatives' perceptions about intravenous and subcutaneous hydration. J Pain Symptom Manage. 2005 Oct;30(4):354–8.

46. Cotter PE, Simon M, Quinn C, O'Keeffe ST. Changing attitudes to cardiopulmonary resuscitation in older people: a 15-year follow-up study. Age Ageing. 2009 Mar 1;38(2):200–5.

47. van Gijn MS, Frijns D, van de Glind EMM, C. van Munster B, Hamaker ME. The chance of survival and the functional outcome after in-hospital cardiopulmonary resuscitation in older people: a systematic review. Age Ageing. 2014 Jul 1;43(4):456–63.

48. Piscator E, Hedberg P, Göransson K, Djärv T. Survival after in-hospital cardiac arrest is highly associated with the Age-combined Charlson Co-morbidity Index in a cohort study from a two-site Swedish University hospital. Resuscitation. 2016 Feb 1;99:79–83.

49. Chambaere K, Rietjens JA, Cohen J, Pardon K, Deschepper R, Pasman HRW, et al. Is educational attainment related to end-of-life decision-making? A large post-mortem survey in Belgium. BMC Public Health.

For peer review only

2013 Nov 9;13(1):1055.

50. Grant M, Ugalde A, Mancuso SG, Vafiadis P, Philip J. Morphine use in cancer care: A survey of attitudes and perceptions in general practice patients. Aust Fam Physician. 2017 Oct;46(10):775–80.

For peer review only

For peer review only - http://bmjopen.bmj.com/site/about/guidelines.xhtml

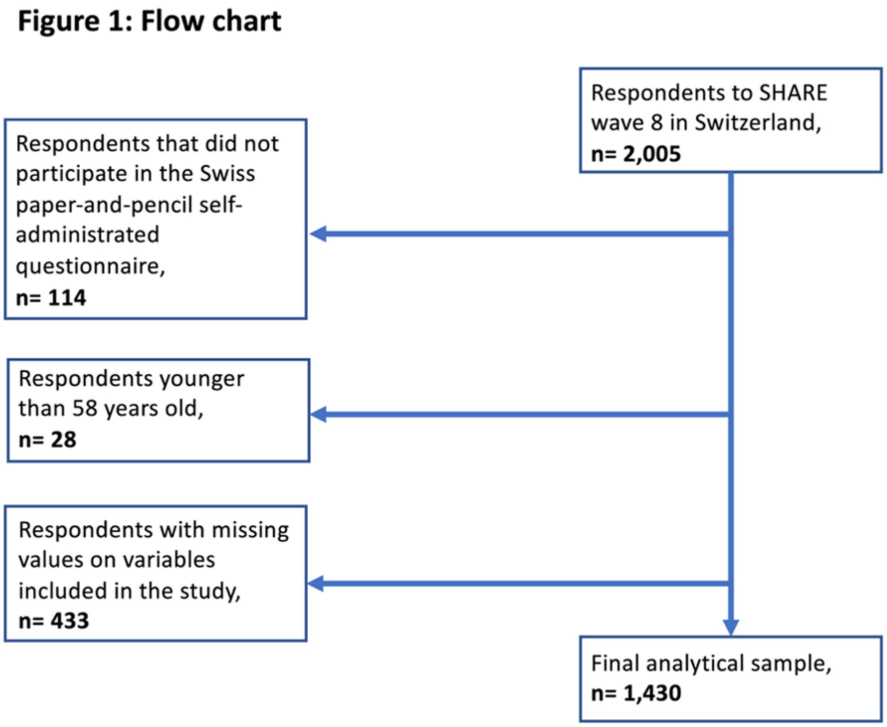

Figure 1: Flow chart

293x242mm (87 x 87 DPI)

Figure 2: Preferences for medical treatments, percentages and 95% confidence intervals, adults aged 58+, SHARE Switzerland, 2019/2020, n=1,430

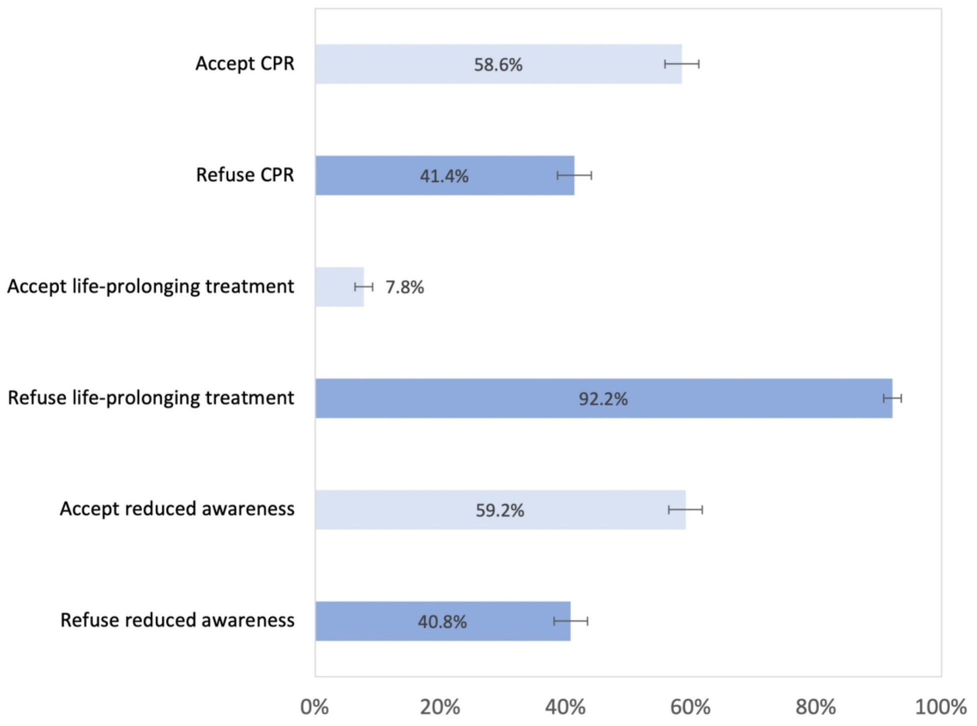

179x162mm (300 x 300 DPI)

FMH

SAMW ASSM
Schweizerische Akademie der Medizinischen Wissenschaften
Académie Suisse des Sciences Médicales
Accademia Svizzera delle Scienze Mediche
Swiss Academy of Medical Sciences

Advance directive | Detailed version

Drawn up by

Name, first name ____________________

Date of birth ____________________

Place of residence ____________________

1. This advance directive is applicable in the following situations

I am drawing up this advance directive after careful reflection in case, at some point, I am no longer able to express my wishes as a result of illness or an accident. The advance directive is to be applicable:

☐ in all situations where I am incapacitated and decisions on medical treatment have to be taken – i.e. in the case of unexpected acute events such as a heart attack, stroke or accident, but also in advanced stages of chronic disease;

or

☐ ____________________

____

2. My motivation and personal values

The following description of my motivation and personal values is a product of careful reflection and is intended to guide the health care team in the event of any difficulties of interpretation.

The following specific situation has prompted me to draw up this advance directive:

☐ (description, if appropriate) ____________________

____

☐ No specific situation; this is a precautionary measure in case I should become incapacitated.

What I particularly wish to achieve with this advance directive is…

…that all possible medical measures are taken to keep me alive. My suffering should be relieved as far as possible, but I accept that certain burdens may be associated with the preservation of my life.<br>☐ This reflects my views more closely.	or	…that medical treatment should aim above all to relieve suffering. Prolonging life at all costs is not the priority for me. I accept that my life may be shortened if certain medical treatments are withheld.<br>☐ This reflects my views more closely.

For peer review only

Describe in your own words your motivation for drawing up an advance directive:

My current situation:

My attitude to life:

My experience, attitude and fears concerning disease, dying and death:

What I understand by 'quality of life'; what impairments/losses of independence I would find it difficult to accept:

Personal and/or religious convictions by which I am guided:

☐ As a member of the following religion, I wish to receive pastoral care:

☐ I do not wish to receive pastoral care.

For peer review only

3. Statements concerning treatment goals and specific medical measures

In drawing up this advance directive, I have received advice from

☐ My GP, Dr ____________________

☐ or ____________________

☐ I do not wish to give any instructions concerning specific medical measures, but I would ask the health care team to act as far as possible in accordance with my wishes (see Section 2: 'My motivation and personal values').

☐ I wish to give specific instructions for the following situations (cf. choices I - IV).

I Unexpected acute event (e.g. accident, stroke, heart attack)

If I am incapacitated as a result of an unexpected acute event and – after initial emergency measures and careful medical assessment – it is deemed impossible or unlikely that I will regain capacity,

☐ I wish to forgo all measures which would serve merely to prolong life and suffering.

☐ I wish that, despite the poor outlook, every medically appropriate measure should be taken.

II Treatment of pain and other symptoms

☐ In any event, I wish to receive effective treatment of pain and other distressing symptoms such as anxiety, restlessness, breathing difficulties and nausea. I am prepared to accept the reduced awareness (sedation) which such treatment may induce.

or

☐ For me, alertness and the ability to communicate are more important than optimum relief of pain and other symptoms.

III Artificial hydration and nutrition

I consent to *long-term* artificial hydration and nutrition (by nasogastric tube, infusion, surgically implanted feeding tube).

☐ Yes ☐ No

If you answered no

I consent to artificial hydration and nutrition *on a temporary basis*, i.e. if it is expected that my suffering will thereby be relieved or that I will subsequently be able – possibly with support – to ingest fluids and food by the normal route again.

☐ Yes ☐ No

IV Resuscitation in the event of a cardiac and/or respiratory arrest

I wish to be resuscitated.

☐ Yes ☐ No

Additional comments:

____

____

____

____

For peer review only

4. Trusted person / healthcare proxy

☐ I have appointed the following healthcare proxy whom I authorize to make my wishes known to the health care team. This person is to be informed about my condition and involved in decision-making; he/she can access my medical records, and I release all physicians and nursing staff from their duty of confidentiality vis-à-vis this person.

Name, first name

Address

Postcode/town

Tel. home work mobile

E-mail

If my healthcare proxy cannot be contacted or is unable for other reasons to exercise this responsibility, I designate the following substitute:

Name, first name

☐ I have informed my healthcare proxy about this advance directive.

Name, first name of my general practitioner (GP)

Address

Postcode/town

Tel. home work mobile

E-mail

☐ I have informed my general practitioner (GP) about this advance directive.

5. Special instructions in the event of my death

Organ donation

☐ I wish to donate my organs, and I consent to the removal of any organs, tissue or cells and to the implementation of the preparatory medical measures which are required prior to organ donation. I consent to the use of any intensive measures this may require.

☐ I consent only to the removal of

☐ I do not wish to be a donor.

Autopsy: I consent to an autopsy

☐ Yes ☐ No ☐ I leave this decision to my healthcare proxy.

I am aware that the drawing-up of an advance directive is voluntary, and that I may revoke or modify it at any time, as long as I have mental capacity.

I have prepared separate supplements to this advance:

☐ No ☐ Yes, namely

Place, date Signature

N.B. Please keep your advance directive in such a way that it can be found should the need arise; you can leave a copy with your general practitioner. Advance directives should not be sent to the FMH/SAMS.The SAMS guidelines on advance directives are available online at *www.samw.ch*, and further information on advance directives is available at *www.fmh.ch > Patientenverfügung.*

For peer review only

FMH

SAMW ASSM
Schweizerische Akademie der Medizinischen Wissenschaften
Académie Suisse des Sciences Médicales
Accademia Svizzera delle Scienze Mediche
Swiss Academy of Medical Sciences

Advance directive | Short version

Drawn up by

Name, first name ______

Date of birth ______ Place of residence ______

If I become incapacitated, it is my wish that:

☐ all medically indicated measures (including resuscitation) should be taken to treat the acute condition so that I may regain capacity;

or

☐ I should not be resuscitated and no intensive medical interventions (in particular ventilation) should be carried out;

or

☐ I should not be resuscitated, but I consent to treatment in an intensive care unit.

If, after an initial stabilizing of my condition, it becomes clear that I am unlikely to regain capacity and that there is a high risk that I will require long-term care, it is my wish that:

☐ all life-sustaining measures continue to be carried out, provided there is still hope that I may regain capacity;

☐ no further life-sustaining measures are carried out.

☐ In any event, I wish to receive effective treatment of pain and other distressing symptoms such as anxiety, restlessness, breathing difficulties and nausea.

I have appointed the following healthcare proxy whom I authorize to make my wishes known to the health care team. This person is to be informed about my condition and involved in decision-making; he/she can access my medical records, and I release all physicians and nursing staff from their duty of confidentiality vis-à-vis this person.

Name, first name ______

Address ______ Postcode/town ______

Tel. home ______ work ______ mobile ______

E-mail ______

If my healthcare proxy cannot be contacted or is unable for other reasons to exercise this responsibility, I designate the following substitute:

Name, first name ______

☐ I have informed my healthcare proxy about this advance directive.

Name, first name of my general practitioner (GP) ______

Address ______ Postcode/town ______

Tel. home ______ work ______ mobile ______

E-mail ______

☐ I have informed my general practitioner (GP) about this advance directive.

For peer review only

(Please turn over)

Organ donation

☐ I wish to donate my organs, and I consent to the removal of any organs, tissue or cells and to the implementation of the preparatory medical measures which are required prior to organ donation. I consent to the use of any intensive measures this may require.

☐ I consent only to the removal of ______________________

☐ I do not wish to be a donor.

Place, date ______________________ Signature ______________________

For peer review only

N.B. Please keep your advance directive in such a way that it can be found should the need arise. You can leave a copy with your general practitioner. Advance directives should not be sent to the FMH/SAMS. The SAMS guidelines on advance directives are available online at *www.samw.ch*, and further information on advance directives is available at *www.fmh.ch > Patientenverfügung.*

Older adults' medical preferences for the end of life: a cross-sectional population-based survey in Switzerland

Abstract:

Objectives: Medical decision-making at the end of life is common and should be as patient-centered as possible. Our study investigates older adults' preferences toward three medical treatments that are frequently included in advance directive forms and their association with social, regional, and health characteristics.

Setting: An observational cross-sectional study using population-based data of wave 8 (2019/20) of the Swiss component of the Survey of Health, Ageing, and Retirement in Europe (SHARE).

Participants: 1,430 adults aged 58 years and older living in Switzerland.

Primary and secondary outcome measures: Three questions on the preferences regarding cardiopulmonary resuscitation (CPR); life-prolonging treatment in case of high risk of permanent mental incapacity; reduced awareness (sedation) to relieve unbearable pain and symptoms. Their associations with individuals' social, regional and health characteristics.

Results: Most older adults expressed a wish to receive CPR (58.6%) and to forgo life-prolonging treatment in case of permanent mental incapacity (90.7%). Most older adults also indicated that they would accept reduced awareness if necessary to receive effective treatment for pain and distressing symptoms (60.8%). Older adults' treatment preferences for CPR and life-prolonging treatment differed according to sex, age, partnership status, linguistic region and health status, while willingness to accept reduced awareness for effective symptom treatment was more similarly distributed across population groups.

Conclusions: Simultaneous preferences for CPR and refusal of life-prolonging treatment might appear to be conflicting treatment goals. Considering individuals' values and motivations can help clarify ambivalent treatment decisions. Structured ACP processes with trained professionals allows for exploring individuals' motivations and values and helps to identify congruent care and treatment goals.

Strengths and limitations of this study

- This study replicates the questions on treatment preferences that are usually part of advance directives.
- This study explores treatment preferences in a national population-based sample of older adults.
- The wording of the questions may not provide clear indications of individuals' actual treatment preferences.

Keywords

Advance directives; life-prolonging treatments; cardiopulmonary resuscitation; sedation; population-based study

For peer review only - http://bmjopen.bmj.com/site/about/guidelines.xhtml

Introduction:

Advances in medical technologies and extended life expectancy have changed the context of dying in today's societies (1) and often result in a need to make complex medical decisions at the end of life (EOL) (2,3). EOL decisions may concern pain or symptom management, possibly but not necessarily with life-shortening effects, withholding or withdrawing life-supporting treatment (e.g. cardiopulmonary resuscitation (CPR), artificial nutrition and hydration, mechanical ventilation, dialysis), and, in countries where it is legal, euthanasia and assisted suicide (4,5). Following the principle of patient autonomy (6), EOL decisions are jointly made by the patients and/or their surrogates and clinicians (7). These decisions ought to be based on considerations of prognosis, benefits and risks of different treatments, and patients' values and care goals among others (8). Patients commonly opt for or against certain treatments based on their perceived benefits or costs in terms of quality of life and prolongation of life, while physicians typically consider the medical appropriateness of the treatments under consideration.

EOL decisions are most often the result of multiple discussions between medical experts, patients, and potentially their families which constitute the basis of a process called advance care planning (ACP), when preparing for a patient health situation that may worsen in the future. The ACP outcomes may be recorded in advance directives (ADs). ACP aims to enable patients to receive medical treatments that are better aligned with their values, care preferences and goals (9). ACP thus contributes to maintaining patients' quality of life throughout their care episodes (10), and eventually achieve what they may have defined as a "good death" for themselves during the ACP process (11,12). ADs record the medical treatments to which a person consents or refuses. ADs can also be used to designate a healthcare proxy to make medical decisions on behalf of that person in the event of a loss of decision-making capacity (13). At the end of life, more than half of the patients lose their decision-making capacity (4). While ACP may not always entirely achieve its primary goals such as a positive impact on patients' quality of life or EOL care consistent with patient preference and goals of care, it often achieves other important secondary objectives such as improving the quality of patient-physician communication and congruence in preferences between patients and their caregivers as well as reducing decisional conflict, increasing preferences for comfort care, and encouraging the documentation of ACP and ADs (14,15). Furthermore, the existence of ADs is associated with more out-of-hospital care, an increased focus on comfort care, and may contribute to reducing overtreatment at the end of life (16).

In Switzerland, ADs have been legally binding since 2013 and individuals can find numerous AD forms published by medical associations, patient organizations or other private initiatives. Most AD forms include information on

For peer review only

For peer review only - http://bmjopen.bmj.com/site/about/guidelines.xhtml

medical and health situations in which the wishes expressed in ADs are to be applied, (individuals', treatment preferences, organ donation, and designated healthcare proxy), while some of them also contain a section about personal values, the meaning of life and quality of life and related fears and expectations (17). ADs can be completed by individuals alone, although completion with the assistance of trained professionals or healthcare specialists is generally recommended (18). While many people tend to have the impression that ADs are mainly targeted to older adults and persons with terminal illnesses, AD completion is in fact also recommended for healthy younger adults, due to the broader risks of potential loss of decision-making capacity after, for example, an accident (19).

Understanding treatment preferences commonly indicated in AD forms may be helpful to assess attitudes towards EOL medical decisions in the general population and give insights into how to best initiate discussion on the topic. Preferences regarding specific life-sustaining treatments (20–22), general EOL care goals such as priority for length or quality of life (21,23,24), and palliative sedation therapy (25–29) have been studied in the general population, but mostly in hypothetical contexts of severe physical pain or cognitive decline. To our knowledge, these preferences have not been investigated together in the way in which they might appear in AD forms. We, therefore, consider three specific questions taken from the actual AD form (see Appendix 1) used until 2022 by the Swiss Medical Association FMH, the leading physicians' association in Switzerland, which comprises 42,000 members and over 70 medical organisations in the country. In this study, we examine older adults' preferences for CPR, life-prolonging treatment, and reduced awareness (sedation) as measured by the questions from the aforementioned AD form in a large population survey of adults aged 58 years and older living in Switzerland. We also investigate the association between stated treatment preferences and individuals' social, regional, and health characteristics. This information is relevant to understanding how older adults view specific EOL treatments and identifying differences in treatment preferences among the population groups.

Methods:

We used data from the Survey of Health, Ageing and Retirement in Europe (SHARE), which collects internationally comparable multidisciplinary longitudinal microdata on social, economic and health conditions of European aged 50 and over and their partners (30). We used a subsample of the Swiss-SHARE Wave 8 (2019/2020) dataset, which included respondents who participated in the internationally harmonized in-person interview and the national paper-and-pencil self-administered EOL questionnaire distributed at the end of the interview in Switzerland (31,32). The EOL questionnaire was developed by the Swiss SHARE team and palliative

For peer review only - http://bmjopen.bmj.com/site/about/guidelines.xhtml

For peer review only

care experts of the Lausanne University Hospital and concerns preferences, knowledge, attitudes, and behaviors toward EOL care planning (32). In Wave 8 in Switzerland, 2,005 respondents participated in the SHARE main interview and 94.3% of them (1,891 individuals) also completed the EOL questionnaire. Since the last sample refreshment in Switzerland was in 2011, only 28 respondents to the Swiss-SHARE Wave 8 survey, which had entered the study as partners, were aged 50-57. We, therefore, discard these respondents from our sample and limit our analyses to respondents aged 58 years and older who had no missing values on any of the items used in our analysis. Our selected final analytical sample comprises 1,430 respondents (Figure1).

(Insert figure 1 here)

Measures

Outcome variables:

We included three questions in our EOL questionnaire, which have been adapted directly from the long version of the AD form that was publicly available for download from the Swiss Medical Association FMH website. The adaptations made for inclusion in our survey mainly consisted of transforming the statements into questions, while keeping the vocabulary and contextual information provided as in the Swiss Medical Association's AD forms.

Question 1. Imagine that you experience a cardiac and/or respiratory arrest. In this situation, you wish:

- ☐ to be resuscitated (1=accept CPR).
- ☐ not to be resuscitated (0=refuse CPR)

Question 2. Imagine that you are incapacitated following an accident, a stroke, or a heart attack. After initial emergency measures and careful medical assessment, physicians deem it very unlikely that you will regain mental capacity. In this situation, you prefer:

- ☐ to forgo all measures which would only serve to prolong your life and suffering (0=refuse life-prolonging treatment)
- ☐ that, despite the poor outlook, every medically appropriate measure should be taken ((1=accept life-prolonging treatment).

Question 3. Imagine that you suffer from a disease that causes unbearable pain and symptoms such as fear, restlessness, breathing difficulties, and nausea. In this situation:

For peer review only - http://bmjopen.bmj.com/site/about/guidelines.xhtml

- you wish to receive optimal treatment of pain and other distressing symptoms, and you are prepared to accept the reduced awareness (sedation) that such treatment may induce (1=accept reduced awareness)
- for you, alertness and the ability to communicate are more important than optimal relief of pain and other symptoms (0=refuse reduced awareness).

Independent variables:

We considered social, regional and health individuals' characteristics as independent variables in our study. Social characteristics included information on sex (0=male, 1=female), age group (58-64 years, 65-74 years, 75+ years), education level based on the International Standard Classification of Education (ISCED) of 2017 (33) (basic=ISCED levels 0-1-2, secondary=ISCED levels 3-4, tertiary=ISCED levels 5-6), living with a partner (0=has a partner, 1=has no partner), parenthood (0=no children, 1=at least one child) and self-perceived financial difficulties (Is your household able to make ends meet?: 1=easily, 2=fairly easily, 3=with difficulty). Regional characteristics comprised variables on the living environment (0=urban, 1=rural), language regions (German, French or Italian) and religiosity (0=never pray, 1=pray at least sometimes). Health characteristics involved two variables: self-rated health (1 = poor/fair health, 2 = good health, 3 = very good/excellent health) and self-reported limitation in activities of daily living (ADLs) (0=no limitation, 1=at least one limitation).

Statistical analysis:

We performed proportion estimation for all the variables included in the study. We further used probit regressions to assess the partial associations of preferences for medical treatments with respondents' characteristics. Regression estimates are expressed in terms of average partial effects (APEs). APEs report (in percentage points) how the average conditional outcome probability P(Y=1 | X) changes when the given explanatory variable changes from 0 to 1, holding all other independent variables at their observed values. Estimated standard errors were clustered at the household level to account for potential unobserved dependencies between partners' responses. All estimations were conducted using STATA/SE 17.0 software (STATA Corporation, College Station, TX).

Ethical approval

The study was approved by the research ethics committee of the canton of Vaud in March 2014 with number 66/14. In addition, during data collection, respondents gave their consent to participate in the survey on two occasions, when they agreed over the phone to schedule an interview and at the beginning of the in-person interview.

For peer review only

For peer review only - http://bmjopen.bmj.com/site/about/guidelines.xhtml

Patient and Public Involvement

Patients and the public were not directly involved in the design, conduct, reporting or dissemination of this study as it uses SHARE data (secondary data) and direct public contribution was therefore not possibletheir contribution would not have been relevant to the research question.

Results:

The social, cultural, and health characteristics of the study population are presented in Table 1. Regarding preferences for medical treatments displayed in Figure 2, 58.6% of respondents preferred to receive CPR, 90.7% chose to forgo life-prolonging treatment in case of high risk of permanent mental incapacity, and 60.8% accepted reduced awareness for optimum treatment of pain and other distressing symptoms.

For peer review only

For peer review only - http://bmjopen.bmj.com/site/about/guidelines.xhtml

Table 1: Characteristics of the study sample, adults aged 58+, SHARE Switzerland, 2019/2020, n=1,430		
	Number	Proportions (%)
Gender		
Male	676	47.3
Female	754	52.7
Age groups		
58-64 years	351	24.6
65-74 years	603	42.2
75+ years	476	33.3
Education		
Basic	231	16.2
Secondary	911	63.7
Tertiary	288	20.1
Living with a partner		
Yes	1,071	74.9
No	359	25.1
Parenthood		
No children	221	15.5
At least on child	1,209	84.5
Make ends meet		
Easily	798	55.8
Fairly easily	457	32
With difficulty	175	12.2
Living environment		
Urban	657	45.9
Rural	773	54.1
Switzerland linguistic regions		
German	1,037	72.5
French	345	24.1
Italian	48	3.4
Religiosity		
Never pray	441	30.8
Pray at least sometimes	989	69.2
Self-rated health		
Poor/fair health	250	17.5
Good health	607	42.4
Very good/excellent health	573	40.1
Self-reported limitations in activities of daily living (ADLs)		
No limitation	1,343	93.9
At least one limitation	87	6.1

(Insert Figure 2 here)

For peer review only

Table 2 shows the results from the multivariable probit regressions of preferences for the three different medical treatments on respondents' characteristics. Concerning preferences for CPR, being a woman decreases the probability of wanting to be resuscitated by 11.2 percentage points compared to being a man (APE: -11.2, $p < 0.001$). In addition, living without a partner (APE: -8.2; $p < 0.05$) and being older than the reference age group of 58-64 (APE 65-74: -10.1, $p < 0.01$; APE 75+: -20.4, $p < 0.001$) were also each associated with a lower likelihood of wanting to be resuscitated. In contrast, having a tertiary level of education (APE: 10.3, $p < 0.05$), being from the Italian-speaking part of Switzerland (APE: 24.8, $p < 0.001$), praying at least sometimes (APE: 5.6, $p < 0.01$), and self-rating one's health as good (APE: 9.4, $p < 0.05$) or very good/excellent (APE: 12.0, $p < 0.01$) were positively associated with preferences for CPR.

Regarding general preferences for life-prolonging treatment, being a woman (APE: -4.3, $p < 0.01$), being 65- to 74 years old (APE: -3.8, $p < 0.05$), and having at least one limitation in activities of daily living (APE: -4.8, $p < 0.05$) were all negatively associated with preference for life-prolonging treatment in case of high risk of permanent mental incapacity. On the other side, living without a partner (APE: 4.3, $p < 0.01$) and being from the Italian-speaking part of Switzerland (APE: 17.7, $p < 0.01$) were associated with a higher chance of wanting life-prolonging treatment.

Concerning acceptance of reduced awareness, having a secondary level of education (APE: 8.9, $p < 0.01$) was associated with a higher likelihood of accepting reduced awareness for optimum pain treatment and other distressing symptoms. At the same time, respondents who said they pray at least sometimes (APE: -6.2, $p < 0.01$) were less likely to accept reduced awareness in order to receive optimal treatment of pain and other distressing symptoms.

For peer review only - http://bmjopen.bmj.com/site/about/guidelines.xhtml

**Table 2: Partial associations of preferences for medical treatments with respondents' social, cultural, and health characteristics, adults aged 58+, SHARE Switzerland, 2019/2020, n=1,430**			
	Preference for CPR APE (95% CI)	Preference for life-prolonging treatment APE (95% CI)	Preference for reduced awareness APE (95% CI)
Gender (ref: male)			
Female	-11.2*** (-16.2,-6.3)	-4.3** (-7.3,-1.4)	2.3 (-2.9,7.4)
Age groups (ref: 58-64 years)			
65-74 years	-10.1** (-16.3,-4.0)	-3.8* (-7.6,-0.1)	2.2 (-4.2,8.5)
75+ years	-20.4*** (-27.3,-13.5)	-4.0 (-8.0,0.0)	-5.1 (-12.4,2.2)
Education (ref: basic)			
Secondary	1.2 (-6.0,8.4)	-4.1 (-8.8,0.7)	8.9* (1.4,16.3)
Tertiary	10.3* (1.5,19.1)	-4.1 (-9.5,1.4)	7.6 (-1.8,16.9)
Living with a partner (ref: yes)			
No	-8.2* (-14.4,-1.9)	4.3* (0.5,8.1)	2.2 (-4.1,8.5)
Parenthood (ref: no children)			
At least on child	0.6 (-6.5,7.7)	-0.6 (-4.5,3.2)	3.3 (-3.9,10.5)
Ease of making ends meet (ref: easy)			
Fairly easily	1.6 (-4.2,7.4)	1.1 (-2.0,4.3)	-2.4 (-8.4,3.7)
With difficulty	2.2 (-6.0,10.3)	2.9 (-2.2,8.1)	-0.0 (-8.7,8.6)
Living environment (ref: urban)			
Rural	1.4 (-3.8,6.6)	0.3 (-2.5,3.0)	-0.0 (-5.4,5.3)
Switzerland linguistic regions (ref: German)			
French	-6.1 (-12.3,0.0)	2.3 (-1.1,5.7)	2.6 (-3.5,8.7)
Italian	24.8*** (15.2,34.3)	17.7* (4.0,31.4)	-7.5 (-23.5,8.5)
Religiosity (ref: never pray)			
Pray at least sometimes	5.6* (0.1,11.1)	0.5 (-2.5,3.5)	-6.2* (-11.8,-0.5)
Self-rated health (ref: poor/fair health)			
Good health	9.4* (1.8,16.9)	-2.4 (-6.6,1.8)	2.6 (-4.8,10.0)
Very good/excellent health	12.0** (4.0,20.0)	-1.9 (-6.3,2.5)	-3.3 (-11.4,4.7)
Self-reported limitations in activities of daily living (ADLs) (ref: no limitation)			
At least one limitation	-1.7 (-12.7,9.4)	-4.8* (-8.6,-0.9)	0.9 (-10.4,12.3)
Observations	1,430	1,430	1,430

*CPR=Cardiopulmonary resuscitation. Average partial effects (APEs) based on probit regression models. All probabilities are multiplied by 100 to express APEs in percentage points. Asterisks indicate levels of significance: ***<0.1%, ** 1%, *5%. Interpretation of APEs: Being a woman decreases the probability of wanting to be resuscitated by 11.2 percentage points compared to being a man. For variables with three response categories the interpretation is as follows. Being aged 75 and over reduces the probability of wanting to be resuscitated by 20.4 percentage points in comparison with being aged 58-64 (reference category).*

For peer review only - http://bmjopen.bmj.com/site/about/guidelines.xhtml

For peer review only

Discussion

Our study is the first national population-based study in Switzerland that investigates older adults' preferences toward specific medical treatments that are commonly elicited in official AD forms. Most respondents wished to receive CPR in case of a cardiac/respiratory arrest, to forgo life-prolonging treatment in case of high risk of permanent mental incapacity, and to reduce their awareness for optimal treatment of pain and other distressing symptoms. Additionally, significant social, regional, and health-related differences in medical treatment preferences were found for CPR and life-sustaining treatments, while preferences for reduced awareness were more uniformly distributed across different population groups.

Preference for cardiopulmonary resuscitation

A majority of individuals reported wanting to receive CPR in the event of cardiac and/or respiratory arrest. In most population-based studies investigating preferences for CPR, the question is asked for a given health condition (e.g., terminal cancer, advanced dementia), and individuals largely refuse CPR (20,21,25). There are several reasons that could explain this difference between our study and previous ones. In our study, the context of the CPR is not specified, and respondents may be referring to their current health status, which is generally rather good (84.8% declared a good, very good, or excellent health) with the preference for CPR being potentially influenced by the prospect of significant remaining longevity (24). Alternatively, this difference could also be explained by an overestimation of CPR survival rate (34), which may lead individuals to choose CPR (35). Some individuals may decide against CPR once they have been informed of its procedure, risks and benefits in different contexts (36).

Regarding social differences in CPR preferences in our study, women reported lower preferences for CPR than men. This finding, which has been shown in other studies (21,25), could be explained by a higher valuation of quality of life over length of life among women compared to men (37) which may lead them to refrain from treatments (38). Moreover, women are more likely to have direct experience with caregiving (39) that may make them more aware of the benefits and limitations of certain medical treatments (34) and the potential burden that these treatments may imply for patients and their relatives (24). Respondents living without a partner were also less likely to opt for CPR than respondents living with a partner. Since respondents living without a partner also seem to be better informed about the limited success of CPR, as shown in a previous study by the authors based on the same Swiss subsample of SHARE Wave 8 (34), they may prefer to refuse CPR (40).

For peer review only - http://bmjopen.bmj.com/site/about/guidelines.xhtml

Conversely, respondents with a tertiary level of education were more likely to report a preference for CPR. Our previous study (34) showed that older adults with higher levels of education were not more knowledgeable about the (relatively low) chances of success of CPR and may, therefore, not be more likely to forgo CPR than persons with less education (34). A study of hospitalized patients aged 65 and over in New Zealand reported that more educated patients were more likely to prefer CPR in their current state of health (41). One potential reason could be that individuals with higher education believe to have better health prospects (42) which could induce them to opt for CPR. One study showed that older adults who are optimistic about their own life expectancy are more likely to want to continue all medical treatment in a situation of serious illness even if there are very low chances of survival (24). Acceptance of CPR was also higher in the Italian linguistic region and among more religious people, who report to pray at least sometimes. These findings may highlight well-documented cultural differences in Switzerland, with respondents from the Italian-speaking part (43,44) and more religious persons being more likely to be influenced by religious considerations and corresponding beliefs in the sanctity of life (38,44,45) that tend to encourage life-prolonging treatments (11).

Finally, age and self-rated health status, which are two parameters of medical prognoses, were also strongly associated with CPR preferences. Compared to adults aged 58-64, older respondents were less likely to state that they would prefer to receive CPR, with this negative association increasing in age. These results are consistent with other studies showing that older people are more likely to refuse CPR (21,25,46). Older adults might realize that age decreases their chances of surviving a CPR (47) and, therefore, be more likely to reject this treatment. The same reasoning may also apply to people in poorer health (48).

Preference for life-prolonging treatment

Over 90% of our respondents indicated a preference to forgo life-prolonging treatment if they were to become incapacitated following an accident, a stroke, or a heart attack and were very unlikely to regain mental capacity. Similar results were found in studies of preferences to continue or stop treatment in the presence of serious physical or mental illness in the general population (21,24). Interestingly, the population groups more likely to forgo life-prolonging treatments are comparable to those who refused CPR, that is, women, older persons, and those in poorer health (1+ ADLs). These findings are in line with a study of the general Dutch population that showed that women, older people, and people with a history of serious illness tend to prioritize quality of life over quantity (38). In contrast, Swiss participants from the Italian-speaking region who were more likely to prefer CPR were also more likely to prefer life-prolonging treatment. A surprising result for which we have no explanation is the preference

For peer review only - http://bmjopen.bmj.com/site/about/guidelines.xhtml

for life-sustaining treatments among people living without a partner, especially since many of them simultaneously indicated to refuse CPR.

Preference for reduced awareness

In our study, 60.8% of our respondents accepted reduced awareness in a situation where they would suffer from a disease that causes unbearable pain and symptoms such as fear, restlessness, breathing difficulties, and nausea, while for the remaining respondents (39.2%), awareness and the ability to communicate was more important than optimal relief of pain and other symptoms. Other studies of individuals' preferences for different types of palliative sedation (e.g. intermittent, mild, continuous deep sedation), in different situations and for different durations have produced mixed results, which are not directly comparable to ours (25–29). Interestingly, although adequate pain and symptoms management is one of the most important features of a good death (28), preservation of consciousness also plays a central role at the end of life for many people (11,25,27). In a Dutch study, 61% of adults aged 20+ reported being conscious until death as important. Those individuals were less likely to accept terminal sedation (67%) and the use of high dosages of morphine (67%) (28). These findings highlight the clinical importance to clearly discuss the possible interaction and trade-offs between symptom alleviation and decreased alertness with patients.

Only education and religiosity were associated with individuals' preferences for or against reduced awareness as a side effect of optimal symptom treatment. People with secondary education are more likely to accept reduced awareness, which is consistent with a Belgian study that showed that patients with higher levels of education are more likely to emphasize pain and symptom relief at the end of life (49). Furthermore, more religious respondents were less likely to accept reduced awareness. Another study pointed that the proportion of individuals rejecting gradual and rapid sedation was higher among more religious people (29). These associations may be linked to the belief that symptom treatment that reduces awareness also shortens life (50), especially since religiosity is commonly associated with life-prolonging treatment preferences among older adults (21,24,38).

Potential discrepancy in treatment preferences

Our results may at first seem contradictory in that most respondents indicated a preference against life-prolonging treatments but at the same time showed a preference for CPR, which has a relatively low chance of survival. This type of ambivalence has already been noted in a study that showed that about one-third of the Dutch adult population could not make a clear choice between quality and quantity of life (38), which could be compared in our study with the refusal of EOL treatments (quality of life) and the simultaneous preference for resuscitation

For peer review only - http://bmjopen.bmj.com/site/about/guidelines.xhtml

(quantity of life). Another study also pointed to inconsistencies between Dutch older adults' general EOL goals and preferences for specific life-sustaining treatments (20). Their authors explained these results by the lack of knowledge of the risks and side effects of these treatments in the population.

The apparent contradiction between preferences for CPR and life-prolonging treatments in our study may be related to the framing of our questions. Our CPR question provides no information about the context in which the CPR is to be conducted such as whether the CPR would take place in- or out-hospital, and individual's health status is also not specified in this question. By contrast, the question on life-prolonging treatment refers to a situation of very bad health which may explain why the overwhelming majority of respondents reported a preference to refuse life-prolonging treatment in this context. Both questions were directly drawn from the Swiss Medical Association's AD form, which was in use at the time of our survey, but has been revised since. The long version of this form contains supplementary contextual information on individuals' motivations, health history and social situation, which seems helpful in translating patients' values and preferences into medically actionable information. Nevertheless, this supplementary information may not completely prevent potential conflicting information about individuals' treatment preferences in case of patients' loss of decision capacity, especially if this information may stand in contrast to individuals' treatment wishes as indicated by the check box questions. A broader approach to ACP that can be translated into more nuanced ADs is clearly desirable and increasingly implemented in practice (10).

Limitations

One limitation of our study is that our question wording may not be ideal for eliciting people's treatment preferences even if the questions were highly relevant to Swiss EOL policy at the time of our survey due to their close correspondence with a widely known AD form in Switzerland. Another limitation is that people in very poor health or living in a nursing home may be underrepresented in our study, despite the considerable efforts of SHARE to interview also sicker people and follow respondents into institutions such as nursing homes. On the other hand, the EOL questionnaire's cooperation rate was high (94.3%), and an analysis of the profile of non-respondents based on individuals' characteristics used in our regression models indicated no obvious underrepresentation of certain population groups.

For peer review only - http://bmjopen.bmj.com/site/about/guidelines.xhtml

Conclusion

Our study investigates preferences for CPR, life-prolonging treatments, and reduced awareness in the general population of adults aged 58 years and older living in Switzerland based on three statements included in the FMH Swiss Medical Association's AD form. Most older adults wished to receive CPR in case of a cardiac/respiratory arrest, forgo life-prolonging treatment in case of high risk of permanent mental incapacity, and reduce their awareness for optimal treatment of pain and other distressing symptoms. Our study also revealed that individuals' preferences appear to be influenced by characteristics that affect medical prognoses, such as advanced age and poor health. Moreover, women were less likely to report a preference for CPR and life-prolonging treatments than men. Furthermore, the apparent contradiction in our findings between individuals' willingness to receive CPR and their wishes to forgo life-prolonging treatment may reveal knowledge gaps regarding the success chances and side effects of certain treatments and also highlights the potential ambivalence between individuals' EOL care goals and the choice they make for specific EOL treatments. This inherent complexity of making advance decisions in the context of ADs could benefit from further contextual information and elicitation of individuals' values through processes of ACP, which facilitate a clearer understanding of individuals' values, motivations and fears concerning EOL care that can help to better translate ADs and related information into medical decisions at the EOL.

Contributorship statement

SV designed the study. CM and JB conducted the statistical analysis with the support of SV. SV, CM and JM drafted the manuscript. All authors discussed the interpretation of findings and provided critical revision of the manuscript for important intellectual content.

Competing interests

The authors declare that they have no competing interests.

Funding

This work was supported by the Swiss National Science Foundation (SNSF), grant number 10001C_188836.

This paper uses data from Börsch-Supan, A. (2021). Survey of Health, Ageing and Retirement in Europe (SHARE) Wave 8. Release version: 1.0.0. SHARE-ERIC. Data set. DOI: 10.6103/SHARE.w8.100. The SHARE data collection has been funded by the European Commission, DG RTD through FP5 (QLK6-CT-2001-00360), FP6

For peer review only - http://bmjopen.bmj.com/site/about/guidelines.xhtml

For peer review only

(SHARE-I3: RII-CT-2006-062193, COMPARE: CIT5-CT-2005-028857, SHARELIFE: CIT4-CT-2006-028812), FP7 (SHARE-PREP: GA N°211909, SHARE-LEAP: GA N°227822, SHARE M4: GAN°261982, DASISH: GA N°283646) and Horizon 2020 (SHARE-DEV3: GA N°676536, SHARE-COHESION: GA N°870628, SERISS: GA N°654221, SSHOC: GA N°823782) and by DG Employment, Social Affairs & Inclusion through VS 2015/0195, VS 2016/0135, VS 2018/0285, VS 2019/0332, and VS 2020/0313. Additional funding from the German Ministry of Education and Research, the Max Planck Society for the Advancement of Science, the U.S. National Institute on Aging (U01_AG09740-13S2, P01_AG005842, P01_AG08291, P30_AG12815, R21_AG025169, Y1-AG-4553-01, IAG_BSR06-11, OGHA_04-064, HHSN271201300071C, RAG052527A) and from various national funding sources is gratefully acknowledged (see https://share-eric.eu/).

Data sharing statement

SHARE data are publicly available for scientific purposes only through registration on the data distribution platform.

Ethics Approval

The study was approved by the research ethics committee of the canton of Vaud in March 2014 with number 66/14.

For peer review only

References

1. Etkind SN, Bone AE, Gomes B, Lovell N, Evans CJ, Higginson IJ, et al. How many people will need palliative care in 2040? Past trends, future projections and implications for services. BMC Med. 2017 May 18;15(1):102.

2. Bosshard G, Zellweger U, Bopp M, Schmid M, Hurst SA, Puhan MA, et al. Medical End-of-Life Practices in Switzerland: A Comparison of 2001 and 2013. JAMA Intern Med. 2016 Apr;176(4):555–6.

3. Becker C, Künzli N, Perrig S, Beck K, Vincent A, Widmer M, et al. Code status discussions in medical inpatients: results of a survey of patients and physicians. Swiss Med Wkly. 2020 Mar 23;150(1314).

4. van der Heide A, Deliens L, Faisst K, Nilstun T, Norup M, Paci E, et al. End-of-life decision-making in six European countries: descriptive study. The Lancet. 2003 Aug 2;362(9381):345–50.

5. Löfmark R, Nilstun T, Cartwright C, Fischer S, van der Heide A, Mortier F, et al. Physicians' experiences with end-of-life decision-making: Survey in 6 European countries and Australia. BMC Med. 2008 Feb 12;6(1):4.

6. Principles of Biomedical Ethics - Paperback - Tom L. Beauchamp; James F. Childress - Oxford University Press [Internet]. [cited 2022 Nov 16]. Available from: //global.oup.com/ushe/product/principles-of-biomedical-ethics-9780190640873

7. Rosca A, Krones T, Biller-Andorno N. Shared decision making: patients have a right to be informed about possible treatment options and their risks and benefits. Swiss Med Wkly. 2020 Jul 6;150(2728):w20268–w20268.

8. Mehta RS, Anderson WG, Hunt S, Chaitin EK, Arnold RM. Chapter 22 - Withholding and Withdrawing Life-Sustaining Therapies. In: Emanuel LL, Librach SL, editors. Palliative Care (Second Edition) [Internet]. Saint Louis: W.B. Saunders; 2011 [cited 2023 Apr 25]. p. 300–18. Available from: https://www.sciencedirect.com/science/article/pii/B9781437716191000226

9. Sudore RL, Lum HD, You JJ, Hanson LC, Meier DE, Pantilat SZ, et al. Defining Advance Care Planning for Adults: A Consensus Definition From a Multidisciplinary Delphi Panel. J Pain Symptom Manage. 2017 May;53(5):821-832.e1.

10. Rietjens JAC, Sudore RL, Connolly M, van Delden JJ, Drickamer MA, Droger M, et al. Definition and recommendations for advance care planning: an international consensus supported by the European Association for Palliative Care. Lancet Oncol. 2017 Sep;18(9):e543–51.

11. Borrat-Besson C, Vilpert S, Borasio GD, Maurer J. Views on a "Good Death": End-of-Life Preferences and Their Association With Socio-Demographic Characteristics in a Representative Sample of Older Adults in Switzerland. OMEGA - J Death Dying. 2020 Jul 29;

12. Krikorian A, Maldonado C, Pastrana T. Patient's Perspectives on the Notion of a Good Death: A Systematic Review of the Literature. J Pain Symptom Manage. 2020 Jan;59(1):152–64.

13. Pautex S, Herrmann F, Zulian G. Role of advance directives in palliative care units: a prospective study. Palliat Med. 2008 Oct 1;22(7):835–41.

14. Malhotra C, Shafiq M, Batcagan-Abueg APM. What is the evidence for efficacy of advance care planning in improving patient outcomes? A systematic review of randomised controlled trials. BMJ Open. 2022 Jul 1;12(7):e060201.

15. Jimenez G, Tan WS, Virk AK, Low CK, Car J, Ho AHY. Overview of Systematic Reviews of Advance Care Planning: Summary of Evidence and Global Lessons. J Pain Symptom Manage. 2018 Sep;56(3):436-459.e25.

16. Brinkman-Stoppelenburg A, Rietjens JA, van der Heide A. The effects of advance care planning on end-of-life care: A systematic review. Palliat Med. 2014 Sep 1;28(8):1000–25.

For peer review only - http://bmjopen.bmj.com/site/about/guidelines.xhtml

17. Rüegger H. Patientenverfügungen in der deutschsprachigen Schweiz, Institut Neumünster, CURAVIVA. 2011;45.

18. Medical-ethical Guidelines [Internet]. [cited 2023 Apr 24]. Medical-ethical Guidelines. Available from: https://www.samw.ch

19. Otte I, Jung C, Elger B, Bally K. Advance directives and the impact of timing. A qualitative study with Swiss general practitioners. Swiss Med Wkly [Internet]. 2014 Sep 21 [cited 2022 Apr 1];(39). Available from: https://smw.ch/article/doi/smw.2014.14035

20. Evans N, Pasman HR, Deeg D, Onwuteaka-Philipsen B, EURO IMPACT. How do general end-of-life treatment goals and values relate to specific treatment preferences? a population-based study. Palliat Med. 2014 Dec;28(10):1206–12.

21. van Wijmen MPS, Pasman HRW, Widdershoven GAM, Onwuteaka-Philipsen BD. Continuing or forgoing treatment at the end of life? Preferences of the general public and people with an advance directive. J Med Ethics. 2015 Aug;41(8):599–606.

22. Hamano J, Hanari K, Tamiya N. End-of-life care preferences of the general public and recommendations of healthcare providers: a nationwide survey in Japan. BMC Palliat Care. 2020 Mar 24;19(1):38.

23. Chung RYN, Wong ELY, Kiang N, Chau PYK, Lau JYC, Wong SYS, et al. Knowledge, Attitudes, and Preferences of Advance Decisions, End-of-Life Care, and Place of Care and Death in Hong Kong. A Population-Based Telephone Survey of 1067 Adults. J Am Med Dir Assoc. 2017 Apr 1;18(4):367.e19-367.e27.

24. Carr D, Moorman SM. End-of-Life Treatment Preferences Among Older Adults: An Assessment of Psychosocial Influences. Sociol Forum Randolph NJ. 2009 Dec 1;24(4):754–78.

25. Sanjo M, Miyashita M, Morita T, Hirai K, Kawa M, Akechi T, et al. Preferences regarding end-of-life cancer care and associations with good-death concepts: a population-based survey in Japan. Ann Oncol Off J Eur Soc Med Oncol. 2007 Sep;18(9):1539–47.

26. Bérubé A, Tapp D, Dupéré S, Plaisance A, Bravo G, Downar J, et al. Do Socioeconomic Factors Influence Knowledge, Attitudes, and Representations of End-of-Life Practices? A Cross-Sectional Study. J Palliat Care. 2022 Oct 13;8258597221131658.

27. Morita T, Hirai K, Okazaki Y. Preferences for Palliative Sedation Therapy in the Japanese General Population. J Palliat Med. 2002 Jun;5(3):375–85.

28. Rietjens JA, van der Heide A, Onwuteaka-Philipsen BD, van der Maas PJ, van der Wal G. Preferences of the Dutch general public for a good death and associations with attitudes towards end-of-life decision-making. Palliat Med. 2006 Oct 1;20(7):685–92.

29. Takla A, Savulescu J, Kappes A, Wilkinson DJC. British laypeople's attitudes towards gradual sedation, sedation to unconsciousness and euthanasia at the end of life. PLOS ONE. 2021 Mar 26;16(3):e0247193.

30. Börsch-Supan A, Brandt M, Hunkler C, Kneip T, Korbmacher J, Malter F, et al. Data Resource Profile: The Survey of Health, Ageing and Retirement in Europe (SHARE). Int J Epidemiol. 2013 Aug 1;42(4):992–1001.

31. [dataset] Börsch-Supan, A. Data from: Survey of Health, Ageing and Retirement in Europe (SHARE) Wave 8. Release version: 8.0.0. SHARE-ERIC, 10th February 2022. DOI: 10.6103/SHARE.w8.800. ~~Börsch-Supan A. Survey of Health, Ageing and Retirement in Europe (SHARE) Wave 8 [Internet]. SHARE-ERIC; 2022 [cited 2023 Feb 6]. Available from: http://www.share-project.org/data-documentation/waves-overview/wave-8.html~~

32. Bergmann M, Börsch-Supan A (Eds.) (2021). SHARE Wave 8 Methodology: Collecting Cross-

For peer review only

For peer review only - http://bmjopen.bmj.com/site/about/guidelines.xhtml

National Survey Data in Times of COVID-19. Munich MEA Max Planck Inst Soc Law Soc Policy.

33. Hoffmeyer-Zlotnik JHP, Hoffmeyer-Zlotnik JHP, Wolf C. Advances in Cross-National Comparison: A European Working Book for Demographic and Socio-economic Variables. Springer Science & Business Media; 2003. 432 p.

34. Meier C, Vilpert S, Borasio GD, Maurer J, Jox RJ. Perceptions and Knowledge Regarding Medical Situations at the End of Life among Older Adults in Switzerland. J Palliat Med [Internet]. 2022 Jun 29 [cited 2022 Jul 4]; Available from: https://www.liebertpub.com/doi/10.1089/jpm.2022.0057

35. Ouellette L, Puro A, Weatherhead J, Shaheen M, Chassee T, Whalen D, et al. Public knowledge and perceptions about cardiopulmonary resuscitation (CPR): Results of a multicenter survey. Am J Emerg Med. 2018 Oct;36(10):1900–1.

36. Kerridge IH, Pearson SA, Rolfe IE, Lowe M, McPhee JR. Impact of written information on knowledge and preferences for cardiopulmonary resuscitation. Med J Aust. 1999;171(5):239–42.

37. Buiting HM, van Delden JJM, Rietjens JAC, Onwuteaka-Philipsen BD, Bilsen J, Fischer S, et al. Forgoing artificial nutrition or hydration in patients nearing death in six European countries. J Pain Symptom Manage. 2007 Sep;34(3):305–14.

38. Rietjens JAC, van der Heide A, Voogt E, Onwuteaka-Philipsen BD, van der Maas PJ, van der Wal G. Striving for quality or length at the end-of-life: attitudes of the Dutch general public. Patient Educ Couns. 2005 Nov 1;59(2):158–63.

39. Haberkern K, Schmid T, Szydlik M. Gender differences in intergenerational care in European welfare states. Ageing Soc. 2015 Feb;35(2):298–320.

40. Murphy DJ, Burrows D, Santilli S, Kemp AW, Tenner S, Kreling B, et al. The influence of the probability of survival on patients' preferences regarding cardiopulmonary resuscitation. N Engl J Med. 1994 Feb 24;330(8):545–9.

41. Sharma R, Jayathissa S, Weatherall M. Cardiopulmonary resuscitation knowledge and opinions on end of life decision making of older adults admitted to an acute medical service. N Z Med J. 2016 Jan 8;129(1428):26–36.

42. Raghupathi V, Raghupathi W. The influence of education on health: an empirical assessment of OECD countries for the period 1995–2015. Arch Public Health. 2020 Apr 6;78(1):20.

43. Vilpert S, Borrat-Besson C, Maurer J, Borasio GD. Awareness, approval and completion of advance directives in older adults in Switzerland. Swiss Med Wkly. 2018 Jul 16;148:w14642.

44. Vilpert S, Bolliger E, Borrat-Besson C, Borasio GD, Maurer J. Social, cultural and experiential patterning of attitudes and behaviour towards assisted suicide in Switzerland: evidence from a national population-based study. Swiss Med Wkly [Internet]. 2020 Jul 1 [cited 2021 Jan 4];150(2526). Available from: https://smw.ch/article/doi/smw.2020.20275

45. Mercadante S, Ferrera P, Girelli D, Casuccio A. Patients' and relatives' perceptions about intravenous and subcutaneous hydration. J Pain Symptom Manage. 2005 Oct;30(4):354–8.

46. Cotter PE, Simon M, Quinn C, O'Keeffe ST. Changing attitudes to cardiopulmonary resuscitation in older people: a 15-year follow-up study. Age Ageing. 2009 Mar 1;38(2):200–5.

47. van Gijn MS, Frijns D, van de Glind EMM, C. van Munster B, Hamaker ME. The chance of survival and the functional outcome after in-hospital cardiopulmonary resuscitation in older people: a systematic review. Age Ageing. 2014 Jul 1;43(4):456–63.

48. Piscator E, Hedberg P, Göransson K, Djärv T. Survival after in-hospital cardiac arrest is highly associated with the Age-combined Charlson Co-morbidity Index in a cohort study from a two-site Swedish University hospital. Resuscitation. 2016 Feb 1;99:79–83.

For peer review only - http://bmjopen.bmj.com/site/about/guidelines.xhtml

49. Chambaere K, Rietjens JA, Cohen J, Pardon K, Deschepper R, Pasman HRW, et al. Is educational attainment related to end-of-life decision-making? A large post-mortem survey in Belgium. BMC Public Health. 2013 Nov 9;13(1):1055.

50. Grant M, Ugalde A, Mancuso SG, Vafiadis P, Philip J. Morphine use in cancer care: A survey of attitudes and perceptions in general practice patients. Aust Fam Physician. 2017 Oct;46(10):775–80.

For peer review only

For peer review only - http://bmjopen.bmj.com/site/about/guidelines.xhtml
